# Evidence for music therapy and music medicine in psychiatry: transdiagnostic meta-review of meta-analyses

**DOI:** 10.1192/bjo.2024.826

**Published:** 2024-12-13

**Authors:** Alexander Lassner, Spyridon Siafis, Emanuel Wiese, Stefan Leucht, Susanne Metzner, Elias Wagner, Alkomiet Hasan

**Affiliations:** Department of Psychiatry, Psychotherapy and Psychosomatics, Medical Faculty, University of Augsburg, Germany; Department of Psychiatry and Psychotherapy, TUM School of Medicine and Health, Technical University of Munich, Germany; and DZPG (German Center for Mental Health), University of Augsburg, Germany; Faculty of Philosophy and Social Sciences/Leopold Mozart College of Music, University of Augsburg, Germany; Department of Psychiatry, Psychotherapy and Psychosomatics, Medical Faculty, University of Augsburg, Germany; and Section of Evidence-based Psychiatry and Psychotherapy, Medical Faculty, University of Augsburg, Germany; Department of Psychiatry, Psychotherapy and Psychosomatics, Medical Faculty, University of Augsburg, Germany; and DZPG (German Center for Mental Health), University of Augsburg, Germany

**Keywords:** Music therapy, music-based, music medicine, transdiagnostic, meta-review

## Abstract

**Background:**

Music therapy is a commonly used intervention added to usual care for psychiatric disorders.

**Aims:**

We review the evidence for music therapy and assess its efficacy as an adjunct therapy across psychiatric disorders.

**Method:**

A systematic literature search was conducted in four scientific databases to identify relevant meta-analyses. Articles were assessed with the AMSTAR-2 tool. The results of the high-quality articles were recalculated with the data from the primary studies. We decided to add the results of the lower-rated articles, using a narrative approach. We pooled the primary studies and calculated standardised mean differences (SMD) for the transdiagnostic outcomes of depression, anxiety and quality of life. We used the Grading of Recommendations, Assessment, Development and Evaluations (GRADE) tool to assess the level of evidence.

**Results:**

Meta-analyses were available for autism, dementia, depression, insomnia, schizophrenia and substance use disorders. We identified 40 relevant articles. One article per domain was identified as high quality. Music therapy added to treatment as usual showed therapeutic value in each disorder. The transdiagnostic results showed a positive effect of music therapy on depression (SMD = 0.57, 95% CI 0.36–0.78), anxiety (SMD = 0.47, 95% CI 0.27–0.66) and quality of life (SMD = 0.47, 95% CI 0.24–0.71). However, these effects were not maintained at follow-up, and all results were based on low or very low evidence.

**Conclusions:**

Music therapy shows promising potential as an adjunctive treatment for psychiatric disorders, but methodological weaknesses and variability limit the evidence. More high-quality, well-powered studies are needed to reliably confirm its effect size.

The burden of mental illness on societies worldwide is increasing.^1^ Nearly a fifth of the population experiences a psychiatric disorder each year. Over the course of a lifetime, nearly 30% will experience psychiatric symptoms,^2^ with prevalence appearing even higher in Western cultures^3–5^ compared with Africa^6^ or Asia.^7^ In particular, the prevalence of psychiatric disorders increases in old age. Given the demographic changes in Western societies, the elderly will be an important area of psychiatric care in the future.^8,9^ Consequently, the healthcare system needs effective and cost-efficient treatment options that improve quality of life, are low threshold and have few side-effects – therapies that include music seem to be a promising option.

Exposure to sound begins in the prenatal period and triggers important psychological growth processes.^10,11^ Across the lifespan, music plays a major role, from musical elements in early parent–child interactions,^12,13^ to gaining a sense of agency and building sociality in music peer groups (especially in adolescence),^14^ being comforted during life crises^15^ and participation in musical culture.^16,17^ In addition, neuronal, physiological and endocrinological processes are involved in listening to or making music.^18–20^ In everyday life, we listen to music for pleasure and reward,^21^ to regulate our emotions and to cope with stress.^22,23^ The importance of music for health and well-being is now being researched worldwide.^24^ In the context of psychiatric disorders – the focus of this review – music can be a gateway to emotions, memories based on early experiences, social interaction and participation.^25,26^

Music therapy is a common way of treating psychiatric disorders, mainly as an adjunct to standard treatment in the context of in-patient psychiatric care.^27^ A large survey study of professional music therapists worldwide was conducted by the World Federation of Music Therapy in 2017. Most of the respondents (46.6%) were employed in a hospital, mainly in mental health settings (16.7%). Respondents served over 46 specific populations, 11 of which are subsumed under mental healthcare. These included individuals with autism spectrum disorder (44.2%), depressive disorder (31.0%), anxiety disorder (26.9%), trauma- and stressor-related disorders (19.9%), bipolar or related disorders (18.0%) and schizophrenia spectrum disorder (17.9%).^28^ Moreover, one scoping review indicates that music therapy in a psychiatric setting is studied in many countries all around the world (with an emphasis in the USA and Europe), indicating a broad use of this intervention.^29^

There is a wide range of possible therapeutic concepts.^30^ Music therapy is defined as ‘a reflexive process wherein the therapist helps the client to optimize the client's health, using various facets of music experience and the relationships formed through them as the impetus for change. As defined here, music therapy is the professional practice component of the discipline, which informs and is informed by theory and research’.^31^

Among the multitude of music therapy methods, two main categories can be distinguished: active music therapy and receptive music therapy. Active music therapy is based on the alternate or simultaneous production of music in a co-creative process in which emotions can be expressed or perceived. Receptive music therapy uses listening to music to explore the emotional processes during the experience. Both concepts involve building a relationship with the therapist or other group members through the shared experience of music and the subsequent reflective conversation. In contrast, music medicine uses music, emphasising its unique properties primarily as a method to stimulate psychophysical processes like relaxation or anxiety reduction. Methodically, the patient listens to self-selected or prescribed music with or without medical staff guidance.^32–34^ In light of this complexity, recent efforts like the National Institutes of Health Music-Based Intervention Toolkit (MBI) aim to standardise these interventions and support their examination in well-designed trials.^35^

## Objectives

There is no consensus on whether music therapy as an umbrella term or specific music therapy methods have disorder-specific effects, or whether the general efficacy factors of psychotherapy dominate,^36^ nor is there any information on the health economic benefits. A transdiagnostic review of the evidence for music therapy for psychiatric disorders promises to add to the knowledge base, given the changing and/or multiple diagnoses in the course of individual illnesses and the increasing importance of dimensional rather than categorical diagnostic criteria for the selection and implementation of therapies. In addition, it is important to identify research desiderata for specific disorders or symptoms, and to make suggestions for future research.

## Method

We conducted a review of reviews, in accordance with the Preferred Reporting Items for Overviews of Reviews (PRIOR) guidelines (see Supplementary Appendix 1 available at https://doi.org/10.1192/bjo.2024.826).^37^ The meta-review protocol was registered on International Prospective Register of Systematic Reviews (PROSPERO) (identifier CRD42021262593). For protocol deviations, see Supplementary Appendix 2. All analyses are based on previously published studies, and therefore no ethical approval was required.

### Search

We systematically searched the databases PsycINFO, Medline, PubMed and Cochrane Reviews on 20 February 2023 (update; initial search on 28 July 2021). We used a search term organised by the PICOS scheme (population, intervention, comparator, outcome and study design; see Supplementary Appendix 3), to identify suitable meta-analyses. The entire search term is shown in Supplementary Appendix 2. The literature search and selection of articles was done by two independent authors (A.L. and E. Wiese). If no agreement was found, a third author was consulted (A.H. or E. Wagner). Titles and abstracts were reviewed with the Rayyan tool used on Google Chrome (Rayyan, Cambridge, Massachusetts, USA; https://www.rayyan.ai/).^38^ Subsequently, a selection was made at the level of the full text.

### Eligibility criteria

We formulated the inclusion criteria *a priori,* as follows. All meta-analysis based on a systematic literature search, with no restriction regarding publication date, examining music therapy or music medicine in the context of a psychiatric disorder, were the subject of the investigation. The participants had to have a psychiatric diagnosis oriented on the ICD/DSM scheme or exceed a cut-off of a clinician-rated, commonly used, validated questionnaire (e.g. Beck Depression Inventory, Hamilton Rating Scale for Depression, Positive and Negative Syndrome Scale (PANSS) and the State-Trait Anxiety Inventory). We included all comparators (placebo, treatment as usual (TAU), other interventions, other control conditions). All end-points were relevant to the analysis. Special emphasis was paid to changes in specific symptoms of psychiatric disorders, changes in psychopathology, changes in symptom severity, changes in quality of life and general level of functioning. We only considered articles written in English or German.

### Quality assessment

We used the Assessing the Methodological Quality of Systematic Reviews 2 (AMSTAR-2) checklist^39^ to evaluate the articles in terms of methodological quality. The rating table is shown in Supplementary Appendix 6. Two reviewers (A.L. and E. Wiese) rated the articles, and disagreements were solved by a third reviewer (E. Wagner or S.S.).

### Data extraction

Data extraction began on 1 December 2021. In the first step, we extracted all outcomes and effect sizes with measures of heterogeneity of the meta-analyses and the AMSTAR-2 rating. In the second step, we concentrated on the high-quality studies and extracted the aggregate data of the original studies included in the meta-analysis and the risk-of-bias assessments (e.g. Cochrane Risk-of-Bias tool for randomised trials). All data were extracted by A.L. and E. Wiese (see Supplementary Appendix 7). Ambiguities and issues were discussed with E. Wagner and S.S.

### Data analysis and synthesis

The following approach was used to synthesise the data. We focused on the comparison of music therapy plus TAU versus TAU alone. We resynthesised the evidence of studies from high-quality meta-analyses, using standardised mean differences (SMDs) for continuous outcomes and odds ratios for dichotomous outcomes. We harmonised the direction of the effects so that an SMD > 0 and odds ratio >1 indicated outcomes in favour of the intervention. We used a random-effects meta-analysis model,^40^ such that SMDs could be meaningfully compared. This approach allowed for a more comprehensive synthesis in a standardised and uniform scheme.

In our meta-analysis, we combined all time points to obtain an overall effect size of music therapy for the specific outcome variable. Sometimes more than one control condition was available. In this case, we included both arms in the meta-analysis if the control conditions met our definition of TAU (e.g. reading in dementia, which is an activity of daily living). If more than one time point was available, we extracted the longest, to avoid the duplication of data (we did the same for follow-up data). We preferred clinician-rated over self-reported data, the PANSS to the Brief Psychiatric Rating Scale (BPRS), and the Global Assessment of Functioning (GAF) to other functioning scales when available. Data for the general level of functioning (e.g. social functioning, general functioning) were combined into one variable. Heterogeneity was quantified with the *I*^2^-statistic. The Grading of Recommendations, Assessment, Development and Evaluation (GRADE) approach was used to assess the level of evidence (see GRADE criteria in Supplementary Appendix 2).

In addition, we presented the results of low-quality meta-analyses in a narrative form to compare and complement their results with our current meta-analysis. In the narrative report, we focused on articles with the most recent publication date, the largest body of primary studies and the least overlap with the high-quality meta-analyses or report on specific populations.

### Outcomes

We considered all outcomes in the respective meta-analysis. Primary outcomes were the disorder-specific symptoms (e.g. psychotic symptoms in schizophrenia, depressive symptoms in depression, etc.). Secondary outcomes were specific symptom domains, drop-out, functioning, quality of life and adverse events. In a *post hoc* analysis, we combined depressive symptoms, anxiety symptoms and quality of life across diagnosis, to provide a more comprehensive synthesis.

## Results

**Fig. 1:**
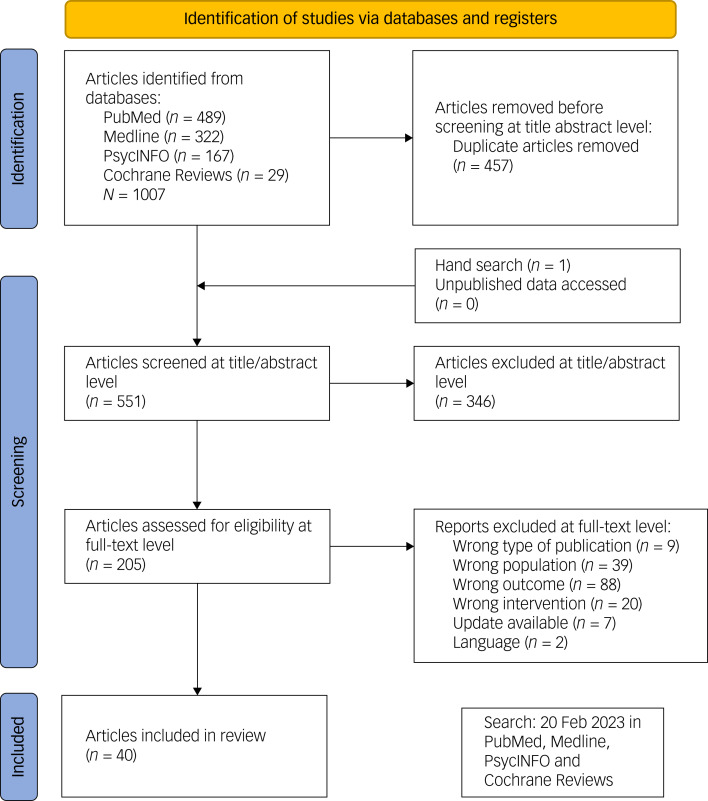
Study flowchart.

A total of 1007 studies were identified by the search algorithm (see Supplementary Appendix 2). By removing duplicates, we excluded 457 items. We added one article by hand searching the reference lists. At title and abstract level, we excluded 346 articles. We examined the remaining 205 articles at the full-text level for inclusion criteria. We had to exclude a further 165 articles on closer examination because they did not meet at least one inclusion criterion. Therefore, 40 articles remained to be included in the meta-review. A flowchart of the selection process is shown in Fig. 1. See Supplementary Appendix 4 for a table of all included and excluded articles.

### Description of studies

Of the 40 articles, 30 were pairwise meta-analyses and ten were network meta-analyses. The articles were very heterogeneous in terms of quality. Only 15% showed a high AMSTAR-2 rating. The remaining 85% were of low (27.5%) or critically low (55%) quality, or could not be rated (2.5%). See Supplementary Appendix 6 for the AMSTAR-2 table.

### Autism

There were three articles that focused on music therapy in relation to autism spectrum disorders. Geretsegger et al^41^ was the only high-quality article according to AMSTAR-2. They included 26 randomised controlled trials and controlled clinical trials with 1165 patients, mostly (21 trials) children between 2 and 12 years of age, with different severity of autism; the remaining samples included adolescents and young adults. Only music therapy interventions by a music therapist were included. Music therapy was provided in a variety of settings, including the home, hospital, out-patient clinics and summer camps. The interventions ranged from 3 days to 8 months (follow-up: 3 days to 12 months). We recalculated the meta-analysis of the primary studies (see the Method section for the exact procedure).

At the end of the intervention, music therapy resulted in a statistically significantly large reduction in overall symptom severity (SMD = 0.83, 95% CI 0.24–1.41, *k* = 9, *n* = 575, GRADE: very low) and increased the odds of global improvement (odds ratio 1.60, 95% CI 1.12–2.28, *k* = 8, *n* = 583, GRADE: low) compared with placebo or TAU. In addition, music therapy improved social interaction (SMD = 0.31, 95% CI 0.01–0.61, *k* = 14, *n* = 627, GRADE: very low) and there was a small increase in quality of life (SMD = 0.32, 95% CI 0.04–0.60, *k* = 3, *n* = 340, GRADE: low). There were no significant changes in follow-up outcomes, except for one variable. Identity formation did not change at the end of treatment, but showed an improvement at follow-up (SMD = 0.86, 95% CI 0.16–1.55, *k* = 1, *n* = 35, GRADE: very low). Music therapy did not change adaptive behaviour, depression, family relationships, verbal or non-verbal communication, and showed no effect on adverse events.

In the lower-rated articles (AMSTAR-2), the findings were similar. A recent meta-analysis was largely based on the same primary studies (Ke et al;^42^ seven out of the eight analysed), and Whipple^43^ combined different outcome domains in the meta-analysis (behaviour, cognition, communication) – an approach that would no longer be the first choice today. See Supplementary Appendix 5 for forest plots and all calculations in detail.

### Dementia and mild cognitive impairment

There were 19 articles dealing with dementia and mild cognitive impairment. Van der Steen et al^44^ was the only article with a high AMSTAR-2 rating. They included 21 studies with 890 participants in their meta-analysis. The participants lived in institutional settings (nursing/residential homes, wards) and had dementia (any type) of varying degrees of severity. The music therapy interventions used active and receptive elements. Interventions were mostly delivered by accredited music therapists (12 studies out of 21), the rest were unclear (four studies) or by other staff (e.g. psychologists, music teachers, research assistants; five studies). Interventions ran from six to 156 sessions, with a median of 14 sessions. A session lasted from 30 min to 2 h. Most trials used an active control condition, typically the promotion of activities of daily living (reading, cooking, board games, etc.), which are usually part of TAU. We recalculated the results of the primary studies of this article. See the Method section for the exact procedure.

At the end of the intervention, music therapy interventions showed an improvement in overall behaviour problems (SMD = 0.24, 95% CI 0.01–0.46, *k* = 13, *n* = 442, GRADE: very low) and social behaviour (SMD = 0.54, 95% CI 0.06–1.02, *k* = 3, *n* = 70, GRADE: very low). In addition, music therapy interventions improved emotional well-being/quality of life (SMD = 0.32, 95% CI 0.02–0.62, *k* = 11, *n* = 348, GRADE: very low) and reduced anxiety (SMD = 0.43, 95% CI 0.13–0.72, *k* = 15, *n* = 478, GRADE: very low) and depression (SMD = 0.27, 95% CI 0.09–0.45, *k* = 12, *n* = 503, GRADE: very low), compared with TAU. The interventions did not show a significant effect on agitation, aggression or cognition. All follow-up outcomes showed no significant results.

The results of the lower-rated articles are listed below (low or critically low). Music therapy had a positive effect on the overall dementia symptoms.^45,46^ There are conflicting results about the effect on cognition, with some meta-analyses finding an effect^45,47–52^ and others not.^53–55^ Most meta-analyses found a positive effect of music therapy on anxiety,^55–57^ depression^45,58–60^ and behaviour/aggression.^55–57,61^ See Supplementary Appendix 5 for forest plots and all calculations in detail.

### Depression

There were five articles dealing with depression. One of these articles received a high AMSTAR-2 rating.^62^ Aalbers et al^62^ (AMSTAR-2: high) reported on nine studies with 411 participants (adolescents and adults, 14–86 years of age) included in the meta-analysis. Participants were recruited through mental health services, nursing homes or high schools. Different music therapy approaches were used. Accredited music therapists provided the therapy in four studies. In the remaining studies, it was not clear whether music therapists were involved, but trained therapists (counsellors, healthcare professionals) were always mentioned. The interventions lasted from 6 to 12 weeks, with a total number of sessions ranging from eight to 48. A single session lasted from 20 to 120 min. We recalculated the results with the primary studies of this article. See the Method section for the exact procedure.

Music therapy (active and receptive, up to 3 months) with TAU significantly improved depression symptoms compared with TAU alone (clinician-rated and patient-rated, SMD = 0.97, 95% CI 0.37–1.57, *k* = 6, *n* = 274, GRADE: very low). In addition, music therapy plus TAU could significantly improve anxiety symptoms (SMD = 0.7, 95% CI 0.01–1.39, *k* = 3, *n* = 192, GRADE: very low). Music therapy did not show significant effects compared with other psychological interventions. There were no significant differences in general functioning, quality of life, self-esteem, drop-out rate or adverse events.

The lower-rated (AMSTAR-2) articles focused on different age groups: children (6–12 years of age), adolescents (13–18 years of age), adults (18–65 years of age) and older adults (≥60 years of age).

A more recent meta-analysis of music-based interventions^63^ (AMSTAR-2: critically low), with an overlap (five out of nine) in primary studies with Aalbers et al,^62^ examined adults and found a similarly high effect size in the improvement of depression compared with the control conditions (TAU/no intervention/active control). Music medicine also showed a significant reduction in depression.

Two network meta-analyses^64,65^ examined the treatment of older adults with depression. Music-based interventions (mostly listening to music) showed the highest improvements in depression symptoms and the highest probability of improvement compared with TAU^64^ (AMSTAR-2: critically low). Dhippayom et al^65^ (AMSTAR-2: critically low) conducted a network meta-analysis of 15 randomised controlled trials with 1144 older adults (≥60 years of age), to evaluate different modalities of music therapy for depression. Six of the trials included participants with dementia. The authors divided the trials according to dose (high: >60 min per week; low: ≤60 min per week), theme (active, receptive, music medicine) and provider (music therapist, non-music therapist). High doses of music therapy delivered by a trained music therapist had the greatest effect on depression. Other types of music therapy also showed significant results.

Geipel et al^66^ (AMSTAR-2: low) reported on five studies involving 195 children and adolescents with internalising symptoms (depression and anxiety). Music therapy significantly reduced internalising symptoms compared with control conditions (various: TAU, waitlist, educational programmes, activities). See Supplementary Appendix 5 for forest plots and all calculations in detail.

### Schizophrenia and psychosis

Our search found six studies addressing schizophrenia and psychosis; one had a high AMSTAR-2 rating.^67^ Geretsegger et al^67^ (AMSTAR-2: high) conducted a meta-analysis of 18 studies with 1215 patients (schizophrenia/schizophrenia-like disorders). All participants were diagnosed with schizophrenia or related psychosis. Active and receptive music therapy concepts were included. The setting was mainly in-patient, with two studies including out-patients. The duration of the interventions ranged from 1 to 6 months. Many different therapeutic approaches were used, with active and receptive elements. Unfortunately, in many trials it is unclear whether an accredited music therapist delivered the intervention. The results of the primary studies were recalculated combining questionnaires and time points. For the exact approach, see the Method section.

Music therapy added to TAU had a significant effect on the general symptoms of schizophrenia (PANSS/BPRS: SMD = 1.09, 95% CI 0.03–2.15, *k* = 5, *n* = 334, GRADE: very low) and on the negative symptoms (Scale for the Assessment of Negative Symptoms: SMD = 0.56, 95% CI 0.17–0.95, *k* = 7, *n* = 443, GRADE: very low). However, there was no significant effect on positive symptoms. Music therapy added to TAU had a positive influence on psychiatric symptoms such as depression (SMD = 0.68, 95% CI 0.36–0.99, *k* = 3, *n* = 165, GRADE: low) and anxiety (SMD = 0.61, 95% CI 0.09–1.13, *k* = 1, *n* = 60, GRADE: low). Music therapy could improve behaviour (SMD = 1.14, 95% CI 0.26–2.02, *k* = 3, *n* = 162, GRADE: very low) and had a positive effect on attention (SMD = 0.72, 95% CI 0.22–1.21, *k* = 1, *n* = 67, GRADE: low) and memory (SMD = 0.5, 95% CI 0.14–0.85, *k* = 2, *n* = 127, GRADE: low), but not on vigilance. Additionally, music therapy added to TAU heightened the perceived support (SMD = 0.73, 95% CI 0.26–1.21, *k* = 1, *n* = 72, GRADE: low). Furthermore, there were no significant differences in the general level of functioning, abstract thinking, quality of life, general response to treatment, satisfaction with treatment or drop-out rate.

A recent meta-analysis^68^ (AMSTAR-2: low) is also worth reporting, as only seven of 18 studies overlap with the high-rated AMSTAR-2 study.^67^ The results also indicate a positive (albeit smaller) effect of music therapy plus TAU on general schizophrenia symptoms compared with TAU (BPRS/PANSS). A similarly large effect of music therapy on negative symptoms was also found.

Other recent meta-analyses with lower AMSTAR-2 ratings covering the topic of music therapy and schizophrenia used only or mostly^69–71^ the same primary studies, or are outdated and use primary studies without control groups.^72^ See Supplementary Appendix 5 for forest plots and all calculations in detail.

### Sleep disorder

Four articles addressed sleep disorders, one of which received a high AMSTAR-2 rating. Jespersen et al^73^ (AMSTAR-2: high) reported on 13 studies involving 1007 patients with insomnia. This meta-analysis included different conditions of insomnia (age-related, arising from a medical condition, pregnancy-related and insomnia disorder). The therapeutic interventions were based on listening to music (researcher or self-selected). The control condition consisted of no intervention or TAU. The daily music listening sessions (3–90 days) ranged from 25 to 60 min (with a mean of 36 min). We focus on reporting the results for the insomnia disorder subgroup.

Music therapy improved sleep quality (SMD = 0.69, 95% CI 0.18–1.2, *k* = 2, *n* = 63, GRADE: very low) compared with the control group. There were also significant improvements in depression (SMD = 3.34, 95% CI 2.18–4.49, *k* = 1, *n* = 30, GRADE: very low) and quality of life (SMD = 0.87, 95% CI 0.16–1.58, *k* = 1, *n* = 34, GRADE: very low). In terms of a sensitivity analysis, the results can be compared with sleep disorders with different forms of insomnia. Sleep disorders arising from any kind of medical condition (SMD = 0.86, 95% CI 0.54–1.19, *k* = 10, *n* = 708, GRADE: very low) showed about the same improvement in sleep quality as primary insomnia.

We briefly mention the noteworthy results of other articles that received a low AMSTAR-2 rating. A meta-analysis^74^ (AMSTAR-2: low) with almost no overlap in primary studies with Jespersen et al^73^ compared acute and chronic sleep disorders. Listening to music significantly improved sleep quality compared with the control condition (similar strength of effect); the effect of listening to music was stronger for people with acute sleep disorders than for people with chronic sleep disorders. In a network meta-analysis,^75^ (AMSTAR-2: low) music-based interventions (e.g. listening to music, music-assisted relaxation, listening to music plus acupuncture, music with exercise) for insomnia disorders were evaluated. Compared with usual care, listening to music alone showed the greatest improvement in sleep quality. In addition, listening to music could improve sleep onset latency. See Supplementary Appendix 5 for forest plots and all calculations in detail.

### Substance use disorder

One article addressed the treatment of substance use disorders. Ghetti et al^76^ (AMSTAR-2: high) included 21 randomised controlled trials with 1984 patients in detoxification settings or longer-term substance use treatment facilities. Of 21 studies, 15 took place in a detoxification setting with a maximum stay of 4–5 days, assessing 1 h of music therapy, and were administered by the same research group. This is why most trials do not show a long course of therapy. In two primary studies, the participants had additional mental health diagnoses. In this analysis, numerous active and passive music therapy concepts are combined for the meta-analysis. We recalculated the meta-analysis with the data from the primary studies. See the Method section for the exact procedure.

Compared with TAU, the adjunct music therapy could show significant improvements in two outcome domains. A medium effect on substance craving (SMD = 0.66, 95% CI 1.23–0.10, *k* = 3, *n* = 254, GRADE: very low) and a small-to-medium effect on motivation for treatment/change (SMD = 0.41, 95% CI 0.21–0.61, *k* = 5, *n* = 408, GRADE: low) could be found in favour of adjunct music therapy compared with TAU. More than 1 h of music therapy was associated with greater reduction in substance craving. The effect of music therapy on motivation for treatment/change was even retained when comparing music therapy with an active control (SMD = 0.46, 95% CI 0.00–0.93). There was no clear evidence regarding the variables depression, anxiety, motivation to stay sober or retention in treatment, compared with TAU or an active control. It is questionable whether the clearly overestimated effect sizes reported for this 1-h intervention go beyond distraction. See Supplementary Appendix 5 for forest plots and all calculations in detail.

### Transdiagnostic results

**Fig. 2:**
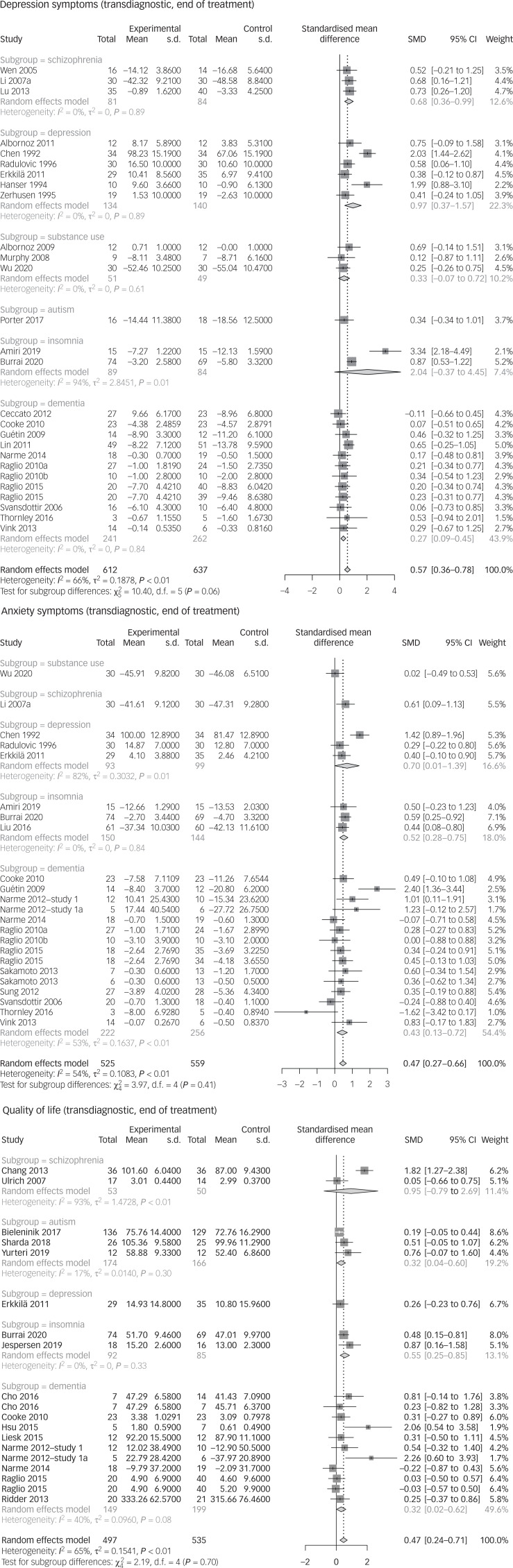
Transdiagnostic calculations of the end-of-treatment data. See the references of the primary studies in Supplementary Appendix 8. SMD, standardised mean difference.

**Fig. 3:**
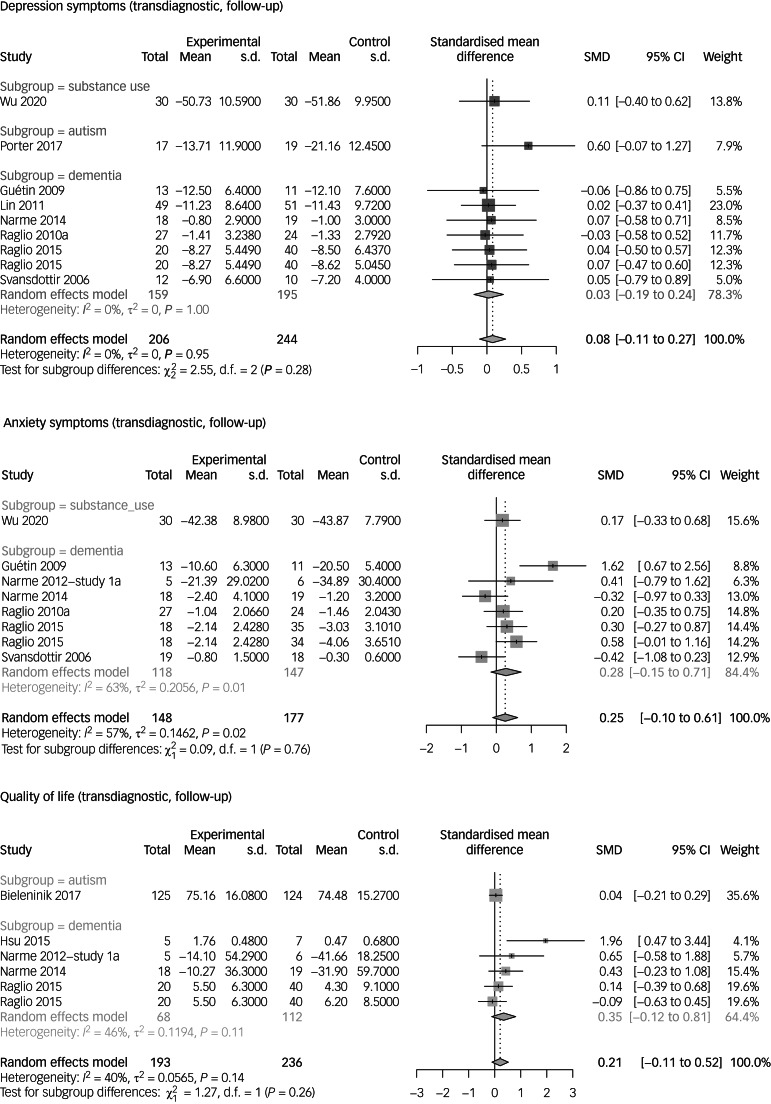
Transdiagnostic calculations of the follow-up data. See the references of the primary studies in Supplementary Appendix 8. SMD, standardised mean difference.

We present the results of a meta-analysis with all available data of the high-rated meta-analyses across diagnoses. See Fig. 2 for forest plots at end of treatment and Fig. 3 for forest plots at follow-up.

At the end of the intervention, music-based interventions achieved an improvement in depressive symptoms (SMD = 0.57, 95% CI 0.36–0.78, *k* = 27, *n* = 1249, GRADE: very low). At follow-up, there was no difference compared with the control group (SMD = 0.08, 95% CI −0.11 to 0.27, *k* = 9, *n* = 450, GRADE: very low). Anxiety symptoms were positively influenced by music-based interventions at the end of treatment (SMD = 0.47, 95% CI 0.27–0.66, *k* = 23, *n* = 1084, GRADE: very low). This effect was not maintained at follow-up (SMD = 0.25, 95% CI −0.1 to 0.61, *k* = 8, *n* = 325, GRADE: very low). Music-based interventions also had a positive effect on quality of life at the end of treatment (SMD = 0.47, 95% CI 0.24–0.71, *k* = 19, *n* = 1032, GRADE: very low). This effect was not found at follow-up (SMD = 0.21, 95% CI −0.11 to 0.52, *k* = 6, *n* = 429, GRADE: very low).

Also worth mentioning is a finding on the dose–response relationship. There is a meta-analysis that explicitly addresses the dose–response relationship of music therapy.^77^ The authors included people with serious mental disorders (psychotic and non-psychotic), defined by severe role disability (low general functioning or in-patient treatment), with all diagnoses included. The dosage of music therapy was the best predictor of its effects, explaining >70% of the variance. For most variables, a linear dose–response relationship provided a good fit. For negative symptoms and functioning, a non-linear relationship, using the square root of the number of sessions, seemed to provide a better fit (indicating a steeper increase in the first few sessions). Small effects can be estimated after three to ten sessions, medium effects occur after ten to 24 sessions and large effects after 16–51 sessions.

## Discussion

In this review of reviews, we summarise, for the first time, the body of evidence for music-based interventions across diagnoses added to TAU. Among other reviews of reviews,^78–80^ this is the first article that makes transdiagnostic calculations and addresses only psychiatric disorders. The included meta-analyses show that music therapy can be offered as a complementary therapy for several psychiatric disorders (autism, dementia, depression, schizophrenia/psychosis, insomnia and substance use disorders), and that therapeutically relevant effects may be achieved. Music therapy appears to be an intervention that may improve quality of life and reduce psychiatric symptoms in specific diagnoses and overlapping diagnoses (anxiety and depression).

First, we conducted a systematic literature search to identify all meta-analyses that investigated music-based interventions for psychiatric disorders. Second, we assessed the meta-analyses, using the standardised AMSTAR-2 tool (15% high quality; one article per diagnosis; see Supplementary Appendix 6). Next, to describe the body of evidence, we used the data from the primary studies of the high-rated articles to repeat the calculations in a standardised form (see Table 1 for a summary of results and Supplementary Appendix 5 for forest plots), supplemented with evidence from the lower-rated articles. In addition, we used the data from the primary studies of the high-quality articles, to perform transdiagnostic calculations for quality of life, anxiety symptoms and depression symptoms (see Figs 2 and 3). Finally, the level of evidence was assessed with GRADE criteria (see Supplementary Appendix 5).

**Table 1:** Summary of results

Disorder	Type of music therapy	Summary of significant results	Calculations based on
Autism	Mostly active music therapy	Autism symptoms	Bharathi 2019, Bieleninik 2017, Chen 2010/2013, Huang 2015, LaGasse 2014, Mateos-Moreno 2013, Rabeyron 2020, Yurteri 2019
		Response to therapy	Bharathi 2019, Bieleninik 2017, Kim 2008, LaGasse 2014, Porter 2017, Rabeyron 2020, Schwartzberg 2013, Thompson 2014
		Quality of life	Bieleninik 2017, Sharda 2018, Yurteri 2019
		Social interaction	Arezina 2011, Thomas 2003, Bharathi 2019, Bieleninik 2017, Chen 2013, Gattino 2011, Ghasemtabar 2015, Kim 2008, LaGasse 2014, Porter 2017, Rabeyron 2020, Schwartzberg 2013, Sharda 2018, Thompson 2014
		Identity development (follow-up)	Porter 2017
Dementia and mild cognitive impairment	Active, receptive music therapy and music medicine	Behavioural problems	Hsu 2015, Lyu 2014, Narme 2014, Raglio 2010a /2010b, Raglio 2015, Sakamoto 2013, Svansdottir 2006, Thornley 2016, Vink 2013
		Social behaviour	Narme 2012 S1/2012 S1a, Narme 2014
		Depression symptoms	Ceccato 2012, Cooke 2010, Guétin 2009, Lin 2011, Narme 2014, Raglio 2010a/ 2010b/2015, Svansdottir 2006, Thornley 2016, Vink 2013
		Anxiety symptoms	Cooke 2010, Guétin 2009, Narme 2012 S1/2012 S1a/ 2014, Raglio 2010a/2010b/ 2015, Sakamoto 2013a/2013b, Sung 2012, Svansdottir 2006, Thornley 2016, Vink 2013
		Quality of life	Cho 2016, Cooke 2010, Hsu 2015, Liesk 2015, Narme 2012 S1/2012 S1a/ 2014, Raglio 2015, Ridder 2013
Depression	Active, receptive music therapy	Depressive symptoms	Albornoz 2011, Chen 1992, Radulovic 1996, Erkkilä 2011, Hanser 1994, Zerhusen 1995
		Anxiety symptoms	Chen 1992, Radulovic 1996, Erkkilä 2011
Schizophrenia and psychosis	Mostly active music therapy	Symptoms of schizophrenia	Mao 2013, Lu 2013, Wen 2005, Talwar 2006, Yang 1998
		Negative symptoms	He 2005, Mohammadi 2012, Tang 1994, Ulrich 2007, Gold 2013, Qu 2012, Yang 1998
		Depression symptoms	Wen 2005, Li 2007a, Lu 2013
		Anxiety symptoms	Li 2007a
		General behaviour	Fu 2013, Li 2007a, Wang 2013
		Attention	Ceccato 2009
		Memory	Cha 2012, Ceccato 2009
		Perceived support	Chang 2013
Primary insomnia	Music medicine	Sleep quality	Amiri 2019, Jespersen 2019
		Depression symptoms	Amiri 2019, Burrai 2020
		Quality of life	Jespersen 2019
Substance use disorder	Active, receptive music therapy and music medicine	Craving	Silverman 2016b/2017, Eshaghi Farahmand 2020
		Motivation for treatment/ change	Silverman 2012/2014/2015b/ 2021, Murphy 2008
Any disorder	Active, receptive music therapy and music medicine	Depression symptoms	See Figs 2 and 3 (forest plots)
		Anxiety symptoms	
		Quality of life	

This is a list of significant results. All calculations with forest plots can be seen in Supplementary Appendix 5. See the references of the primary studies in Supplementary Appendix 8.

Music therapy interventions may reduce symptoms of depression (SMD = 0.57) and anxiety (SMD = 0.47), and improve quality of life (SMD = 0.47) across diagnoses at the end of treatment. Even when different therapy concepts were used (active, receptive, music medicine), the interventions usually achieved similar effect sizes across diagnoses. The transdiagnostic positive effects on anxiety, depression and quality of life are not maintained at follow-up. These calculations combine different severities of the diagnoses and different age groups.

The results and limitations of the review are discussed below. In an umbrella review we had to deal with the fact that some of the meta-analyses were based on the same primary trials. Therefore, we tried to focus on using the highest-rated (AMSTAR-2) articles for our calculations, and to supplement the results with the results of lower-rated meta-analyses in a narrative way; these were mostly consistent. A recent review looked for articles to update the results of the Cochrane reviews.^79^ Only a few primary studies were found, and none of them were considered high quality. Therefore, it can be assumed that the results presented here represent the current state of research.

Over the past decade, the effect size for psychotherapy and psychopharmacology for the treatment of psychiatric disorders has had to be reduced from 0.5^81^ to 0.35.^82^ Probably, the main reason for this is that larger samples and better conducted trials lead to less bias. Effect sizes >0.5 were mainly associated with a high risk of bias or limited evidence.^82^ Replications of highly cited trials of psychotherapy showed a overestimation of the effect by up to 132%.^83^

The factors of a high risk of bias and a limited body of evidence are also present in music therapy. The effect sizes in this meta-review should be understood as the upper limit of possible effects. A meta-analysis with small samples cannot overcome these problems and runs the risk of overestimating effect sizes.^84^ In addition, meta-analyses based on a pooled sample <800 must be considered imprecise.^85,86^ Therefore, we had to downgrade the level of evidence for most of the results, and rated the level of evidence for all results as low or very low. The low quality of the source data poses an important risk of bias, and the reported high effect sizes must be interpreted with caution and cannot be compared to lower effect sizes, e.g. in pharmacological and cognitive–behavioural therapy studies. Furthermore, the effect size of psychotherapeutic or psychosocial interventions may depend on the severity of the disorder and on age. Neurodegenerative effects, which may be present in both dementia and schizophrenia, also need to be considered as a function of age (especially in long-term evaluations) – circumstances that we cannot address in this meta-review, as long-term effects are understudied for most disorders. In addition, the external validity of a meta-analysis is complicated by the fact that research projects tend to include people with few comorbidities or a more clearly defined diagnosis.^87^

A largely unsolved problem of randomised controlled trial designs in music therapy research is the development of an adequate control design, which is related to the relatively small size of the field as well as to the almost unaffordable effort of recruiting comparable therapies. It is more difficult to test so-called complex interventions, such as psychosocial interventions or psychotherapy including music therapy, compared with interventions that can be well isolated (e.g. administration of psychopharmaceuticals) against a control condition (waiting group or other psychosocial intervention or placebo).

In music therapy, music is an object of perception and thus the point of reference for patient and therapist. Apart from the possible physiological effects, music is used to develop the individual's ability to perceive, experience, symbolise and relate. The reception, production and reproduction of music set intrapsychic and interpersonal processes in motion, and have both a diagnostic and therapeutic function.^88^

Irrespective of whether randomised controlled trials follow more ideal experimental conditions or are pragmatically designed, it would have to be considered, when including control groups, that the effect of music therapy would vary with the degree of ‘activity’ of the control condition. However, this is not a problem unique to music therapy, as meta-analyses of numerous randomised controlled psychotherapy trials show.^82^ In terms of bias, the role of patients’ positive attitudes toward music cannot really be ruled out, as there is no placebo music therapy. Critics might argue that the homogeneity of the overall positive effects is a sign of insufficiently sensitive research methods or inadequate measurement criteria.

Music therapy research has only started to proceed into the so-called competition phase.^89^ Controlled clinical studies comparing different music therapeutic interventions are extremely rare. In the field of mental health, Mössler et al^90^ investigated the predictive value for therapeutic success between musical improvisation and choir singing, and Pedersen et al^91^ compared the effects of music therapy with music listening on negative symptoms in schizophrenia. However, both studies are too small to generate generalisable findings. If one disregards the fact that the sensitivity of the measuring instruments may also have to be questioned, it seems that, according to the current state of knowledge, an approximate equivalence of effectiveness of different music therapy approaches can be assumed, just as in psychotherapy.^92^

Depending on the diagnosis, certain music therapy concepts were investigated in the primary studies. The different approaches consider economic factors and address the specifics of the disorders. Empirical evidence for a distinct indication or contraindication for defined populations has not been provided by research yet. However, the transdiagnostic approach in our review is not only a result of a desideratum, but is in line with clinical practice. Patients suffering from severe mental illness often have multiple diagnoses (e.g. psychosis and addiction, depression and anxiety, etc.), and in-patient groups sometimes consist of mixed diagnoses. There is a tremendous amount of clinical experience needed to decide which form of music therapy is indicated concerning the actual individual case. Music therapists are guided by the current biopsychosocial state of the patient, and flexibly adapt their methods within the context of a multimodal treatment concept. The dosage of confrontative interventions is regarded to be handled with caution, as Dümpelmann and Metzner^93^ explain, using the example of schizophrenic and psychotic disorders. Depressive symptoms, for example, are embedded in disorders of varying complexity and therefore severity, which must be considered in the choice of intervention. In a transdiagnostic approach, overlaps between different diagnoses can be seen not only in the phenomenology of symptoms, but also in risk factors and indications for therapy.

The evidence from the study by Gold et al^77^ of a favourable dose–response relationship is often confirmed by clinical observations. Even if the sustainability of the treatment success is in question, it is an advantage, especially for severely ill patients, if a therapy takes effect relatively quickly, because this raises hope and strengthens adherence. Hannibal et al^94^ demonstrated significantly better treatment adherence in patients with schizophrenia and personality disorders compared with the control group. Patients with low treatment motivation across different diagnostic groups have also been shown to benefit from music therapy.^95^ However, in certain patients, the rapid response to music therapy must again be viewed critically. Metzner et al^96^ found rapid synchronisation performance in free improvisations – one of the assumed effective factors of music therapy – in patients with psychosis with a high symptom burden in the first therapy session, but statistical analysis revealed that it was a predictor for the decline of psychotic symptoms only when initial rhythmic attunement occurred further into the first session. This is only a small detail, but it sheds light on the complexity of dose–response relationships.

In general, there are difficulties in analysing and classifying the results, as the term music therapy often covers many different interventions and therapy concepts. This results in a substantial heterogeneity in the design of music therapy trials. The description of the interventions and the control conditions is often very poor (in trials and in meta-analyses). It should be noted that the term music therapy itself is not problematic, but it is necessary to consider and communicate how exactly the intervention was carried out and what the content of the intervention was.^62,78,80^ In some cases, there is no information in the primary studies on whether the intervention was delivered by a music therapist or not. The description of trials and control conditions should use a standardised reporting system.^97^ How the intervention is implemented in the treatment is a very important factor and should also be addressed.^98^

Future research is needed to better differentiate between therapeutic approaches and therapeutic goals. Only then will it be possible to identify the factors that make music therapy effective and unique. Primary studies are also needed to better target specific populations (severity of the disorder) and specific diagnoses. Some are underrepresented or not studied at all (e.g. bipolar disorder, schizoaffective psychosis, attention-deficit hyperactivity disorder, eating disorders). Adverse effects of music therapy have not yet been investigated – an issue that Strupp et al first flagged in 1994.^99^ Important suggestions have been made to improve the quality of trials and address the problems inherent in psychotherapy and pharmacology research, which are also applicable to music therapy.^87^ In addition, specifically for music therapy, the National Institutes of Health MBI Toolkit offers a comprehensive framework of standardised data elements and outcome measures, providing critical methodological guidance to enhance the rigour and effectiveness of future research on music-based interventions across the lifespan.^35^

In conclusion, we were able to use a systematic meta-review and meta-analysis to map the state of research on music therapy as a complementary intervention compared with standard therapy. We used an innovative approach by combining symptoms across different diagnoses into one meta-analytic calculation. Music therapy may offer unique therapeutic value in terms of symptom reduction and quality of life, but more high-quality, well-powered trials are needed to reliably determine the size of the effect.

## Supporting information

Lassner et al. supplementary material 1 Lassner et al. supplementary material

Lassner et al. supplementary material 2 Lassner et al. supplementary material

Lassner et al. supplementary material 3 Lassner et al. supplementary material

Lassner et al. supplementary material 4 Lassner et al. supplementary material

Lassner et al. supplementary material 5 Lassner et al. supplementary material

Lassner et al. supplementary material 6 Lassner et al. supplementary material

Lassner et al. supplementary material 7 Lassner et al. supplementary material

Lassner et al. supplementary material 8 Lassner et al. supplementary material

Lassner et al. supplementary material 9 Lassner et al. supplementary material

## Data Availability

The data that support the findings of this study are openly available in the Appendix of the original meta-analyses: 10.1002/14651858.CD004517.pub3., 10.1002/14651858.CD004025.pub4., 10.1002/14651858.CD004381.pub4., 10.1002/14651858.CD012576.pub3., 10.1002/14651858.CD010459.pub3., 10.1002/14651858.CD003477.pub4.
