# Supplementary material for: Evidence for music therapy and music medicine in psychiatry: transdiagnostic meta-review of meta-analyses
Source: BJPsych Open. 2024 Dec 13;11(1):e4. doi: 10.1192/bjo.2024.826 (PMC11733488; doi:10.1192/bjo.2024.826)
Supplement: Lassner et al. supplementary material 2 — Lassner et al. supplementary material [file S2056472424008263sup002.docx]

**Appendix 2**

**Search-Term (generic)**

(depression OR depressive OR dysthymia OR mental illness* OR mental disorder* OR mood disorder* OR affective disorder* OR anxiety OR panic disorder OR obsessive compulsive OR OCD OR ADHD OR attention deficit OR attentional deficit OR phobia OR bipolar OR psychosis OR psychotic OR schizophr* OR post traumatic* OR personality disorder* OR stress disorder* OR dissociative disorder* OR dementia OR alzheimer* OR cognitive impairment OR cognitive deficits OR autism OR developmental disorder OR addiction OR substance abuse OR drug abuse OR sleeping disorder*) AND (music therapy OR music based OR music intervention*) AND (random* OR placebo OR control* OR adjunc* OR added* OR addit*) AND (meta-analy* OR metaanaly* OR meta regr* OR metaregr* OR systematic review*)

**Amendments to the protocol**

We have made the following changes to the original protocol. The amendments were made after the AMSTAR-2 rating:

Strategy for data synthesis:

- Since we found only one high-quality (AMSTAR-2) meta-analysis per diagnosis we recalculated the original data, to present it in a standardised way; no data was pooled within a diagnosis.
- In the synthesis of the results, we chose to report the findings of the lower quality meta-analyses in a narrative way.
- We did transdiagnostic calculations and pooled the data of primary studies of the high-rated meta-analyses across diagnoses regarding quality of life, depression and anxiety.

**Grade-Criteria**

The GRADE-rating started from high and could be downgraded to moderate, low or very low according to the following rules for each domain.

- Reporting bias: There was no downgrading due to reporting bias, because the Cochrane reviews did not find any.
- Study limitations: We used the original ratings by the Cochrane reviews. We assigned each study 1 for low risk of bias, 2 for unclear and 3 for high risk. We calculated a weighted risk of bias of the pooled estimate using the weight of the study contributing to the estimate. We downgraded the evidence by 2 levels if the weighted ROB was high (>2.5) and by 1 level if it was moderate (>1.5).
- Heterogeneity: The evidence was downgraded 2 levels if *I^2^*>75% and 1 level if *I*^2^>50%. If there was only one study, we did not downgrade the evidence.
- Imprecision: We followed the guidelines outlined in Nikolakopoulou et al. (2020, CINeMA) establishing a range of equivalence for SMD of -0.1 to 0.1 and OR from 0.83 to 1.2. If we didn't downgrade for this reason, we considered downgrading by one level when the sample size was less than 800 participants.
