# Supplementary material for: Evidence for music therapy and music medicine in psychiatry: transdiagnostic meta-review of meta-analyses
Source: BJPsych Open. 2024 Dec 13;11(1):e4. doi: 10.1192/bjo.2024.826 (PMC11733488; doi:10.1192/bjo.2024.826)
Supplement: Lassner et al. supplementary material 3 — Lassner et al. supplementary material [file S2056472424008263sup003.docx]

| PICOS | Selection criteria |
| --- | --- |
| Population | Patients with a psychiatric diagnosis: e.g., depression, dysthymia, mood disorder, anxiety, panic disorder, obsessive compulsive disorder, ADHD, phobia, bipolar disorder, psychosis, post-traumatic stress disorder, personality disorder, dissociative disorder, dementia, cognitive impairment, autism, addiction, substance use disorder, sleeping disorder |
| Intervention | Music therapy, music-based intervention |
| Comparators | All controls and comparators are relevant |
| Outcomes | Symptom severity, all other outcomes |
| Study design | Meta-analysis of rcts / ccts / qrcts |

PICOS table
