# Supplementary material for: Evidence for music therapy and music medicine in psychiatry: transdiagnostic meta-review of meta-analyses
Source: BJPsych Open. 2024 Dec 13;11(1):e4. doi: 10.1192/bjo.2024.826 (PMC11733488; doi:10.1192/bjo.2024.826)
Supplement: Lassner et al. supplementary material 4 — Lassner et al. supplementary material [file S2056472424008263sup004.docx]

| Appendix Included Studies | |
| --- | --- |
| 1 | Aalbers, S., Fusar-Poli, L., Freeman, R. E., Spreen, M., Ket, J. C. F., Vink, A. C., Maratos, A., Crawford, M., Chen, X.-J., & Gold, C. (2017). Music therapy for depression. Cochrane Database of Systematic Reviews, 11, CD004517. https://doi.org/10.1002/14651858.CD004517.pub3 |
| 2 | Andrews, L. (2016). Music for Insomnia in Adults. Clinical Nurse Specialist, 30(4), 198-199. https://doi.org/10.1097/nur.0000000000000221 |
| 3 | Bian, X., Wang, Y., Zhao, X., Zhang, Z., & Ding, C. (2021). Does music therapy affect the global cognitive function of patients with dementia? A meta-analysis. NeuroRehabilitation, 48(4), 553-562. https://content.iospress.com/articles/neurorehabilitation/nre210018 |
| 4 | Brown Wilson, C., Arendt, L., Nguyen, M., Scott, T. L., Neville, C. C., & Pachana, N. A. (2019). Nonpharmacological Interventions for Anxiety and Dementia in Nursing Homes: A Systematic Review. Gerontologist, 59(6), e731-e742. https://doi.org/10.1093/geront/gnz020 |
| 5 | Burley, C. V., Burns, K., Lam, B. C. P., & Brodaty, H. (2022). Nonpharmacological approaches reduce symptoms of depression in dementia: A systematic review and meta-analysis. Ageing research reviews, 79, 101669. https://doi.org/10.1016/j.arr.2022.101669 |
| 6 | Chang, Y. S., Chu, H., Yang, C. Y., Tsai, J. C., Chung, M. H., Liao, Y. M., Chi, M. J., Liu, M. F., & Chou, K. R. (2015). The efficacy of music therapy for people with dementia: A meta-analysis of randomised controlled trials. J Clin Nurs, 24(23-24), 3425-3440. https://doi.org/10.1111/jocn.12976 |
| 7 | Chen, Y. J., Li, X. X., Pan, B., Wang, B., Jing, G. Z., Liu, Q. Q., Li, Y. F., Bing, Z. T., Yang, K. H., Han, X. M., & Ge, L. (2021). Non-pharmacological interventions for older adults with depressive symptoms: a network meta-analysis of 35 randomized controlled trials. Aging Ment Health, 25(5), 773-786. https://doi.org/10.1080/13607863.2019.1704219 |
| 8 | Dhippayom, T., Saensook, T., Promkhatja, N., Teaktong, T., Chaiyakunapruk, N., & Devine, B. (2022). Comparative effects of music interventions on depression in older adults: A systematic review and network meta-analysis. EClinicalMedicine, 50, 101509. https://doi.org/10.1016/j.eclinm.2022.101509 |
| 9 | Dorris, J. L., Neely, S., Terhorst, L., VonVille, H. M., & Rodakowski, J. (2021). Effects of music participation for mild cognitive impairment and dementia: A systematic review and meta-analysis. J Am Geriatr Soc. https://doi.org/10.1111/jgs.17208 |
| 10 | Feng, F., Zhang, Y., Hou, J., Cai, J., Jiang, Q., Li, X., Zhao, Q., & Li, B. A. (2018). Can music improve sleep quality in adults with primary insomnia? A systematic review and network meta-analysis. Int J Nurs Stud, 77, 189-196. |
| 11 | Fusar-Poli, L., Bieleninik, L., Brondino, N., Chen, X. J., & Gold, C. (2018). The effect of music therapy on cognitive functions in patients with dementia: a systematic review and meta-analysis. Aging Ment Health, 22(9), 1097-1106. https://doi.org/10.1080/13607863.2017.1348474 |
| 12 | Geipel, J., Koenig, J., Hillecke, T. K., Resch, F., & Kaess, M. (2018). Music-based interventions to reduce internalizing symptoms in children and adolescents: A meta-analysis. J Affect Disord, 225, 647-656. https://doi.org/10.1016/j.jad.2017.08.035 |
| 13 | Geretsegger, M., Fusar-Poli, L., Elefant, C., Mössler, K. A., Vitale, G., & Gold, C. (2022). Music therapy for autistic people. Cochrane Database of Systematic Reviews(5). https://doi.org/10.1002/14651858.CD004381.pub4 |
| 14 | Geretsegger, M., Mossler, K. A., Bieleninik, L., Chen, X. J., Heldal, T. O., & Gold, C. (2017). Music therapy for people with schizophrenia and schizophrenia-like disorders. Cochrane Database Syst Rev, 5(5), CD004025. https://doi.org/10.1002/14651858.CD004025.pub4 |
| 15 | Ghetti, C., Chen, X.-J., Brenner, A. K., Hakvoort, L. G., Lien, L., Fachner, J., & Gold, C. (2022). Music therapy for people with substance use disorders. The Cochrane database of systematic reviews, 5, CD012576. https://doi.org/10.1002/14651858.CD012576.pub3 |
| 16 | Gold, C., Solli, H. P., Kruger, V., & Lie, S. A. (2009). Dose-response relationship in music therapy for people with serious mental disorders: systematic review and meta-analysis. Clin Psychol Rev, 29(3), 193-207. https://doi.org/10.1016/j.cpr.2009.01.001 |
| 17 | Gold, C., Voracek, M., & Wigram, T. (2004). Effects of music therapy for children and adolescents with psychpathology: a meta-analysis. Journal of Child Psychology and Psychiatry, 45(6), 1045-1063. https://doi.org/10.1111/j.1469-7610.2004.t01-1-00298.x |
| 18 | Ito, E., Nouchi, R., Dinet, J., Cheng, C.-H., & Husebø, B. S. (2022). The Effect of Music-Based Intervention on General Cognitive and Executive Functions, and Episodic Memory in People with Mild Cognitive Impairment and Dementia: A Systematic Review and Meta-Analysis of Recent Randomized Controlled Trials. Healthcare (Basel, Switzerland), 10(8). https://doi.org/10.3390/healthcare10081462 |
| 19 | Jespersen, K. V., Pando-Naude, V., Koenig, J., Jennum, P., & Vuust, P. (2022). Listening to music for insomnia in adults. Cochrane Database of Systematic Reviews(8). https://doi.org/10.1002/14651858.CD010459.pub3 |
| 20 | Jia, R., Liang, D., Yu, J., Lu, G., Wang, Z., Wu, Z., Huang, H., & Chen, C. (2020). The effectiveness of adjunct music therapy for patients with schizophrenia: A meta-analysis. Psychiatry Res, 293, 113464. https://doi.org/10.1016/j.psychres.2020.113464 |
| 21 | Jin, B., Xv, Y., Zhang, B., Qiao, L., & Liu, H. (2022). Comparative efficacy and acceptability of treatments for depressive symptoms in cognitive impairment: A systematic review and Bayesian network meta-analysis. Frontiers in Aging Neuroscience, 14. https://doi.org/10.3389/fnagi.2022.1037414 |
| 22 | Ke, X., Song, W., Yang, M., Li, J., & Liu, W. (2022). Effectiveness of music therapy in children with autism spectrum disorder: A systematic review and meta-analysis. Frontiers in Psychiatry, 13, 905113. https://doi.org/10.3389/fpsyt.2022.905113 |
| 23 | Lai, X., Wen, H., Li, Y., Lu, L., & Tang, C. (2020). The Comparative Efficacy of Multiple Interventions for Mild Cognitive Impairment in Alzheimer's Disease: A Bayesian Network Meta-Analysis. Front Aging Neurosci, 12, 121. https://doi.org/10.3389/fnagi.2020.00121 |
| 24 | Lee, K. H., Lee, J. Y., & Kim, B. (2020). Person-Centered Care in Persons Living with Dementia: A Systematic Review and Meta-Analysis. Gerontologist. https://doi.org/10.1093/geront/gnaa207 |
| 25 | Li, H. C., Wang, H. H., Lu, C. Y., Chen, T. B., Lin, Y. H., & Lee, I. (2019). The effect of music therapy on reducing depression in people with dementia: A systematic review and meta-analysis. Geriatr Nurs, 40(5), 510-516. https://doi.org/10.1016/j.gerinurse.2019.03.017 |
| 26 | Liang, J. H., Xu, Y., Lin, L., Jia, R. X., Zhang, H. B., & Hang, L. (2018). Comparison of multiple interventions for older adults with Alzheimer disease or mild cognitive impairment: A PRISMA-compliant network meta-analysis. Medicine (Baltimore), 97(20), e10744. https://doi.org/10.1097/MD.0000000000010744 |
| 27 | Liu, Q., Wang, F., Tan, L., Liu, L., Cheng, H., & Hu, X. (2023). Comparative efficacy of various art therapies for patients with dementia: A network meta-analysis of randomized controlled trials. Frontiers in Psychiatry, 14, 1072066. https://doi.org/10.3389/fpsyt.2023.1072066 |
| 28 | Lutgens, D., Gariepy, G., & Malla, A. (2017). Psychological and psychosocial interventions for negative symptoms in psychosis: systematic review and meta-analysis. Br J Psychiatry, 210(5), 324-332. https://doi.org/10.1192/bjp.bp.116.197103 |
| 29 | Na, R., Yang, J. H., Yeom, Y., Kim, Y. J., Byun, S., Kim, K., & Kim, K. W. (2019). A Systematic Review and Meta-Analysis of Nonpharmacological Interventions for Moderate to Severe Dementia. Psychiatry Investig, 16(5), 325-335. https://doi.org/10.30773/pi.2019.02.11.2 |
| 30 | Silverman, M. J. (2003). The influence of music on the symptoms of psychosis: a meta-analysis. J Music Ther, 40(1), 27-40. https://doi.org/10.1093/jmt/40.1.27 |
| 31 | Steenhuis, L. A., Nauta, M. H., Bocking, C. L., & Pijnenborg, G. H. (2015). Treating Depressive Symptoms in Psychosis: A Network Meta-Analysis on the Effects of Non-Verbal Therapies. PLoS One, 10(10), e0140637. https://doi.org/10.1371/journal.pone.0140637 |
| 32 | Tang, Q., Huang, Z., Zhou, H., & Ye, P. (2020). Effects of music therapy on depression: A meta-analysis of randomized controlled trials. PLoS One, 15(11), e0240862. https://doi.org/10.1371/journal.pone.0240862 |
| 33 | Tseng, P. T., Chen, Y. W., Lin, P. Y., Tu, K. Y., Wang, H. Y., Cheng, Y. S., Chang, Y. C., Chang, C. H., Chung, W., & Wu, C. K. (2016). Erratum to: Significant treatment effect of adjunct music therapy to standard treatment on the positive, negative, and mood symptoms of schizophrenic patients: a meta-analysis. BMC Psychiatry, 16, 150. https://doi.org/10.1186/s12888-016-0846-1 |
| 34 | Ueda, T., Suzukamo, Y., Sato, M., & Izumi, S. (2013). Effects of music therapy on behavioral and psychological symptoms of dementia: a systematic review and meta-analysis. Ageing Res Rev, 12(2), 628-641. https://doi.org/10.1016/j.arr.2013.02.003 |
| 35 | van der Steen, J. T., Smaling, H. J., van der Wouden, J. C., Bruinsma, M. S., Scholten, R. J., & Vink, A. C. (2018). Music-based therapeutic interventions for people with dementia. The Cochrane database of systematic reviews, 7, CD003477. https://doi.org/10.1002/14651858.CD003477.pub4 |
| 36 | Wang, C. F., Sun, Y. L., & Zang, H. X. (2014). Music therapy improves sleep quality in acute and chronic sleep disorders: a meta-analysis of 10 randomized studies. Int J Nurs Stud, 51(1), 51-62. https://doi.org/10.1016/j.ijnurstu.2013.03.008 |
| 37 | Wang, Y. Q., Jia, R. X., Liang, J. H., Li, J., Qian, S., Li, J. Y., & Xu, Y. (2020). Effects of non-pharmacological therapies for people with mild cognitive impairment. A Bayesian network meta-analysis. Int J Geriatr Psychiatry, 35(6), 591-600. https://doi.org/10.1002/gps.5289 |
| 38 | Watt, J. A., Goodarzi, Z., Veroniki, A. A., Nincic, V., Khan, P. A., Ghassemi, M., Thompson, Y., Tricco, A. C., & Straus, S. E. (2019). Comparative Efficacy of Interventions for Aggressive and Agitated Behaviors in Dementia: A Systematic Review and Network Meta-analysis. Ann Intern Med, 171(9), 633-642. https://doi.org/10.7326/M19-0993 |
| 39 | Whipple, J. (2004). Music in intervention for children and adolescents with autism: a meta-analysis. J Music Ther, 41(2), 90-106. https://doi.org/10.1093/jmt/41.2.90 |
| 40 | Zhang, Y., Cai, J., An, L., Hui, F., Ren, T., Ma, H., & Zhao, Q. (2017). Does music therapy enhance behavioral and cognitive function in elderly dementia patients? A systematic review and meta-analysis. Ageing Res Rev, 35, 1-11. |

| Appendix Excluded Studies | | |
| --- | --- | --- |
| # | Study | Reason for exclusion |
| 1 | Abraha, I., Rimland, J. M., Lozano-Montoya, I., Dell'Aquila, G., Velez-Diaz-Pallares, M., Trotta, F. M., Cruz-Jentoft, A. J., & Cherubini, A. (2020). Simulated presence therapy for dementia. Cochrane Database Syst Rev, 4(11), CD011882. https://doi.org/10.1002/14651858.CD011882.pub3 | Wrong intervention |
| 2 | Aleixo, M. A. R., Santos, R. L., & do Nascimento Dourado, M. C. (2017). Efficacy of music therapy in the neuropsychiatric symptoms of dementia: Systematic review. Jornal Brasileiro de Psiquiatria, 66(1), 52-61. http://search.ebscohost.com/login.aspx?direct=true&db=psyh&AN=2017-31723-007&site=ehost-live | Wrong outcome |
| 3 | Ang, K., Maddocks, M., Xu, H., & Higginson, I. J. (2017). The Effectiveness of Singing or Playing a Wind Instrument in Improving Respiratory Function in Patients with Long-Term Neurological Conditions: A Systematic Review. Journal of Music Therapy, 54(1), 108-131. https://doi.org/10.1093/jmt/thx001 | Wrong population |
| 4 | Applewhite, B., Cankaya, Z., Heiderscheit, A., & Himmerich, H. (2022). A Systematic Review of Scientific Studies on the Effects of Music in People with or at Risk for Autism Spectrum Disorder. International journal of environmental research and public health, 19(9). https://doi.org/10.3390/ijerph19095150 | Wrong population |
| 5 | Backhouse, T., Dudzinski, E., Killett, A., & Mioshi, E. (2020). Strategies and interventions to reduce or manage refusals in personal care in dementia: A systematic review. International Journal of Nursing Studies, 109, 103640. https://doi.org/10.1016/j.ijnurstu.2020.103640 | Wrong outcome |
| 6 | Baker, F. A., Metcalf, O., Varker, T., & O'Donnell, M. (2018). A systematic review of the efficacy of creative arts therapies in the treatment of adults with PTSD. Psychological Trauma: Theory, Research, Practice, and Policy, 10(6), 643-651. https://doi.org/10.1037/tra0000353 | Wrong outcome |
| 7 | Baker, F. A., Pool, J., Johansson, K., Wosch, T., Bukowska, A. A., Kulis, A., Blauth, L., Stensæth, K., Clark, I. N., & Odell-Miller, H. (2021). Strategies for Recruiting People With Dementia to Music Therapy Studies: Systematic Review. Journal of Music Therapy. https://doi.org/10.1093/jmt/thab010 | Wrong outcome |
| 8 | Baumeister, H., Hutter, N., & Bengel, J. (2012). Psychological and pharmacological interventions for depression in patients with diabetes mellitus and depression. Cochrane Database of Systematic Reviews, 12, CD008381. https://doi.org/10.1002/14651858.CD008381.pub2 | Wrong population |
| 9 | Beck, B. D., Lund, S. T., Søgaard, U., Simonsen, E., Tellier, T. C., Cordtz, T. O., Laier, G. H., & Moe, T. (2018). Music therapy versus treatment as usual for refugees diagnosed with posttraumatic stress disorder (PTSD): study protocol for a randomized controlled trial. Trials, 19(1), 301. https://www.ncbi.nlm.nih.gov/pmc/articles/PMC5977477/pdf/13063_2018_Article_2662.pdf | Wrong publication |
| 10 | Belski, N., Abdul-Rahman, Z., Youn, E., Balasundaram, V., & Diep, D. (2022). Review: The effectiveness of musical therapy in improving depression and anxiety symptoms among children and adolescents - a systematic review. Child and Adolescent Mental Health, 27(4), 369-377. https://doi.org/10.1111/camh.12526 | Wrong outcome |
| 11 | Boehm, K., Cramer, H., Staroszynski, T., & Ostermann, T. (2014). Arts Therapies for Anxiety, Depression, and Quality of Life in Breast Cancer Patients: A Systematic Review and Meta-Analysis. Evidence-Based Complementary and Alternative Medicine, 2014, 1-9. https://doi.org/10.1155/2014/103297 | Wrong population |
| 12 | Booth, V., Hood, V., & Kearney, F. (2016). Interventions incorporating physical and cognitive elements to reduce falls risk in cognitively impaired older adults. JBI database of systematic reviews and implementation reports, 14(5), 110-135. https://doi.org/10.11124/jbisrir-2016-002499 | Wrong intervention |
| 13 | Broder-Fingert, S., Feinberg, E., & Silverstein, M. (2017). Music Therapy for Children With Autism Spectrum Disorder. Jama, 318(6), 523-524. https://doi.org/10.1001/jama.2017.9477 | Wrong outcome |
| 14 | Brondino, N., Fusar-Poli, L., Rocchetti, M., Provenzani, U., Barale, F., & Politi, P. (2015). Complementary and Alternative Therapies for Autism Spectrum Disorder. Evidence-Based Complementary and Alternative Medicine, 2015, 1-31. https://doi.org/10.1155/2015/258589 | Wrong outcome |
| 15 | Brown, L. S., & Jellison, J. A. (2012). Music Research with Children and Youth with Disabilities and Typically Developing Peers: A Systematic Review. Journal of Music Therapy, 49(3), 335-364. https://doi.org/10.1093/jmt/49.3.335 | Wrong population |
| 16 | Buechner, H., Toparlak, S. M., Ostinelli, E. G., Shokraneh, F., Nicholls-Mindlin, J., Cipriani, A., Geddes, J. R., & Syed Sheriff, R. (2023). Community interventions for anxiety and depression in adults and young people: A systematic review. Aust N Z J Psychiatry, 48674221150362. https://doi.org/10.1177/00048674221150362 | Wrong outcome |
| 17 | Bunn, D. K., Abdelhamid, A., Copley, M., Cowap, V., Dickinson, A., Howe, A., Killett, A., Poland, F., Potter, J. F., Richardson, K., Smithard, D., Fox, C., & Hooper, L. (2016). Effectiveness of interventions to indirectly support food and drink intake in people with dementia: Eating and Drinking Well IN dementiA (EDWINA) systematic review. BMC Geriatr, 16, 89. https://doi.org/10.1186/s12877-016-0256-8 | Wrong intervention |
| 18 | Burley, C. V., Burns, K., & Brodaty, H. (2022). Pharmacological and nonpharmacological approaches to reduce disinhibited behaviors in dementia: a systematic review. International Psychogeriatrics, 1-17. https://doi.org/10.1017/S1041610222000151 | Wrong outcome |
| 19 | Campbell, E. A., Kantor, J., Kantorova, L., Svobodova, Z., & Wosch, T. (2021). Tactile Low Frequency Vibration in Dementia Management: A Scoping Review Protocol. Int J Environ Res Public Health, 18(4). https://doi.org/10.3390/ijerph18041904 | Wrong publication |
| 20 | Carter, T. E., & Panisch, L. S. (2020). A systematic review of music therapy for psychosocial outcomes of substance use clients. International Journal of Mental Health and Addiction. http://search.ebscohost.com/login.aspx?direct=true&db=psyh&AN=2020-16925-001&site=ehost-live | Wrong outcome |
| 21 | Castillo-Bueno, M. D., Moreno-Pina, J. P., Martinez-Puente, M. V., Artiles-Suarez, M. M., Company-Sancho, M. C., Garcia-Andres, M. C., Sanchez-Villar, I., & Hernandez-Perez, R. (2010). Effectiveness of nursing intervention for adult patients experiencing chronic pain: a systematic review. JBI Libr Syst Rev, 8(28), 1112-1168. https://doi.org/10.11124/01938924-201008280-00001 | Wrong population |
| 22 | Chan, M. F., Wong, Z. Y., & Thayala, N. V. (2010). A systematic review on the effectiveness of music listening in reducing depressive symptoms in adults. JBI Libr Syst Rev, 8(31), 1242-1287. https://doi.org/10.11124/01938924-201008310-00001 | Wrong population |
| 23 | Chan, M. F., Wong, Z. Y., & Thayala, N. V. (2011). The effectiveness of music listening in reducing depressive symptoms in adults: a systematic review. Complement Ther Med, 19(6), 332-348. https://doi.org/10.1016/j.ctim.2011.08.003 | Wrong population |
| 24 | Chen, C. T., Tung, H. H., Fang, C. J., Wang, J. L., Ko, N. Y., Chang, Y. J., & Chen, Y. C. (2021). Effect of music therapy on improving sleep quality in older adults: A systematic review and meta-analysis. J Am Geriatr Soc, 69(7), 1925-1932. https://doi.org/10.1111/jgs.17149 | Wrong population |
| 25 | Chen, X., Wei, Q., Jing, R., & Fan, Y. (2021). Effects of music therapy on cancer-related fatigue, anxiety, and depression in patients with digestive tumors: A protocol for systematic review and meta-analysis. Medicine (Baltimore), 100(22), e25681. https://doi.org/10.1097/MD.0000000000025681 | Wrong population |
| 26 | Chen, X. J., Leith, H., Aarø, L. E., Manger, T., & Gold, C. (2016). Music therapy for improving mental health problems of offenders in correctional settings: Systematic review and meta-analysis. Journal of Experimental Criminology, 12(2), 209-228. http://search.ebscohost.com/login.aspx?direct=true&db=psyh&AN=2016-09577-001&site=ehost-live | Wrong population |
| 27 | Cheung, D. S. K., Wang, S. S., Li, Y., Ho, K. H. M., Kwok, R. K. H., Mo, S. H., & Bressington, D. (2022). Sensory-based interventions for the immediate de-escalation of agitation in people with dementia: A systematic review. Aging & Mental Health, 1-12. https://doi.org/10.1080/13607863.2022.2116404 | Wrong intervention |
| 28 | Cho, E., Shin, J., Seok, J. W., Lee, H., Lee, K. H., Jang, J., Heo, S.-J., & Kang, B. (2023). The effectiveness of non-pharmacological interventions using information and communication technologies for behavioral and psychological symptoms of dementia: A systematic review and meta-analysis. International Journal of Nursing Studies, 138, 104392. https://doi.org/10.1016/j.ijnurstu.2022.104392 | Wrong intervention |
| 29 | Clark, I., & Harding, K. (2012). Psychosocial outcomes of active singing interventions for therapeutic purposes: A systematic review of the literature. Nordic Journal of Music Therapy, 21(1), 80-98. http://search.ebscohost.com/login.aspx?direct=true&db=psyh&AN=2012-04057-004&site=ehost-live | Wrong outcome |
| 30 | Cogo-Moreira, H., Andriolo, R. B., Yazigi, L., Ploubidis, G. B., Brandao de Avila, C. R., & Mari, J. J. (2012). Music education for improving reading skills in children and adolescents with dyslexia. Cochrane Database Syst Rev(8), CD009133. https://doi.org/10.1002/14651858.CD009133.pub2 | Wrong outcome |
| 31 | Craig, J. (2014). Music therapy to reduce agitation in dementia. Nurs Times, 110(32-33), 12-15. https://www.ncbi.nlm.nih.gov/pubmed/25188964 | Wrong outcome |
| 32 | de Niet, G., Tiemens, B., Lendemeijer, B., & Hutschemaekers, G. (2009). Music-assisted relaxation to improve sleep quality: meta-analysis. J Adv Nurs, 65(7), 1356-1364. https://doi.org/10.1111/j.1365-2648.2009.04982.x | Wrong population |
| 33 | de Niet, G. J., Tiemens, B. G., Kloos, M. W., & Hutschemaekers, G. J. (2009). Review of systematic reviews about the efficacy of non-pharmacological interventions to improve sleep quality in insomnia. Int J Evid Based Healthc, 7(4), 233-242. | Wrong publication |
| 34 | Deshmukh, S. R., Holmes, J., & Cardno, A. (2018). Art therapy for people with dementia. Cochrane Database Syst Rev, 9(9), CD011073. https://doi.org/10.1002/14651858.CD011073.pub2 | Wrong outcome |
| 35 | Dolle, K., & Schulte-Korne, G. (2014). [Complementary treatment methods for depression in children and adolescents]. Prax Kinderpsychol Kinderpsychiatr, 63(3), 237-263. https://www.ncbi.nlm.nih.gov/pubmed/24707770 (Komplementare Ansatze zur Behandlung von depressiven Storungen bei Kindern und Jugendlichen.) | Wrong outcome |
| 36 | Dyer, S. M., Harrison, S. L., Laver, K., Whitehead, C., & Crotty, M. (2018). An overview of systematic reviews of pharmacological and non-pharmacological interventions for the treatment of behavioral and psychological symptoms of dementia. Int Psychogeriatr, 30(3), 295-309. https://doi.org/10.1017/S1041610217002344 | Wrong publication |
| 37 | Edwards, J. (2006). Music therapy in the treatment and management of mental disorders. Ir J Psychol Med, 23(1), 33-35. https://doi.org/10.1017/S0790966700009459 | Wrong outcome |
| 38 | Fakhoury, N., Wilhelm, N., Sobota, K. F., & Kroustos, K. R. (2017). Impact of Music Therapy on Dementia Behaviors: A Literature Review. Consult Pharm, 32(10), 623-628. https://doi.org/10.4140/TCP.n.2017.623 | Wrong outcome |
| 39 | Fischer, R., Bortolini, T., Karl, J. A., Zilberberg, M., Robinson, K., Rabelo, A., Gemal, L., Wegerhoff, D., Nguyen, T. B. T., Irving, B., Chrystal, M., & Mattos, P. (2020). Rapid Review and Meta-Meta-Analysis of Self-Guided Interventions to Address Anxiety, Depression, and Stress During COVID-19 Social Distancing. Front Psychol, 11, 563876. https://doi.org/10.3389/fpsyg.2020.563876 | Wrong population |
| 40 | Fong, Z. H., Tan, S. H., Mahendran, R., Kua, E. H., & Chee, T. T. (2021). Arts-based interventions to improve cognition in older persons with mild cognitive impairment: A systematic review of randomized controlled trials. Aging & Mental Health, 25(9), 1605-1617. https://doi.org/10.1080/13607863.2020.1786802 | Wrong outcome |
| 41 | Fredericks, S., Lapum, J., & Lo, J. (2012). Anxiety, depression, and self-management: a systematic review. Clin Nurs Res, 21(4), 411-430. https://doi.org/10.1177/1054773812436681 | Wrong population |
| 42 | García-Perdomo, H. A., Montealegre Cardona, L. M., Cordoba-Wagner, M. J., & Zapata-Copete, J. A. (2018). Music to reduce pain and anxiety in cystoscopy: a systematic review and meta-analysis. Journal of complementary & integrative medicine, 16(3). https://doi.org/10.1515/jcim-2018-0095 | Wrong population |
| 43 | Garza-Villarreal, E. A., Pando, V., Vuust, P., & Parsons, C. (2017). Music-Induced Analgesia in Chronic Pain Conditions: A Systematic Review and Meta-Analysis. Pain Physician, 20(7), 597-610. https://www.ncbi.nlm.nih.gov/pubmed/29149141 | Wrong population |
| 44 | Gaviola, M. A., Inder, K. J., Dilworth, S., Holliday, E. G., & Higgins, I. (2020). Impact of individualised music listening intervention on persons with dementia: A systematic review of randomised controlled trials. Australas J Ageing, 39(1), 10-20. https://doi.org/10.1111/ajag.12642 | Wrong outcome |
| 45 | Geretsegger, M., Elefant, C., Mossler, K. A., & Gold, C. (2014). Music therapy for people with autism spectrum disorder. Cochrane Database Syst Rev, 2014(6), CD004381. https://doi.org/10.1002/14651858.CD004381.pub3 | Update available |
| 46 | Gold, C., Heldal, T. O., Dahle, T., & Wigram, T. (2005). Music therapy for schizophrenia or schizophrenia-like illnesses. Cochrane Database Syst Rev(2), CD004025. https://doi.org/10.1002/14651858.CD004025.pub2 | Update available |
| 47 | Gold, C., Wigram, T., & Elefant, C. (2006). Music therapy for autistic spectrum disorder. Cochrane Database Syst Rev(2), CD004381. https://doi.org/10.1002/14651858.CD004381.pub2 | Update available |
| 48 | Gomez-Romero, M., Jimenez-Palomares, M., Rodriguez-Mansilla, J., Flores-Nieto, A., Garrido-Ardila, E. M., & Gonzalez Lopez-Arza, M. V. (2017). Benefits of music therapy on behaviour disorders in subjects diagnosed with dementia: a systematic review. Neurologia, 32(4), 253-263. https://doi.org/10.1016/j.nrl.2014.11.001 (Beneficios de la musicoterapia en las alteraciones conductuales de la demencia. Revision sistematica.) | Wrong outcome |
| 49 | Goris, E. D., Ansel, K. N., & Schutte, D. L. (2016). Quantitative systematic review of the effects of non-pharmacological interventions on reducing apathy in persons with dementia. J Adv Nurs, 72(11), 2612-2628. https://doi.org/10.1111/jan.13026 | Wrong outcome |
| 50 | Gurbuz-Dogan, R. N., Ali, A., Candy, B., & King, M. (2021). The effectiveness of Sufi music for mental health outcomes. A systematic review and meta-analysis of 21 randomised trials. Complement Ther Med, 57, 102664. https://doi.org/10.1016/j.ctim.2021.102664 | Wrong population |
| 51 | Hackett, K., Sabat, S. R., & Giovannetti, T. (2021). A person-centered framework for designing music-based therapeutic studies in dementia: current barriers and a path forward. Aging Ment Health, 1-10. https://doi.org/10.1080/13607863.2021.1931029 | Wrong outcome |
| 52 | Haslam, R., Heiderscheit, A., & Himmerich, H. (2022). A Systematic Review of Scientific Studies on the Effects of Music in People with Personality Disorders. International journal of environmental research and public health, 19(23). https://doi.org/10.3390/ijerph192315434 | Wrong outcome |
| 53 | Helgason, C., & Sarris, J. (2013). Mind-body medicine for schizophrenia and psychotic disorders: a review of the evidence. Clin Schizophr Relat Psychoses, 7(3), 138-148. https://doi.org/10.3371/CSRP.HESA.020813 | Wrong intervention |
| 54 | Hermans, D. G., Htay, U. H., & McShane, R. (2007). Non-pharmacological interventions for wandering of people with dementia in the domestic setting. Cochrane Database Syst Rev(1), CD005994. https://doi.org/10.1002/14651858.CD005994.pub2 | Wrong outcome |
| 55 | Hofbauer, L. M., Ross, S. D., & Rodriguez, F. S. (2022). Music-based interventions for community-dwelling people with dementia: A systematic review. Health & social care in the community, 30(6), 2186-2201. https://doi.org/10.1111/hsc.13895 | Wrong outcome |
| 56 | Hohmann, L., Bradt, J., Stegemann, T., & Koelsch, S. (2017). Effects of music therapy and music-based interventions in the treatment of substance use disorders: A systematic review. PLoS One, 12(11), e0187363. https://www.ncbi.nlm.nih.gov/pmc/articles/PMC5687713/pdf/pone.0187363.pdf | Wrong outcome |
| 57 | Hulme, C., Wright, J., Crocker, T., Oluboyede, Y., & House, A. (2010). Non-pharmacological approaches for dementia that informal carers might try or access: a systematic review. Int J Geriatr Psychiatry, 25(7), 756-763. https://onlinelibrary.wiley.com/doi/10.1002/gps.2429 | Wrong outcome |
| 58 | Iizuka, A., Suzuki, H., Ogawa, S., Kobayashi-Cuya, K. E., Kobayashi, M., Takebayashi, T., & Fujiwara, Y. (2019). Can cognitive leisure activity prevent cognitive decline in older adults? A systematic review of intervention studies. Geriatr Gerontol Int, 19(6), 469-482. https://onlinelibrary.wiley.com/doi/10.1111/ggi.13671 | Wrong intervention |
| 59 | Ing-Randolph, A. R., Phillips, L. R., & Williams, A. B. (2015). Group music interventions for dementia-associated anxiety: A systematic review. Int J Nurs Stud, 52(11), 1775-1784. https://doi.org/10.1016/j.ijnurstu.2015.06.014 | Wrong outcome |
| 60 | Jackson, J., Currie, K., Graham, C., & Robb, Y. (2011). The effectiveness of interventions to reduce undernutrition and promote eating in older adults with dementia: A systematic review. JBI Libr Syst Rev, 9(37), 1509-1550. https://doi.org/10.11124/01938924-201109370-00001 | Wrong outcome |
| 61 | Janus, S. I. M., Kosters, J., van den Bosch, K. A., Andringa, T. C., Zuidema, S. U., & Luijendijk, H. J. (2021). Sounds in nursing homes and their effect on health in dementia: a systematic review. Int Psychogeriatr, 33(6), 627-644. https://www.cambridge.org/core/journals/international-psychogeriatrics/article/abs/sounds-in-nursing-homes-and-their-effect-on-health-in-dementia-a-systematic-review/B715F0047E8CABF7CC799D14BDE276C0 | Wrong outcome |
| 62 | Jensen, L., & Padilla, R. (2017). Effectiveness of Environment-Based Interventions That Address Behavior, Perception, and Falls in People With Alzheimer's Disease and Related Major Neurocognitive Disorders: A Systematic Review. Am J Occup Ther, 71(5), 7105180030p7105180031-7105180030p7105180010. | Wrong outcome |
| 63 | Jespersen, K. V., Koenig, J., Jennum, P., & Vuust, P. (2015). Music for insomnia in adults. Cochrane Database Syst Rev(8), CD010459. https://doi.org/10.1002/14651858.CD010459.pub2 | Update available |
| 64 | Jiménez-Palomares, M., Rodríguez-Mansilla, J., González-López-Arza, M. V., Rodríguez-Domínguez, M. T., & Prieto-Tato, M. (2013). [Benefits of music therapy as therapy no pharmacology and rehabilitation moderate dementia]. Rev Esp Geriatr Gerontol, 48(5), 238-242. | Wrong outcome |
| 65 | Kim, E. Y., Hwang, S. D., & Kim, E. J. (2016). [Stimulation-Oriented Interventions for Behavioral Problems among People with Dementia: A Systematic Review and Meta-Analysis]. J Korean Acad Nurs, 46(4), 475-489. https://www.jkan.or.kr/Synapse/Data/PDFData/0006JKAN/jkan-46-475.pdf | Language |
| 66 | Kim, J., & Stegemann, T. (2016). Music listening for children and adolescents in health care contexts: A systematic review. The Arts in Psychotherapy, 51, 72-85. http://search.ebscohost.com/login.aspx?direct=true&db=psyh&AN=2016-55939-011&site=ehost-live | Wrong outcome |
| 67 | Kishita, N., Backhouse, T., & Mioshi, E. (2020). Nonpharmacological Interventions to Improve Depression, Anxiety, and Quality of Life (QoL) in People With Dementia: An Overview of Systematic Reviews. J Geriatr Psychiatry Neurol, 33(1), 28-41. https://doi.org/10.1177/0891988719856690 | Wrong publication |
| 68 | Klainin-Yobas, P., Oo, W. N., Suzanne Yew, P. Y., & Lau, Y. (2015). Effects of relaxation interventions on depression and anxiety among older adults: a systematic review. Aging Ment Health, 19(12), 1043-1055. https://www.tandfonline.com/doi/full/10.1080/13607863.2014.997191 | Wrong population |
| 69 | Koger, S. M., & Brotons, M. (2000). Music therapy for dementia symptoms. Cochrane Database Syst Rev(3), Cd001121. | Wrong outcome |
| 70 | Konno, R., Kang, H. S., & Makimoto, K. (2014). A best-evidence review of intervention studies for minimizing resistance-to-care behaviours for older adults with dementia in nursing homes. J Adv Nurs, 70(10), 2167-2180. https://doi.org/10.1111/jan.12432 | Wrong outcome |
| 71 | Korczak, D., Wastian, M., & Schneider, M. (2012). Therapy of the burnout syndrome. GMS Health Technol Assess, 8, Doc05. https://doi.org/10.3205/hta000103 | Wrong population |
| 72 | Kverno, K. S., Black, B. S., Nolan, M. T., & Rabins, P. V. (2009). Research on treating neuropsychiatric symptoms of advanced dementia with non-pharmacological strategies, 1998-2008: a systematic literature review. Int Psychogeriatr, 21(5), 825-843. https://doi.org/10.1017/S1041610209990196 | Wrong outcome |
| 73 | Lang, R., White, P. J., Machalicek, W., Rispoli, M., Kang, S., Aquilar, J., O'Reilly, M., Sigafoos, J., Lancioni, G., & Didden, R. (2009). Treatment of bruxism in individuals with developmental disabilities: a systematic review. Res Dev Disabil, 30(5), 809-818. https://doi.org/10.1016/j.ridd.2008.12.006 | Wrong population |
| 74 | Letrondo, P. A., Ashley, S. A., Flinn, A., Burton, A., Kador, T., & Mukadam, N. (2023). Systematic review of arts and culture-based interventions for people living with dementia and their caregivers. Ageing research reviews, 83, 101793. https://doi.org/10.1016/j.arr.2022.101793 | Wrong outcome |
| 75 | Leubner, D., & Hinterberger, T. (2017). Reviewing the Effectiveness of Music Interventions in Treating Depression. Front Psychol, 8, 1109. https://doi.org/10.3389/fpsyg.2017.01109 | Wrong outcome |
| 76 | Li, K., Cui, C., Zhang, H., Jia, L., Li, R., & Hu, H.-Y. (2022). Exploration of combined physical activity and music for patients with Alzheimer’s disease: A systematic review. Frontiers in Aging Neuroscience, 14. https://doi.org/10.3389/fnagi.2022.962475 | Wrong outcome |
| 77 | Li, X., Li, C., Hu, N., & Wang, T. (2020). Music Interventions for Disorders of Consciousness: A Systematic Review and Meta-analysis. J Neurosci Nurs, 52(4), 146-151. https://doi.org/10.1097/JNN.0000000000000511 | Wrong population |
| 78 | Li, Y. H., Chen, S. M., Chou, M. C., & Huang, T. Y. (2014). [The use of music intervention in nursing practice for elderly dementia patients: a systematic review]. Hu Li Za Zhi, 61(2), 84-94. | Wrong outcome |
| 79 | Livingston, G., Johnston, K., Katona, C., Paton, J., Lyketsos, C. G., & Old Age Task Force of the World Federation of Biological, P. (2005). Systematic review of psychological approaches to the management of neuropsychiatric symptoms of dementia. Am J Psychiatry, 162(11), 1996-2021. https://doi.org/10.1176/appi.ajp.162.11.1996 | Wrong outcome |
| 80 | Livingston, G., Kelly, L., Lewis-Holmes, E., Baio, G., Morris, S., Patel, N., Omar, R. Z., Katona, C., & Cooper, C. (2014a). Non-pharmacological interventions for agitation in dementia: systematic review of randomised controlled trials. Br J Psychiatry, 205(6), 436-442. https://doi.org/10.1192/bjp.bp.113.141119 | Wrong outcome |
| 81 | Livingston, G., Kelly, L., Lewis-Holmes, E., Baio, G., Morris, S., Patel, N., Omar, R. Z., Katona, C., & Cooper, C. (2014b). A systematic review of the clinical effectiveness and cost-effectiveness of sensory, psychological and behavioural interventions for managing agitation in older adults with dementia. Health Technol Assess, 18(39), 1-226, v-vi. https://doi.org/10.3310/hta18390 | Wrong outcome |
| 82 | Lourida, I., Gwernan-Jones, R., Abbott, R., Rogers, M., Green, C., Ball, S., Hemsley, A., Cheeseman, D., Clare, L., Moore, D., Hussey, C., Coxon, G., Llewellyn, D. J., Naldrett, T., & Thompson Coon, J. (2020). Activity interventions to improve the experience of care in hospital for people living with dementia: a systematic review. BMC Geriatr, 20(1), 131. https://doi.org/10.1186/s12877-020-01534-7 | Wrong outcome |
| 83 | Lu, G., Jia, R., Liang, D., Yu, J., Wu, Z., & Chen, C. (2021). Effects of music therapy on anxiety: A meta-analysis of randomized controlled trials. Psychiatry Research, 304. https://doi.org/10.1016/j.psychres.2021.114137 | Wrong population |
| 84 | Lyra, L., Rizzo, L. E., Sunahara, C. S., Pachito, D. V., Latorraca, C. O. C., Martimbianco, A. L. C., & Riera, R. (2017). What do Cochrane systematic reviews say about interventions for autism spectrum disorders? Sao Paulo Med J, 135(2), 192-201. https://www.scielo.br/j/spmj/a/ddmbY5KH44j8dZCbkCM9Skx/?lang=en&format=pdf | Wrong publication |
| 85 | Maratos, A. S., Gold, C., Wang, X., & Crawford, M. J. (2008). Music therapy for depression. Cochrane Database Syst Rev(1), CD004517. https://doi.org/10.1002/14651858.CD004517.pub2 | Update available |
| 86 | Mayer-Benarous, H., Benarous, X., Vonthron, F., & Cohen, D. (2021). Music Therapy for Children With Autistic Spectrum Disorder and/or Other Neurodevelopmental Disorders: A Systematic Review. Front Psychiatry, 12, 643234. https://www.ncbi.nlm.nih.gov/pmc/articles/PMC8062803/pdf/fpsyt-12-643234.pdf | Wrong outcome |
| 87 | Mays, K. L., Clark, D. L., & Gordon, A. J. (2008). Treating addiction with tunes: a systematic review of music therapy for the treatment of patients with addictions. Subst Abus, 29(4), 51-59. https://doi.org/10.1080/08897070802418485 | Wrong outcome |
| 88 | McDermott, O., Crellin, N., Ridder, H. M., & Orrell, M. (2013). Music therapy in dementia: a narrative synthesis systematic review. Int J Geriatr Psychiatry, 28(8), 781-794. https://doi.org/10.1002/gps.3895 | Wrong outcome |
| 89 | McFerran, K. S., Garrido, S., & Saarikallio, S. (2016). A critical interpretive synthesis of the literature linking music and adolescent mental health. Youth & Society, 48(4), 521-538. http://search.ebscohost.com/login.aspx?direct=true&db=psyh&AN=2016-25417-004&site=ehost-live | Wrong outcome |
| 90 | McKinney, C. H., & Honig, T. J. (2017). Health Outcomes of a Series of Bonny Method of Guided Imagery and Music Sessions: A Systematic Review. J Music Ther, 54(1), 1-34. https://doi.org/10.1093/jmt/thw016 | Wrong outcome |
| 91 | Megranahan, K., & Lynskey, M. T. (2018). Do creative arts therapies reduce substance misuse? A systematic review. The Arts in Psychotherapy, 57, 50-58. http://search.ebscohost.com/login.aspx?direct=true&db=psyh&AN=2018-14544-008&site=ehost-live | Wrong outcome |
| 92 | Melo, F. L., Mendoza, J. F. W., Latorraca, C. O. C., Pacheco, R. L., Martimbianco, A. L. C., Pachito, D. V., & Riera, R. (2018). What do Cochrane systematic reviews say about interventions for insomnia? Sao Paulo Med J, 136(6), 579-585. https://www.scielo.br/j/spmj/a/wpTz8F9T6DZ4pshkrLgRLXM/?lang=en&format=pdf | Wrong publication |
| 93 | Millan-Calenti, J. C., Lorenzo-Lopez, L., Alonso-Bua, B., de Labra, C., Gonzalez-Abraldes, I., & Maseda, A. (2016). Optimal nonpharmacological management of agitation in Alzheimer's disease: challenges and solutions. Clin Interv Aging, 11, 175-184. https://doi.org/10.2147/CIA.S69484 | Wrong outcome |
| 94 | Moola, S., Pearson, A., & Hagger, C. (2011). Effectiveness of music interventions on dental anxiety in paediatric and adult patients: a systematic review. JBI Libr Syst Rev, 9(18), 588-630. https://doi.org/10.11124/01938924-201109180-00001 | Wrong population |
| 95 | Moreira, S. V., Justi, F., & Moreira, M. (2018). Can musical intervention improve memory in Alzheimer's patients? Evidence from a systematic review. Dement Neuropsychol, 12(2), 133-142. https://www.ncbi.nlm.nih.gov/pmc/articles/PMC6022981/pdf/1980-5764-dn-12-02-0133.pdf | Wrong outcome |
| 96 | Morgan, A. J., & Jorm, A. F. (2008). Self-help interventions for depressive disorders and depressive symptoms: a systematic review. Ann Gen Psychiatry, 7, 13. https://doi.org/10.1186/1744-859X-7-13 | Wrong outcome |
| 97 | Morison, L., Simonds, L., & Stewart, S.-J. F. (2022). Effectiveness of creative arts-based interventions for treating children and adolescents exposed to traumatic events: A systematic review of the quantitative evidence and meta-analysis. Arts & Health: An International Journal for Research, Policy and Practice, 14(3), 237-262. https://doi.org/10.1080/17533015.2021.2009529 | Wrong intervention |
| 98 | Mossler, K., Chen, X., Heldal, T. O., & Gold, C. (2011). Music therapy for people with schizophrenia and schizophrenia-like disorders. Cochrane Database Syst Rev(12), CD004025. https://doi.org/10.1002/14651858.CD004025.pub3 | Update available |
| 99 | Moumdjian, L., Sarkamo, T., Leone, C., Leman, M., & Feys, P. (2017). Effectiveness of music-based interventions on motricity or cognitive functioning in neurological populations: a systematic review. Eur J Phys Rehabil Med, 53(3), 466-482. https://doi.org/10.23736/S1973-9087.16.04429-4 | Wrong population |
| 100 | Mu, P. F., Chen, Y. C., & Cheng, S. C. (2009). The effectiveness of non-pharmacological pain management in relieving chronic pain for children and adolescents. JBI Libr Syst Rev, 7(34), 1489-1543. https://doi.org/10.11124/01938924-200907340-00001 | Wrong population |
| 101 | Nilsson, U. (2008). The anxiety- and pain-reducing effects of music interventions: a systematic review. Aorn j, 87(4), 780-807. https://doi.org/10.1016/j.aorn.2007.09.013 | Wrong population |
| 102 | Noone, D., Stott, J., Aguirre, E., Llanfear, K., & Spector, A. (2019). Meta-analysis of psychosocial interventions for people with dementia and anxiety or depression. Aging Ment Health, 23(10), 1282-1291. https://doi.org/10.1080/13607863.2018.1495177 | Wrong outcome |
| 103 | Nordhausen, T., Langner, H., Fleischer, S., Meyer, G., & Berg, A. (2019). [Improving psychosocial health of nursing home residents: a systematic review of interventions for prevention and health promotion]. Z Evid Fortbild Qual Gesundhwes, 147-148, 7-19. https://doi.org/10.1016/j.zefq.2019.09.005 (Starkung der psychosozialen Gesundheit von Bewohnerinnen und Bewohnern der stationaren Langzeitpflege: Systematische Ubersicht zu Interventionen der Pravention und Gesundheitsforderung.) | Wrong outcome |
| 104 | O'Connor, D. W., Ames, D., Gardner, B., & King, M. (2009a). Psychosocial treatments of behavior symptoms in dementia: a systematic review of reports meeting quality standards. Int Psychogeriatr, 21(2), 225-240. https://doi.org/10.1017/S1041610208007588 | Wrong outcome |
| 105 | O'Connor, D. W., Ames, D., Gardner, B., & King, M. (2009b). Psychosocial treatments of psychological symptoms in dementia: a systematic review of reports meeting quality standards. Int Psychogeriatr, 21(2), 241-251. https://doi.org/10.1017/S1041610208008223 | Wrong outcome |
| 106 | Olley, R., & Morales, A. (2018). Systematic review of evidence underpinning non-pharmacological therapies in dementia. Aust Health Rev, 42(4), 361-369. https://doi.org/10.1071/AH16212 | Wrong outcome |
| 107 | Opie, J., Rosewarne, R., & O'Connor, D. W. (1999). The efficacy of psychosocial approaches to behaviour disorders in dementia: a systematic literature review. Aust N Z J Psychiatry, 33(6), 789-799. https://doi.org/10.1046/j.1440-1614.1999.00652.x | Wrong outcome |
| 108 | Orgeta, V., McDonald, K. R., Poliakoff, E., Hindle, J. V., Clare, L., & Leroi, I. (2020). Cognitive training interventions for dementia and mild cognitive impairment in Parkinson's disease. Cochrane Database Syst Rev, 2(2), CD011961. https://doi.org/10.1002/14651858.CD011961.pub2 | Wrong intervention |
| 109 | Panteleeva, Y., Ceschi, G., Glowinski, D., Courvoisier, D. S., & Grandjean, D. (2018). Music for anxiety? Meta-analysis of anxiety reduction in non-clinical samples. Psychology of Music, 46(4), 473-487. http://search.ebscohost.com/login.aspx?direct=true&db=psyh&AN=2018-34095-002&site=ehost-live | Wrong population |
| 110 | Parslow, R., Morgan, A. J., Allen, N. B., Jorm, A. F., O'Donnell, C. P., & Purcell, R. (2008). Effectiveness of complementary and self-help treatments for anxiety in children and adolescents. Med J Aust, 188(6), 355-359. https://doi.org/10.5694/j.1326-5377.2008.tb01654.x | Wrong outcome |
| 111 | Pestana-Santos, M., Pires, R., Goncalves, A., Parola, V., Santos, M. R., & Lomba, L. (2021). Nonpharmacological interventions used in the perioperative period to prevent anxiety in adolescents: a scoping review. JBI Evid Synth. | Wrong population |
| 112 | Petrovsky, D. V., Ramesh, P., McPhillips, M. V., & Hodgson, N. A. (2021). Effects of music interventions on sleep in older adults: A systematic review. Geriatr Nurs, 42(4), 869-879. https://doi.org/10.1016/j.gerinurse.2021.04.014 | Wrong population |
| 113 | Reschke-Hernandez, A. E. (2011). History of music therapy treatment interventions for children with autism. J Music Ther, 48(2), 169-207. https://doi.org/10.1093/jmt/48.2.169 | Wrong outcome |
| 114 | Rieckmann, N., Schwarzbach, C., Nocon, M., Roll, S., Vauth, C., Willich, S. N., & Greiner, W. (2009). Concepts of care for people with dementia. GMS Health Technol Assess, 5, Doc01. https://doi.org/10.3205/hta000063 | Wrong intervention |
| 115 | Robinson, L., Hutchings, D., Corner, L., Beyer, F., Dickinson, H., Vanoli, A., Finch, T., Hughes, J., Ballard, C., May, C., & Bond, J. (2006). A systematic literature review of the effectiveness of non-pharmacological interventions to prevent wandering in dementia and evaluation of the ethical implications and acceptability of their use. Health Technol Assess, 10(26), iii, ix-108. https://www.journalslibrary.nihr.ac.uk/hta/hta10260/ | Wrong outcome |
| 116 | Robinson, L., Hutchings, D., Dickinson, H. O., Corner, L., Beyer, F., Finch, T., Hughes, J., Vanoli, A., Ballard, C., & Bond, J. (2007). Effectiveness and acceptability of non-pharmacological interventions to reduce wandering in dementia: a systematic review. Int J Geriatr Psychiatry, 22(1), 9-22. https://doi.org/10.1002/gps.1643 | Wrong outcome |
| 117 | Ronzi, S., Orton, L., Pope, D., Valtorta, N. K., & Bruce, N. G. (2018). What is the impact on health and wellbeing of interventions that foster respect and social inclusion in community-residing older adults? A systematic review of quantitative and qualitative studies. Syst Rev, 7(1), 26. https://www.ncbi.nlm.nih.gov/pmc/articles/PMC5789687/pdf/13643_2018_Article_680.pdf | Wrong outcome |
| 118 | Rossignol, D. A. (2009). Novel and emerging treatments for autism spectrum disorders: a systematic review. Ann Clin Psychiatry, 21(4), 213-236. https://www.ncbi.nlm.nih.gov/pubmed/19917212 | Wrong outcome |
| 119 | Schmid, W., & Ostermann, T. (2010). Home-based music therapy--a systematic overview of settings and conditions for an innovative service in healthcare. BMC Health Serv Res, 10, 291. https://doi.org/10.1186/1472-6963-10-291 | Wrong outcome |
| 120 | Schmitt, B., & Frolich, L. (2007). [Creative therapy options for patients with dementia--a systematic review]. Fortschr Neurol Psychiatr, 75(12), 699-707. https://doi.org/10.1055/s-2006-944298 (Kreative Therapieansatze in der Behandlung von Demenzen--eine systematische Ubersicht.) | Wrong outcome |
| 121 | Seitz, D. P., Brisbin, S., Herrmann, N., Rapoport, M. J., Wilson, K., Gill, S. S., Rines, J., Le Clair, K., & Conn, D. (2012). Efficacy and feasibility of nonpharmacological interventions for neuropsychiatric symptoms of dementia in long term care: a systematic review. J Am Med Dir Assoc, 13(6), 503-506 e502. https://doi.org/10.1016/j.jamda.2011.12.059 | Wrong outcome |
| 122 | Sharew, N. T. (2022). The Effect of Multimodal Non-pharmacological Interventions on Cognitive Function Improvement for People With Dementia: A Systematic Review. Frontiers in public health, 10, 894930. https://doi.org/10.3389/fpubh.2022.894930 | Wrong intervention |
| 123 | Siders, C., Nelson, A., Brown, L. M., Joseph, I., Algase, D., Beattie, E., & Verbosky-Cadena, S. (2004). Evidence for implementing nonpharmacological interventions for wandering. Rehabil Nurs, 29(6), 195-206. https://www.ncbi.nlm.nih.gov/pubmed/15597998 | Wrong outcome |
| 124 | Sihvonen, A. J., Leo, V., Sarkamo, T., & Soinila, S. (2014). [Effectiveness of music in brain rehabilitation. A systematic review]. Duodecim, 130(18), 1852-1860. https://www.ncbi.nlm.nih.gov/pubmed/25558627 (Musiikin vaikuttavuus aivojen kuntoutuksessa.) | Wrong outcome |
| 125 | Silverman, M. J. (2008). Quantitative comparison of cognitive behavioral therapy and music therapy research: a methodological best-practices analysis to guide future investigation for adult psychiatric patients. J Music Ther, 45(4), 457-506. https://doi.org/10.1093/jmt/45.4.457 | Wrong outcome |
| 126 | Sinha, Y., Silove, N., Hayen, A., & Williams, K. (2011). Auditory integration training and other sound therapies for autism spectrum disorders (ASD). Cochrane Database Syst Rev, 2011(12), CD003681. https://doi.org/10.1002/14651858.CD003681.pub3 | Wrong intervention |
| 127 | Sinha, Y., Silove, N., Wheeler, D., & Williams, K. (2004). Auditory integration training and other sound therapies for autism spectrum disorders. Cochrane Database Syst Rev(1), CD003681. https://doi.org/10.1002/14651858.CD003681.pub2 | Wrong intervention |
| 128 | Snowden, M., Sato, K., & Roy-Byrne, P. (2003). Assessment and treatment of nursing home residents with depression or behavioral symptoms associated with dementia: a review of the literature. J Am Geriatr Soc, 51(9), 1305-1317. https://doi.org/10.1046/j.1532-5415.2003.51417.x | Wrong outcome |
| 129 | Sousa, L., Dowson, B., McDermott, O., Schneider, J., & Fernandes, L. (2020). Music-based interventions in the acute setting for patients with dementia: a systematic review. Eur Geriatr Med, 11(6), 929-943. https://doi.org/10.1007/s41999-020-00381-4 | Wrong outcome |
| 130 | Spelten, E., Thomas, B., O'Meara, P. F., Maguire, B. J., FitzGerald, D., & Begg, S. J. (2020). Organisational interventions for preventing and minimising aggression directed towards healthcare workers by patients and patient advocates. Cochrane Database Syst Rev, 4(4), CD012662. https://doi.org/10.1002/14651858.CD012662.pub2 | Wrong intervention |
| 131 | Stern, C. (2013). Music interventions for preoperative anxiety. International Journal of Evidence-Based Healthcare, 11(3), 208-209. http://search.ebscohost.com/login.aspx?direct=true&db=psyh&AN=2013-31576-010&site=ehost-live | Wrong population |
| 132 | Su Maw, S., & Haga, C. (2018). Effectiveness of cognitive, developmental, and behavioural interventions for Autism Spectrum Disorder in preschool-aged children: A systematic review and meta-analysis. Heliyon, 4(9), e00763. https://doi.org/10.1016/j.heliyon.2018.e00763 | Wrong Outcome |
| 133 | Swedish Council on Health Technology, A. (2008). SBU Systematic Reviews. Swedish Council on Health Technology Assessment (SBU) Copyright © 2008 by the Swedish Council on Health Technology Assessment. | Wrong publication |
| 134 | Tan, D. G. H., Boo, B. M. B., Chong, C. S., Tan, M. M. L.-L., & Wong, B.-S. (2022). Effectiveness of home-based, non-exercise interventions for dementia: A systematic review. Frontiers in Aging Neuroscience, 14. https://doi.org/10.3389/fnagi.2022.846271 | Wrong outcome |
| 135 | Testa, F., Arunachalam, S., Heiderscheit, A., & Himmerich, H. (2020). A Systematic Review of Scientific Studies on the Effects of Music in People with or at Risk for Eating Disorders. Psychiatr Danub, 32(3), 334-345. | Wrong population |
| 136 | Thekkumkara, S. N., Jagannathan, A., Muliyala, K. P., & Murthy, P. (2022). Psychosocial Interventions for Prisoners with Mental and Substance Use Disorders: A Systematic Review. Indian journal of psychological medicine, 44(3), 211-217. https://doi.org/10.1177/02537176211061655 | Wrong outcome |
| 137 | Thrane, S. E., Hsieh, K., Donahue, P., Tan, A., Exline, M. C., & Balas, M. C. (2019). Could complementary health approaches improve the symptom experience and outcomes of critically ill adults? A systematic review of randomized controlled trials. Complement Ther Med, 47, 102166. | Wrong population |
| 138 | Travers, C., Brooks, D., Hines, S., O'Reilly, M., McMaster, M., He, W., MacAndrew, M., Fielding, E., Karlsson, L., & Beattie, E. (2016). Effectiveness of meaningful occupation interventions for people living with dementia in residential aged care: a systematic review. JBI Database System Rev Implement Rep, 14(12), 163-225. https://doi.org/10.11124/JBISRIR-2016-003230 | Wrong intervention |
| 139 | Treurnicht Naylor, K., Kingsnorth, S., Lamont, A., McKeever, P., & Macarthur, C. (2011). The effectiveness of music in pediatric healthcare: a systematic review of randomized controlled trials. Evid Based Complement Alternat Med, 2011, 464759. https://doi.org/10.1155/2011/464759 | Wrong outcome |
| 140 | Truong, T. P. A., Applewhite, B., Heiderscheit, A., & Himmerich, H. (2021). A Systematic Review of Scientific Studies and Case Reports on Music and Obsessive-Compulsive Disorder. International journal of environmental research and public health, 18(22). https://doi.org/10.3390/ijerph182211799 | Wrong outcome |
| 141 | Tungpunkom, P., Maayan, N., & Soares-Weiser, K. (2012). Life skills programmes for chronic mental illnesses. Cochrane Database Syst Rev, 1(1), CD000381. https://doi.org/10.1002/14651858.CD000381.pub3 | Wrong intervention |
| 142 | van der Steen, J. T., van Soest-Poortvliet, M. C., van der Wouden, J. C., Bruinsma, M. S., Scholten, R. J., & Vink, A. C. (2017). Music-based therapeutic interventions for people with dementia. Cochrane Database Syst Rev, 5(5), CD003477. https://doi.org/10.1002/14651858.CD003477.pub3 | Update available |
| 143 | Van't Hooft, J. J., Pijnenburg, Y. A. L., Sikkes, S. A. M., Scheltens, P., Spikman, J. M., Jaschke, A. C., Warren, J. D., & Tijms, B. M. (2021). Frontotemporal dementia, music perception and social cognition share neurobiological circuits: A meta-analysis. Brain Cogn, 148, 105660. https://doi.org/10.1016/j.bandc.2020.105660 | Wrong intervention |
| 144 | Veerman, S. R. T., Schulte, P. F. J., & de Haan, L. (2017). Treatment for Negative Symptoms in Schizophrenia: A Comprehensive Review. Drugs, 77(13), 1423-1459. https://doi.org/10.1007/s40265-017-0789-y | Wrong outcome |
| 145 | Vink, A. C., Birks, J. S., Bruinsma, M. S., & Scholten, R. J. (2004). Music therapy for people with dementia. Cochrane Database Syst Rev(3), CD003477. https://doi.org/10.1002/14651858.CD003477.pub2 | Wrong outcome |
| 146 | Wade, D. F., Moon, Z., Windgassen, S. S., Harrison, A. M., Morris, L., & Weinman, J. A. (2016). Non-pharmacological interventions to reduce ICU-related psychological distress: a systematic review. Minerva Anestesiol, 82(4), 465-478. https://www.ncbi.nlm.nih.gov/pubmed/26505225 | Wrong population |
| 147 | Wang, L. Y., Pei, J., Zhan, Y. J., & Cai, Y. W. (2020). Overview of Meta-Analyses of Five Non-pharmacological Interventions for Alzheimer's Disease. Front Aging Neurosci, 12, 594432. https://doi.org/10.3389/fnagi.2020.594432 | Wrong publication |
| 148 | Wang, X.-S., Li, J.-J., Wang, Y.-S., Yu, C.-C., He, C., Huang, Z.-S., Jiang, T., Hao, Q., & Kong, L.-H. (2021). Acupuncture and Related Therapies for the Cognitive Function of Alzheimer's Disease: A Network Meta-Analysis. Iranian journal of public health, 50(12), 2411-2426. https://doi.org/10.18502/ijph.v50i12.7924 | Wrong intervention |
| 149 | Watson, R., & Green, S. M. (2006). Feeding and dementia: a systematic literature review. J Adv Nurs, 54(1), 86-93. https://doi.org/10.1111/j.1365-2648.2006.03793.x | Wrong outcome |
| 150 | Weitlauf, A. S., Sathe, N., McPheeters, M. L., & Warren, Z. E. (2017). Interventions Targeting Sensory Challenges in Autism Spectrum Disorder: A Systematic Review. Pediatrics, 139(6). https://doi.org/10.1542/peds.2017-0347 | Wrong outcome |
| 151 | Whear, R., Abbott, R., Thompson-Coon, J., Bethel, A., Rogers, M., Hemsley, A., Stahl-Timmins, W., & Stein, K. (2014). Effectiveness of mealtime interventions on behavior symptoms of people with dementia living in care homes: a systematic review. J Am Med Dir Assoc, 15(3), 185-193. https://doi.org/10.1016/j.jamda.2013.10.016 | Wrong outcome |
| 152 | Wigram, T., & Gold, C. (2006). Music therapy in the assessment and treatment of autistic spectrum disorder: clinical application and research evidence. Child Care Health Dev, 32(5), 535-542. https://doi.org/10.1111/j.1365-2214.2006.00615.x | Wrong outcome |
| 153 | Witusik, A., & Pietras, T. (2019). Music therapy as a complementary form of therapy for mental disorders. Pol Merkur Lekarski, 47(282), 240-243. | Wrong outcome |
| 154 | Woods, B., Aguirre, E., Spector, A. E., & Orrell, M. (2012). Cognitive stimulation to improve cognitive functioning in people with dementia. Cochrane Database Syst Rev(2), CD005562. https://doi.org/10.1002/14651858.CD005562.pub2 | Wrong intervention |
| 155 | Woods, B., O'Philbin, L., Farrell, E. M., Spector, A. E., & Orrell, M. (2018). Reminiscence therapy for dementia. Cochrane Database Syst Rev, 3, CD001120. https://doi.org/10.1002/14651858.CD001120.pub3 | Wrong intervention |
| 156 | Xu, B., Sui, Y., Zhu, C., Yang, X., Zhou, J., Li, L., Ren, L., & Wang, X. (2017). Music intervention on cognitive dysfunction in healthy older adults: a systematic review and meta-analysis. Neurol Sci, 38(6), 983-992. https://doi.org/10.1007/s10072-017-2878-9 | Wrong population |
| 157 | Ye, P., Huang, Z., Zhou, H., & Tang, Q. (2021). Music-based intervention to reduce aggressive behavior in children and adolescents: A meta-analysis. Medicine (Baltimore), 100(4), e23894. https://doi.org/10.1097/MD.0000000000023894 | Wrong population |
| 158 | Yoo, J., Oh, J., Kim, S.-Y., Shin, J., Kim, S., & Roh, C. (2022). Impact of Digital Device, Exercise, and Music Intervention Programs on the Cognition and Depression of the Elderly in South Korea: A Meta-Regression Analysis. International journal of environmental research and public health, 19(7). https://doi.org/10.3390/ijerph19074036 | Wrong outcome |
| 159 | You, Z. Y., & Wang, J. Z. (2002). [Meta-analysis of assisted music therapy for chronic schizophrenia]. Zhongguo Yi Xue Ke Xue Yuan Xue Bao, 24(6), 564-567. https://www.ncbi.nlm.nih.gov/pubmed/12905680 | Language |
| 160 | Yun-Springer, A. B., & Silverman, M. J. (2014). Levels of evidence in the journal of music therapy from 2000–2009: Descriptive analyses by year and clinical population. Music Therapy Perspectives, 32(2), 185-190. http://search.ebscohost.com/login.aspx?direct=true&db=psyh&AN=2014-57179-009&site=ehost-live | Wrong outcome |
| 161 | Zarafshan, H., Salmanian, M., Aghamohammadi, S., Mohammadi, M. R., & Mostafavi, S. A. (2017). Effectiveness of Non-Pharmacological Interventions on Stereotyped and Repetitive Behaviors of Pre-school Children With Autism: A Systematic Review. Basic Clin Neurosci, 8(2), 95-103. https://doi.org/10.18869/nirp.bcn.8.2.95 | Wrong outcome |
| 162 | Zhang, S., Liu, D., Ye, D., Li, H., & Chen, F. (2017). Can music-based movement therapy improve motor dysfunction in patients with Parkinson's disease? Systematic review and meta-analysis. Neurol Sci, 38(9), 1629-1636. https://link.springer.com/article/10.1007%2Fs10072-017-3020-8 | Wrong population |
| 163 | Zhao, K., Bai, Z. G., Bo, A., & Chi, I. (2016). A systematic review and meta-analysis of music therapy for the older adults with depression. Int J Geriatr Psychiatry, 31(11), 1188-1198. https://doi.org/10.1002/gps.4494 | Wrong population |
| 164 | Zhou, L. Y., Zhang, Y., Tian, Y., Fu, X., Wang, L. Z., & Xie, C. G. (2020). Effect of music intervention on mental health in patients with diabetes mellitus: protocol for a systematic review and meta-analysis of randomizsed controlled trials. BMJ Open, 10(8), e036268. https://doi.org/10.1136/bmjopen-2019-036268 | Wrong population |
| 165 | Zhu, Y., Wang, R., Tang, X., Li, Q., Xu, G., & Zhang, A. (2021). The effect of music, massage, yoga and exercise on antenatal depression: A meta-analysis. J Affect Disord, 292, 592-602. https://doi.org/10.1016/j.jad.2021.05.122 | Wrong population |
