# Supplementary material for: Evidence for music therapy and music medicine in psychiatry: transdiagnostic meta-review of meta-analyses
Source: BJPsych Open. 2024 Dec 13;11(1):e4. doi: 10.1192/bjo.2024.826 (PMC11733488; doi:10.1192/bjo.2024.826)

Study	Experimental Total	Experimental Mean	Experimental SD	Control Total	Control Mean	Control SD	SMD	95%−CI	Weight
Porter 2017	16	−71.19	33.4300	19	−89.55	30.8000	0.56	[−0.12; 1.24]	38.7%
Bieleninik 2017	155	−3.66	2.1100	135	−3.40	2.0500	−0.12	[−0.36; 0.11]	61.3%
**Random effects model**	**171**			**154**			**0.14**	**[−0.51; 0.79]**	**100.0%**

Heterogeneity: $I^2 = 71\%$, $\tau^2 = 0.1674$, $p = 0.06$

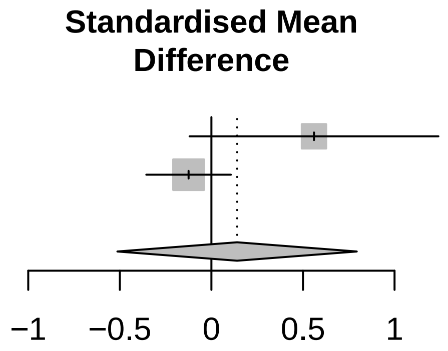

Supplement: Lassner et al. supplementary material 5 — Lassner et al. supplementary material [file S2056472424008263sup005.zip › analysis_2023.08.18/analysis/autism/autism_adaptive_behavior_followup.pdf]
