# Supplementary material for: Evidence for music therapy and music medicine in psychiatry: transdiagnostic meta-review of meta-analyses
Source: BJPsych Open. 2024 Dec 13;11(1):e4. doi: 10.1192/bjo.2024.826 (PMC11733488; doi:10.1192/bjo.2024.826)

Study	Experimental Total	Experimental Mean	Experimental SD	Control Total	Control Mean	Control SD	SMD	95%−CI	Weight
Moon 2010	10	2.51	1.0000	10	0.00	1.0000	2.40	[1.20; 3.61]	46.7%
Porter 2017	16	18.88	6.4300	19	16.11	6.1500	0.43	[−0.24; 1.10]	53.3%
**Random effects model**	**26**			**29**			**1.35**	**[−0.58; 3.28]**	**100.0%**

Heterogeneity: $I^2 = 87\%$, $\tau^2 = 1.6978$, $p < 0.01$

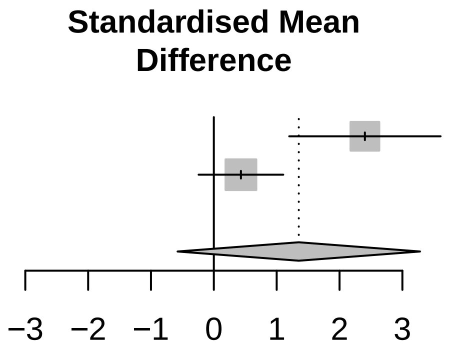

Supplement: Lassner et al. supplementary material 5 — Lassner et al. supplementary material [file S2056472424008263sup005.zip › analysis_2023.08.18/analysis/autism/autism_identify_formation_endpoint.pdf]
