# Supplementary material for: Evidence for music therapy and music medicine in psychiatry: transdiagnostic meta-review of meta-analyses
Source: BJPsych Open. 2024 Dec 13;11(1):e4. doi: 10.1192/bjo.2024.826 (PMC11733488; doi:10.1192/bjo.2024.826)

Study	Experimental Total	Experimental Mean	Experimental SD	Control Total	Control Mean	Control SD	Standardised Mean Difference	SMD	95%−CI	Weight
Amiri 2019	15	−2.37	4.8000	15	−4.80	1.3700		0.67	[−0.07; 1.41]	48.0%
Jespersen 2019	18	−8.70	3.8000	15	−11.20	3.0000		0.70	[−0.00; 1.41]	52.0%
**Random effects model**	**33**			**30**				**0.69**	**[0.18; 1.20]**	**100.0%**

Heterogeneity: $I^2 = 0\%$, $\tau^2 = 0$, $p = 0.95$

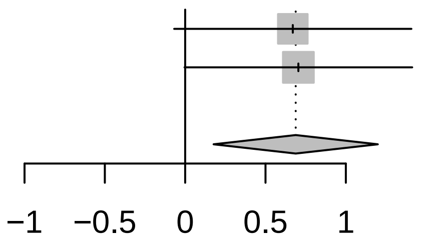

Supplement: Lassner et al. supplementary material 5 — Lassner et al. supplementary material [file S2056472424008263sup005.zip › analysis_2023.08.18/analysis/insomnia/primary_insomnia_sleep_qol_endpoint.pdf]
