# Supplementary figures and images for: Evidence for music therapy and music medicine in psychiatry: transdiagnostic meta-review of meta-analyses
Source: BJPsych Open. 2024 Dec 13;11(1):e4. doi: 10.1192/bjo.2024.826 (PMC11733488; doi:10.1192/bjo.2024.826)

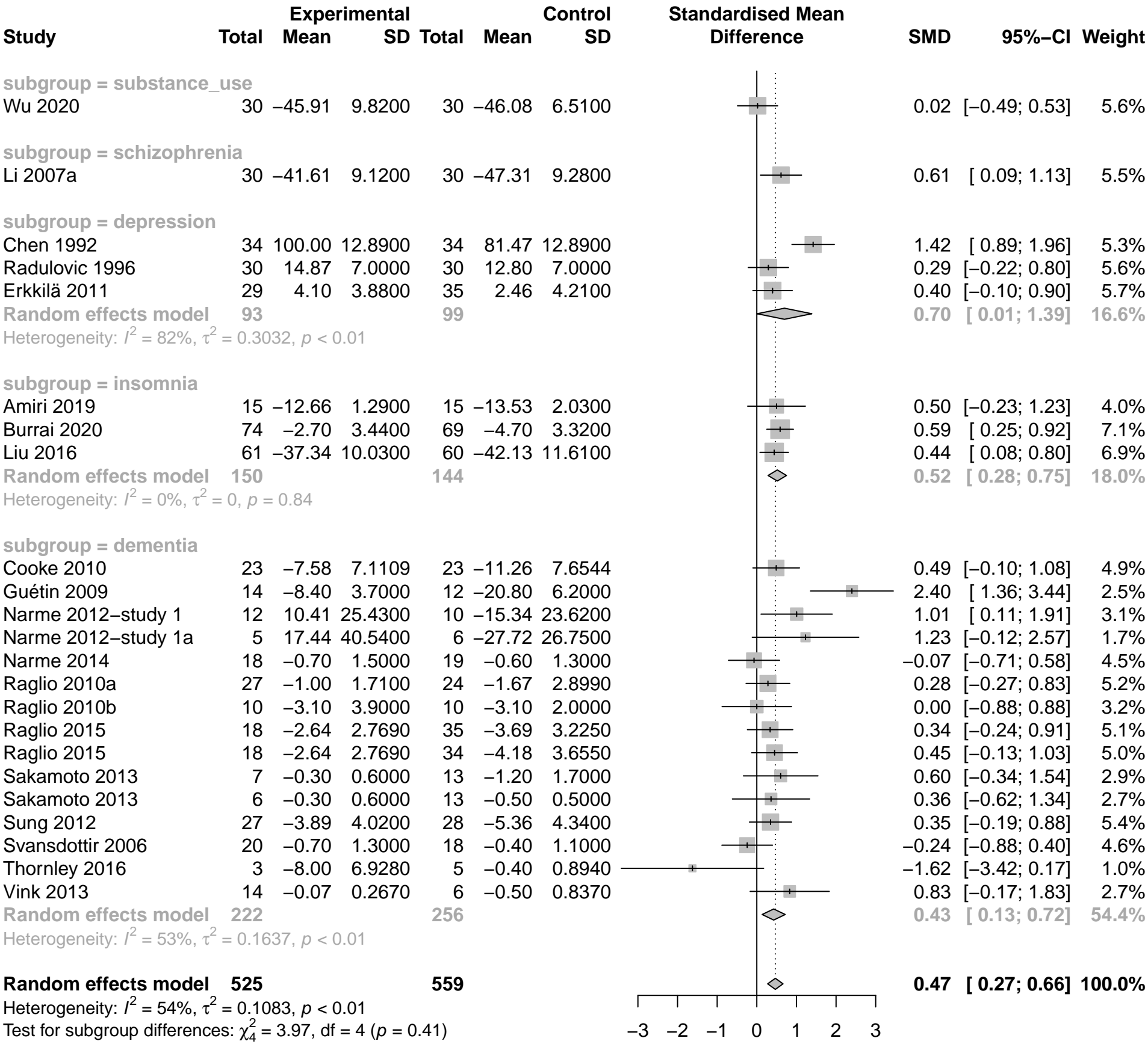

Supplement: Lassner et al. supplementary material 5 — Lassner et al. supplementary material [file S2056472424008263sup005.zip › analysis_2023.08.18/analysis/any anxiety/any_anxiety_endpoint.pdf]

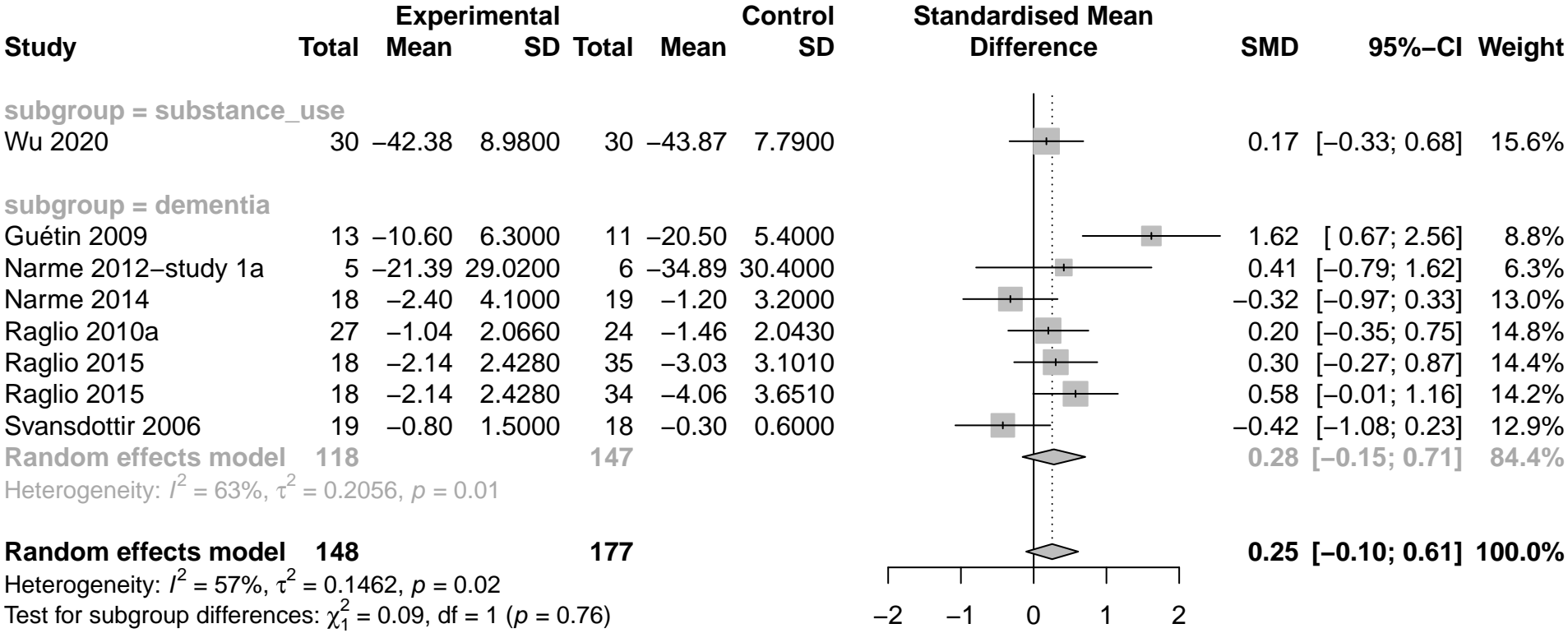

Supplement: Lassner et al. supplementary material 5 — Lassner et al. supplementary material [file S2056472424008263sup005.zip › analysis_2023.08.18/analysis/any anxiety/any_anxiety_followup.pdf]

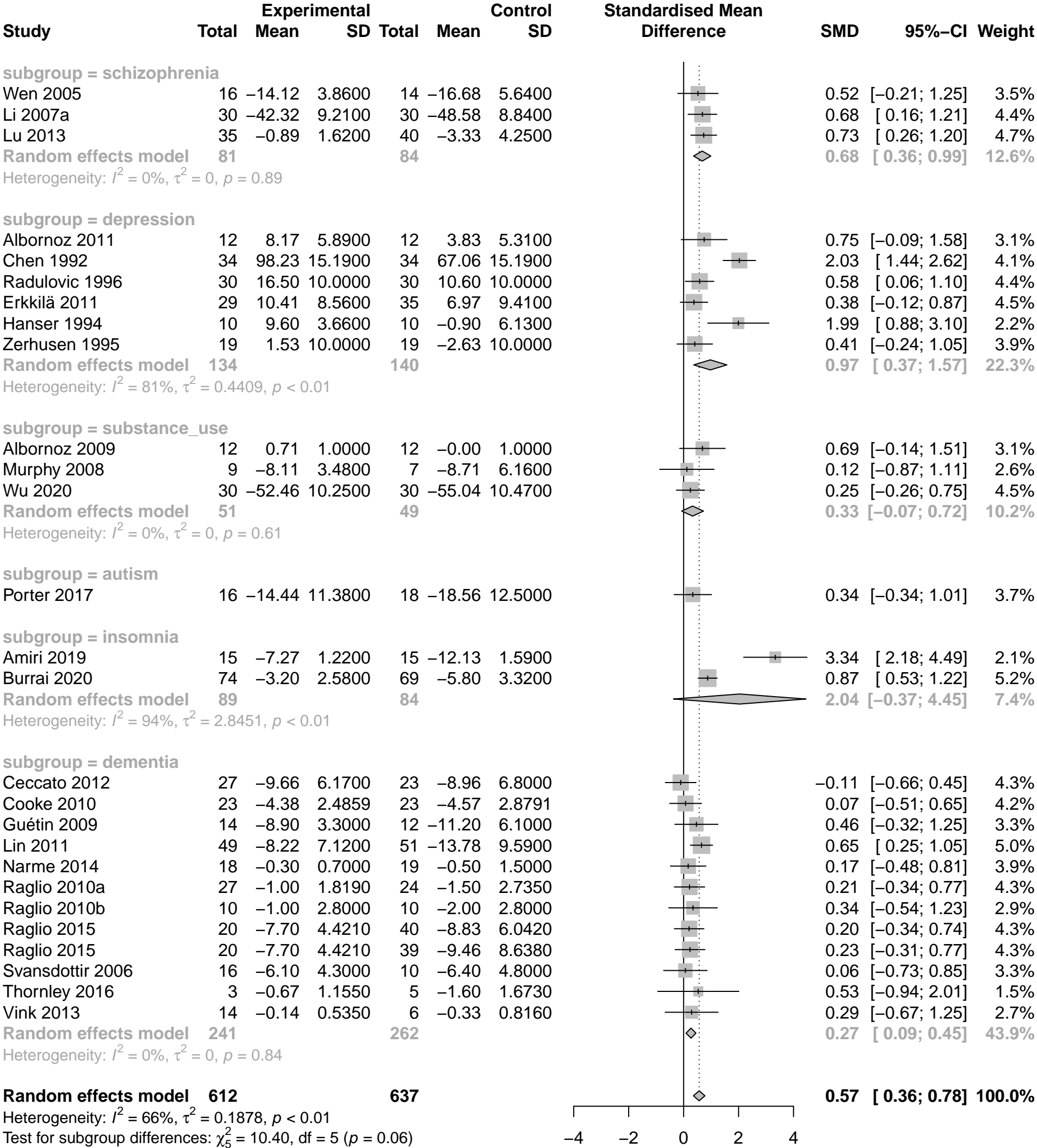

Supplement: Lassner et al. supplementary material 5 — Lassner et al. supplementary material [file S2056472424008263sup005.zip › analysis_2023.08.18/analysis/any depression/any_depression_endpoint.pdf]

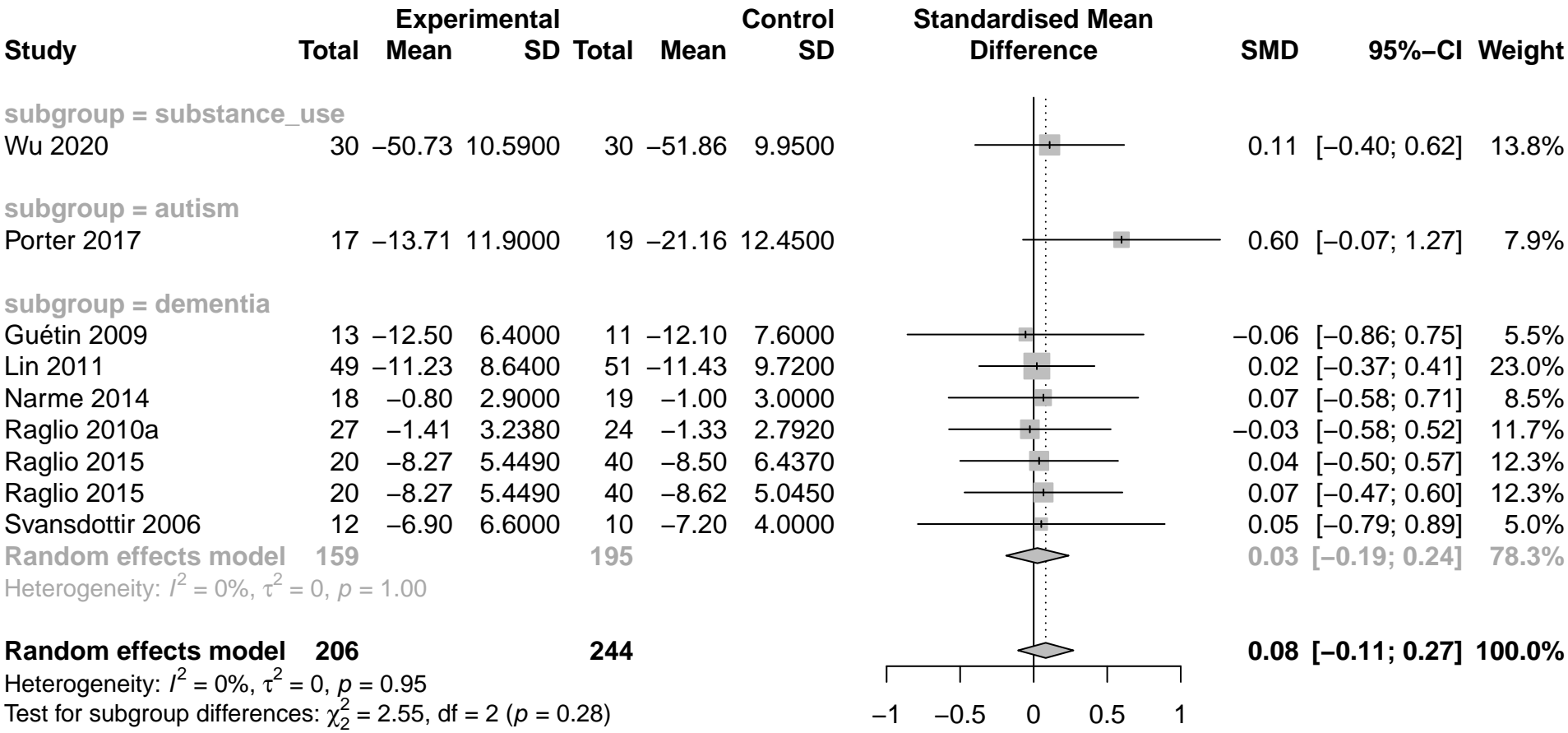

Supplement: Lassner et al. supplementary material 5 — Lassner et al. supplementary material [file S2056472424008263sup005.zip › analysis_2023.08.18/analysis/any depression/any_depression_followup.pdf]

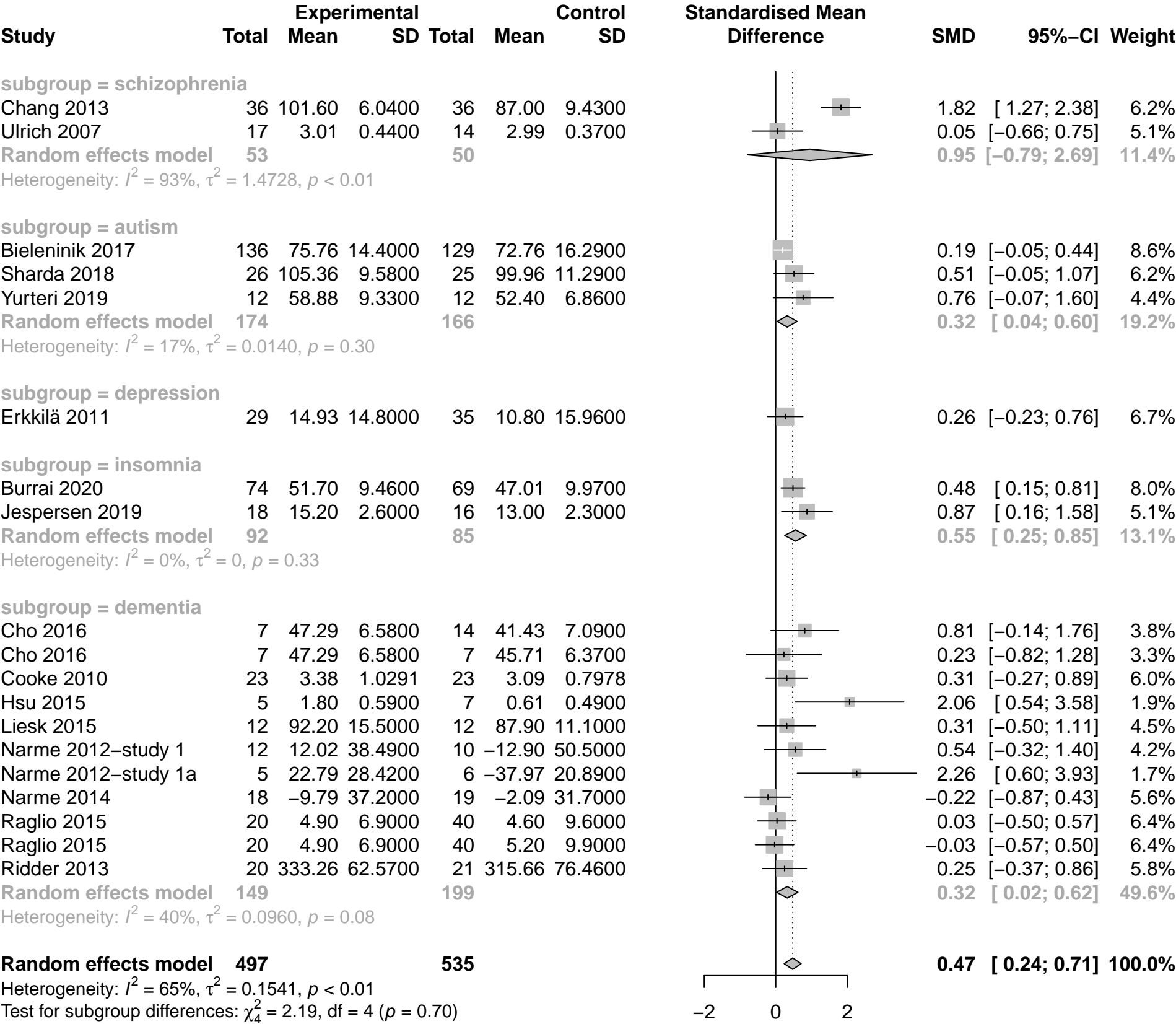

Supplement: Lassner et al. supplementary material 5 — Lassner et al. supplementary material [file S2056472424008263sup005.zip › analysis_2023.08.18/analysis/any qol/any_qol_endpoint.pdf]

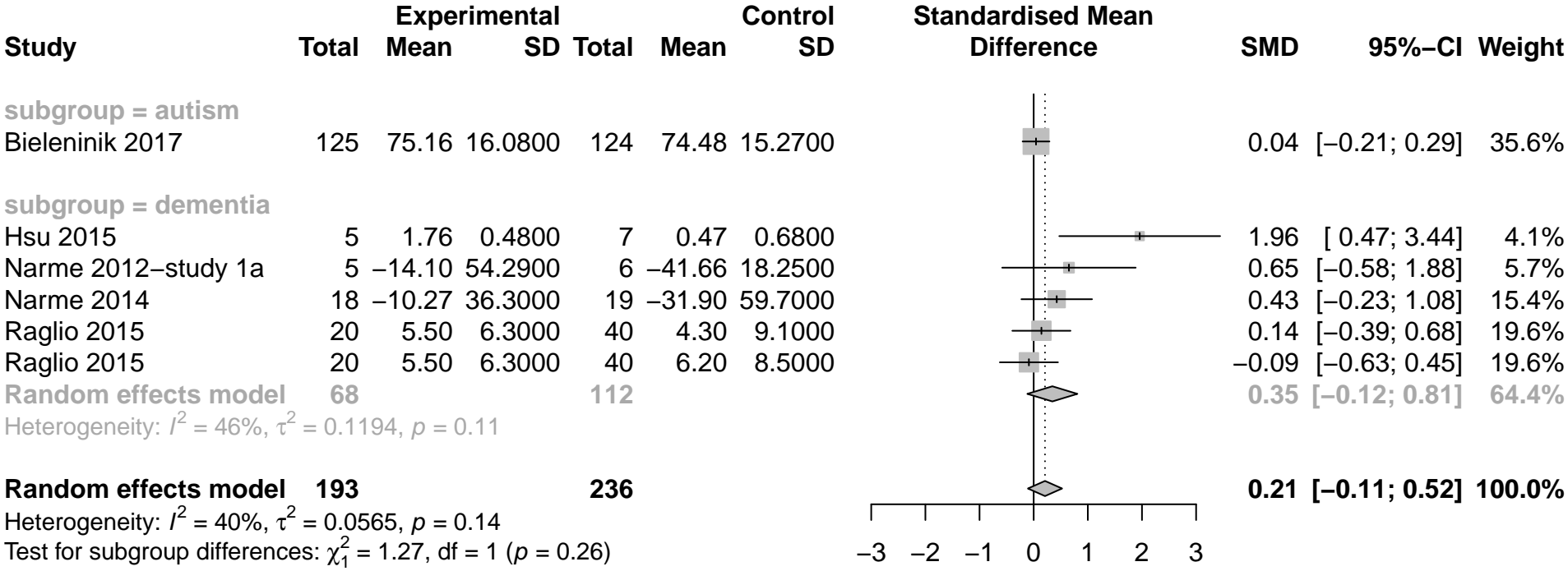

Supplement: Lassner et al. supplementary material 5 — Lassner et al. supplementary material [file S2056472424008263sup005.zip › analysis_2023.08.18/analysis/any qol/any_qol_followup.pdf]

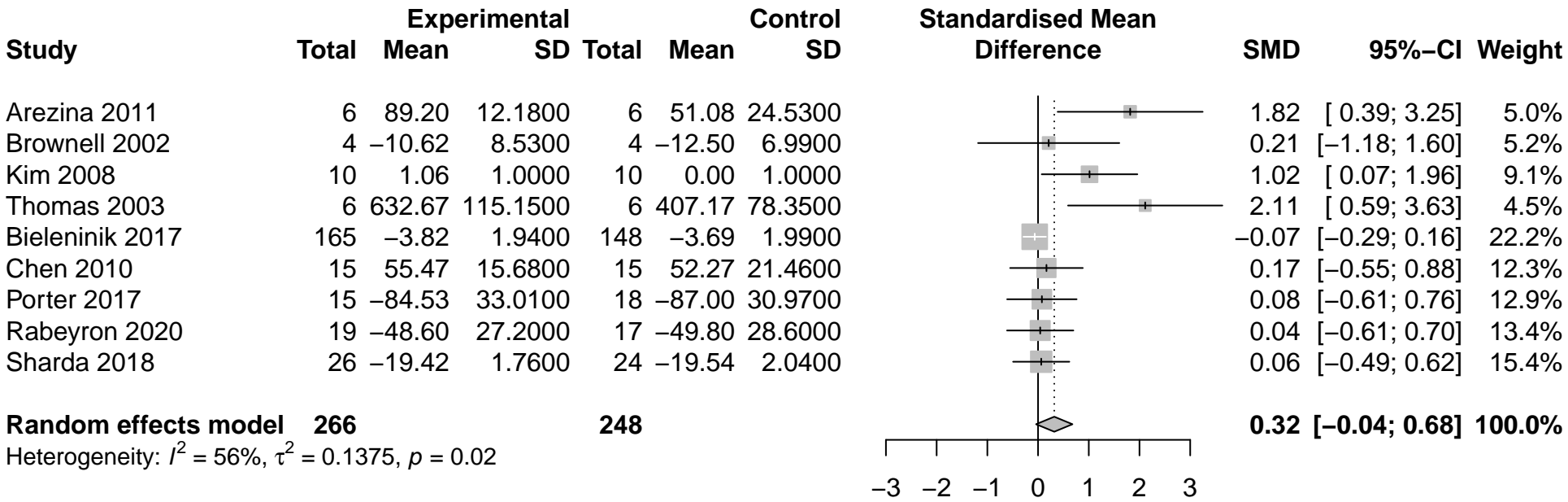

Supplement: Lassner et al. supplementary material 5 — Lassner et al. supplementary material [file S2056472424008263sup005.zip › analysis_2023.08.18/analysis/autism/autism_adaptive_behavior_endpoint.pdf]

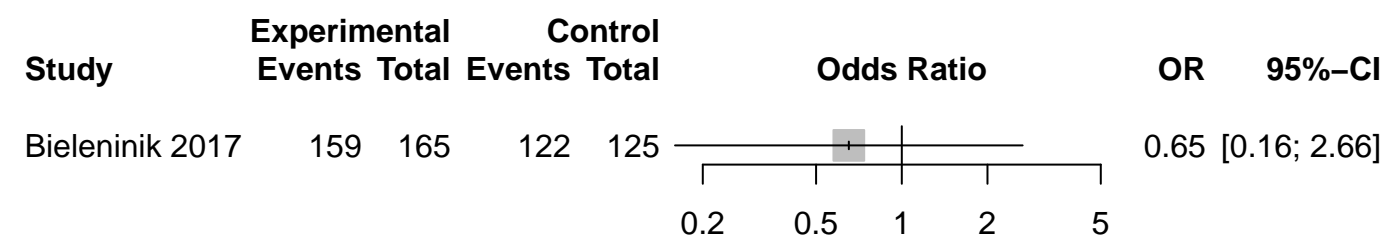

Supplement: Lassner et al. supplementary material 5 — Lassner et al. supplementary material [file S2056472424008263sup005.zip › analysis_2023.08.18/analysis/autism/autism_adverse_event_endpoint.pdf]

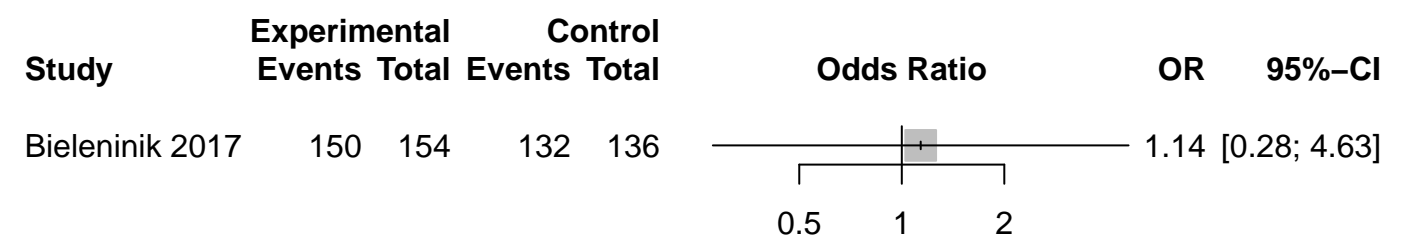

Supplement: Lassner et al. supplementary material 5 — Lassner et al. supplementary material [file S2056472424008263sup005.zip › analysis_2023.08.18/analysis/autism/autism_adverse_event_followup.pdf]

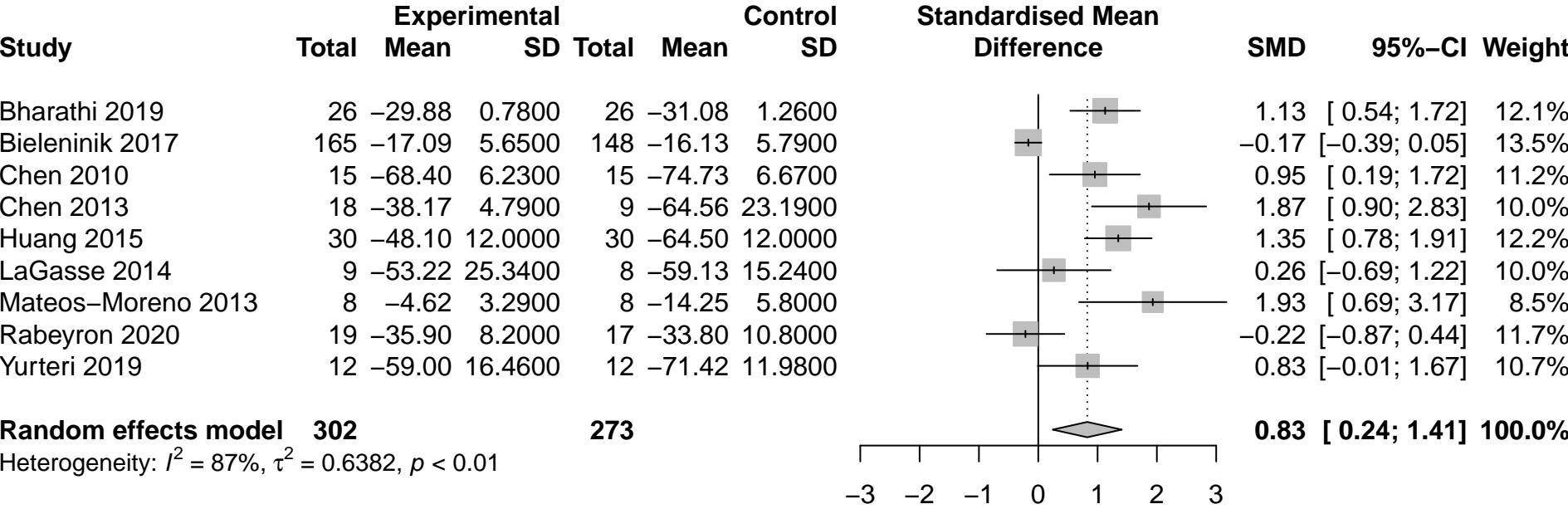

Supplement: Lassner et al. supplementary material 5 — Lassner et al. supplementary material [file S2056472424008263sup005.zip › analysis_2023.08.18/analysis/autism/autism_autism_symptoms_endpoint.pdf]

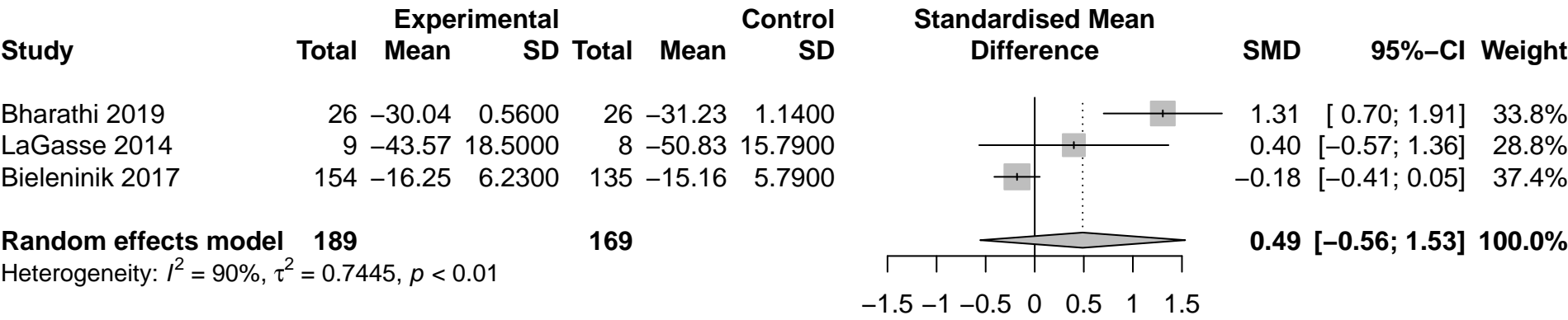

Supplement: Lassner et al. supplementary material 5 — Lassner et al. supplementary material [file S2056472424008263sup005.zip › analysis_2023.08.18/analysis/autism/autism_autism_symptoms_followup.pdf]

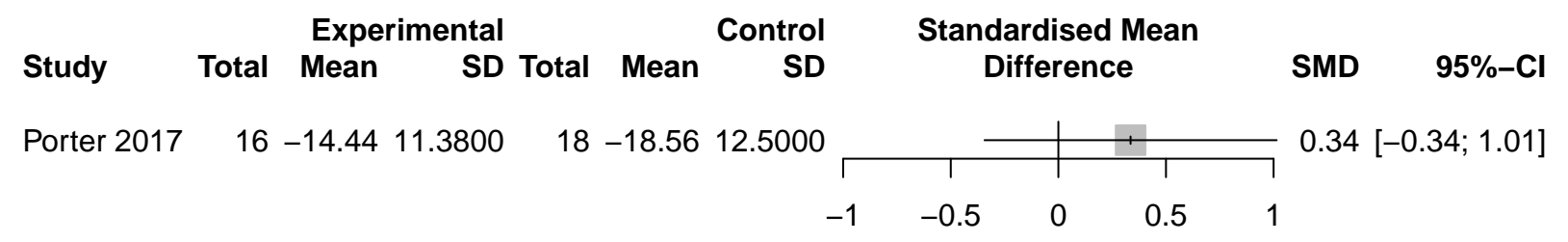

Supplement: Lassner et al. supplementary material 5 — Lassner et al. supplementary material [file S2056472424008263sup005.zip › analysis_2023.08.18/analysis/autism/autism_depression_endpoint.pdf]

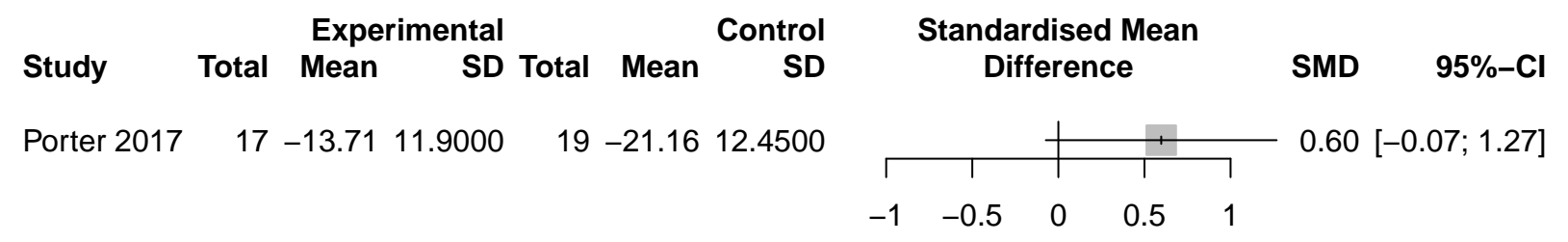

Supplement: Lassner et al. supplementary material 5 — Lassner et al. supplementary material [file S2056472424008263sup005.zip › analysis_2023.08.18/analysis/autism/autism_depression_followup.pdf]

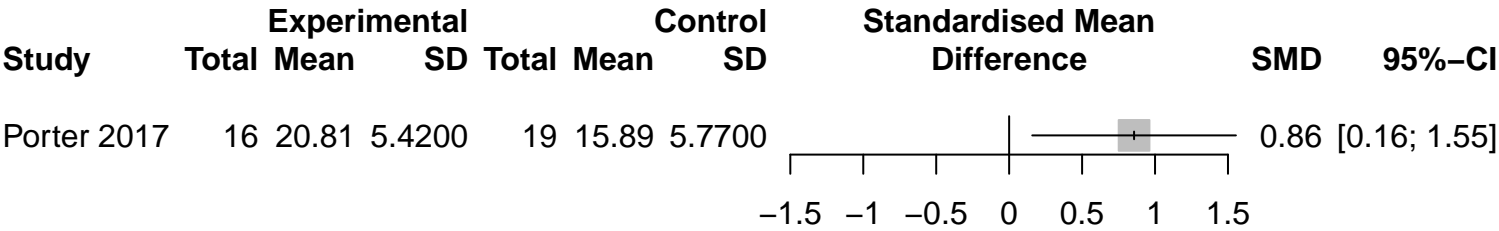

Supplement: Lassner et al. supplementary material 5 — Lassner et al. supplementary material [file S2056472424008263sup005.zip › analysis_2023.08.18/analysis/autism/autism_identify_formation_followup.pdf]

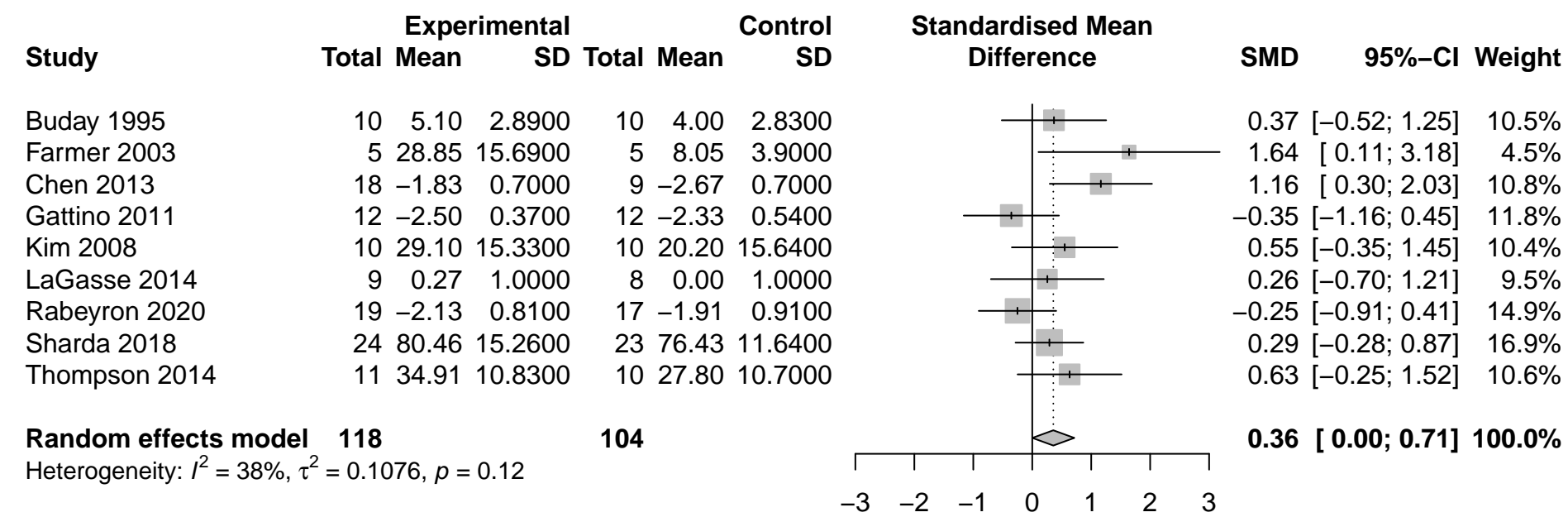

Supplement: Lassner et al. supplementary material 5 — Lassner et al. supplementary material [file S2056472424008263sup005.zip › analysis_2023.08.18/analysis/autism/autism_non-verbal_endpoint.pdf]

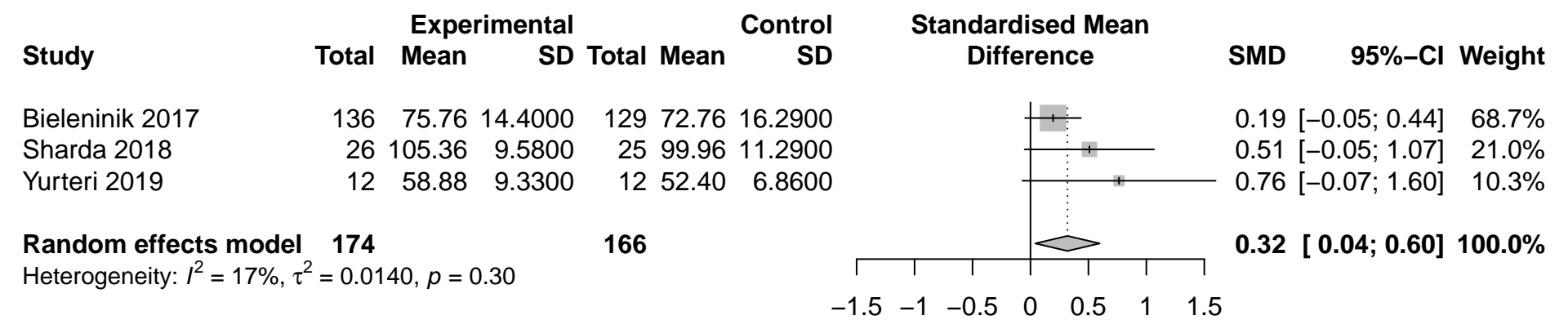

Supplement: Lassner et al. supplementary material 5 — Lassner et al. supplementary material [file S2056472424008263sup005.zip › analysis_2023.08.18/analysis/autism/autism_qol_endpoint.pdf]

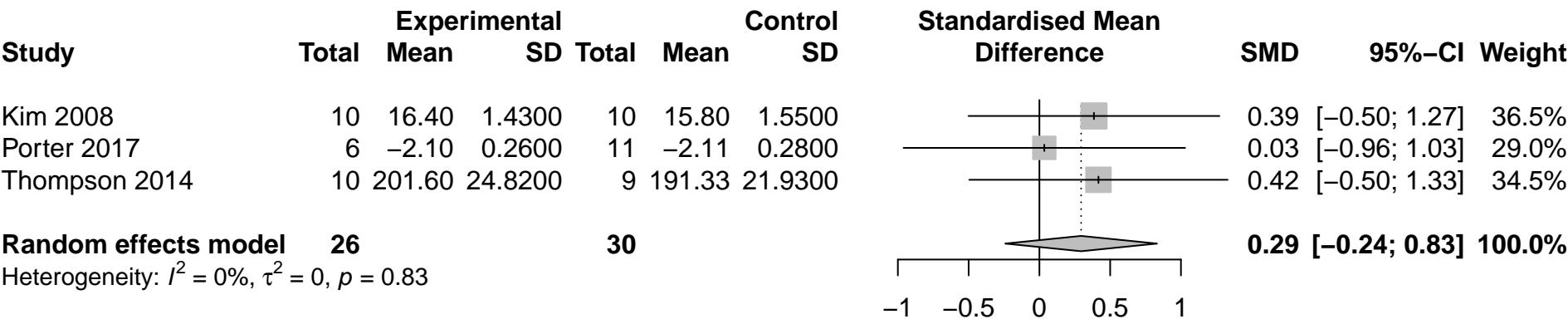

Supplement: Lassner et al. supplementary material 5 — Lassner et al. supplementary material [file S2056472424008263sup005.zip › analysis_2023.08.18/analysis/autism/autism_qol_family_relationships_endpoint.pdf]

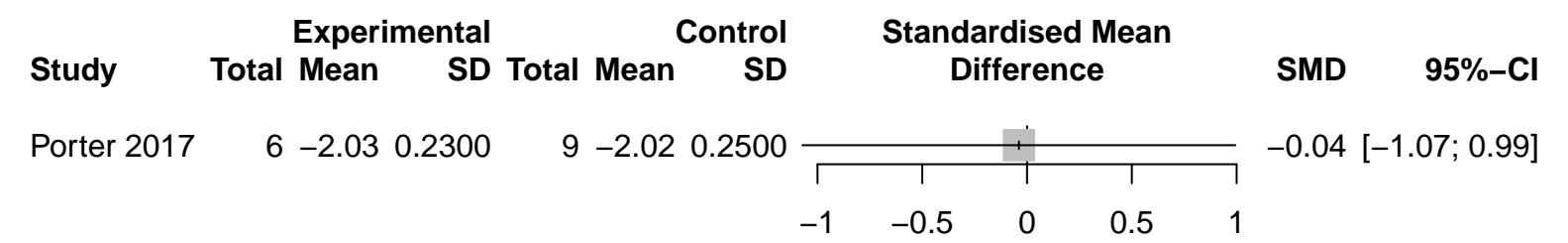

Supplement: Lassner et al. supplementary material 5 — Lassner et al. supplementary material [file S2056472424008263sup005.zip › analysis_2023.08.18/analysis/autism/autism_qol_family_relationships_followup.pdf]

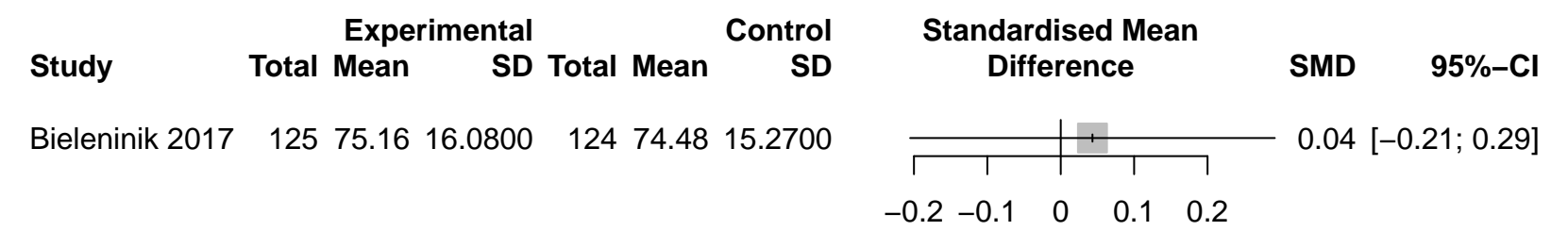

Supplement: Lassner et al. supplementary material 5 — Lassner et al. supplementary material [file S2056472424008263sup005.zip › analysis_2023.08.18/analysis/autism/autism_qol_followup.pdf]

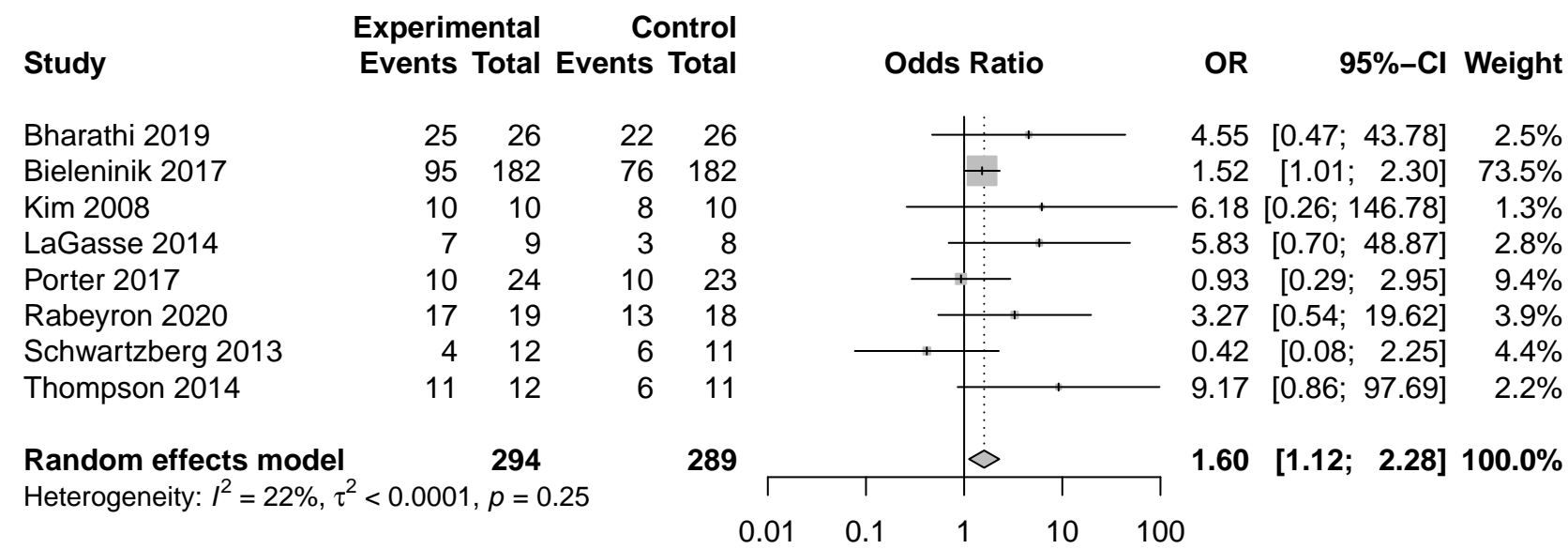

Supplement: Lassner et al. supplementary material 5 — Lassner et al. supplementary material [file S2056472424008263sup005.zip › analysis_2023.08.18/analysis/autism/autism_response_endpoint.pdf]

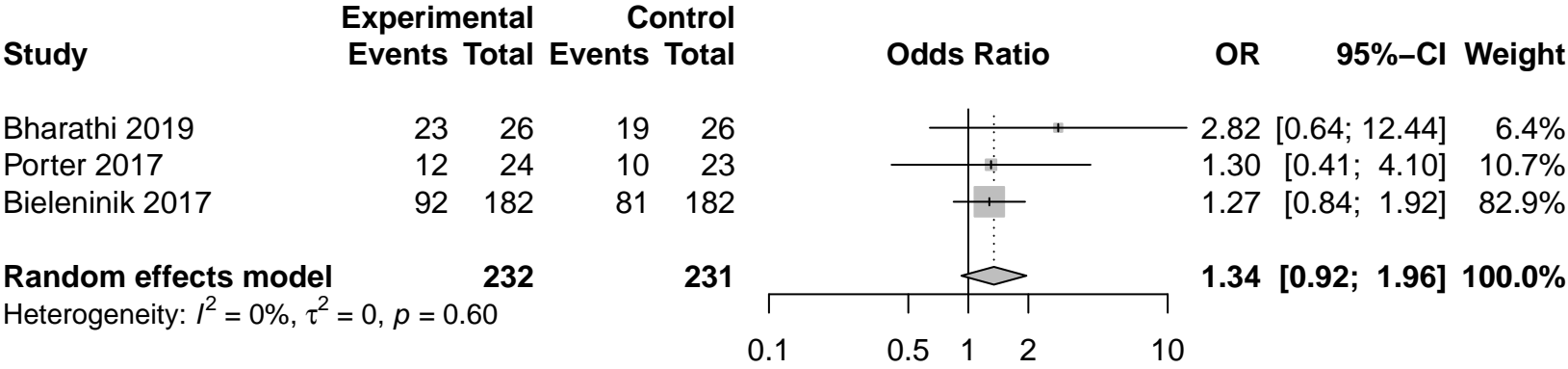

Supplement: Lassner et al. supplementary material 5 — Lassner et al. supplementary material [file S2056472424008263sup005.zip › analysis_2023.08.18/analysis/autism/autism_response_followup.pdf]

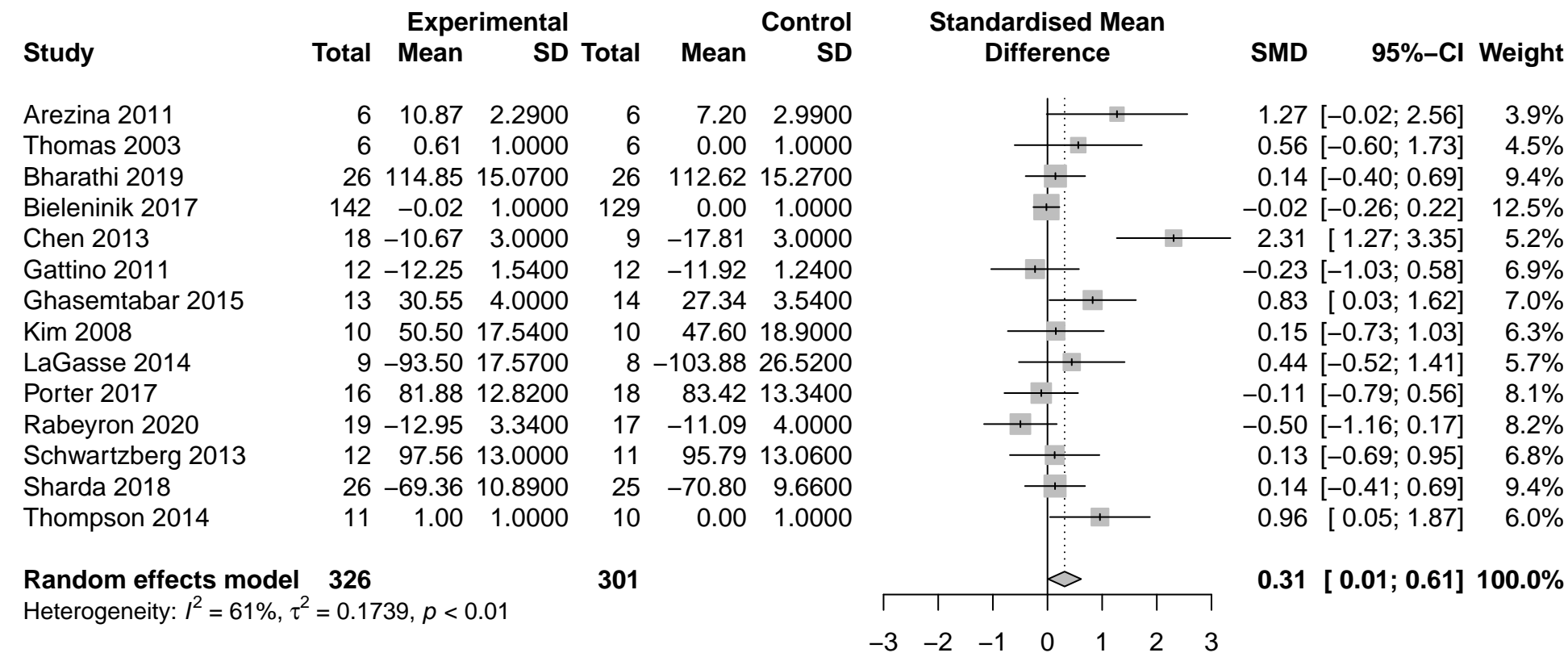

Supplement: Lassner et al. supplementary material 5 — Lassner et al. supplementary material [file S2056472424008263sup005.zip › analysis_2023.08.18/analysis/autism/autism_social interaction_endpoint.pdf]

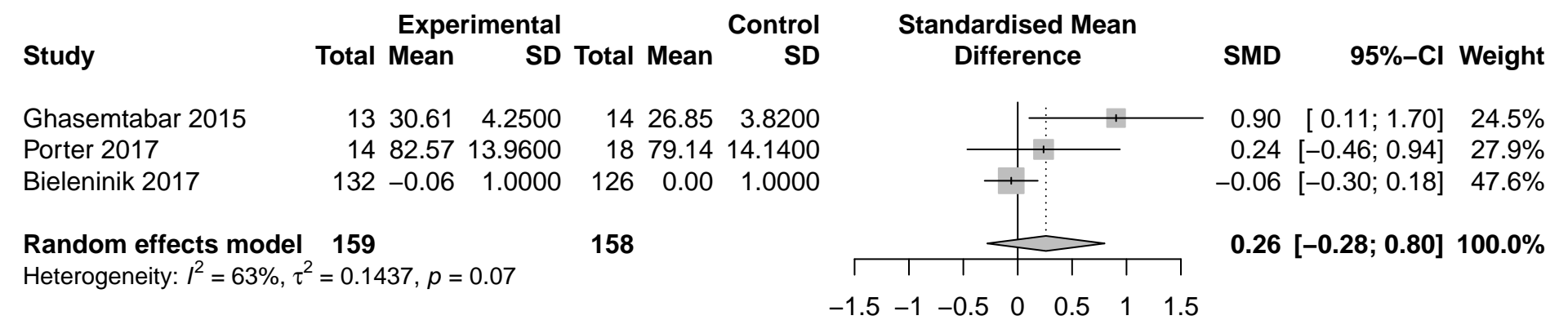

Supplement: Lassner et al. supplementary material 5 — Lassner et al. supplementary material [file S2056472424008263sup005.zip › analysis_2023.08.18/analysis/autism/autism_social interaction_followup.pdf]

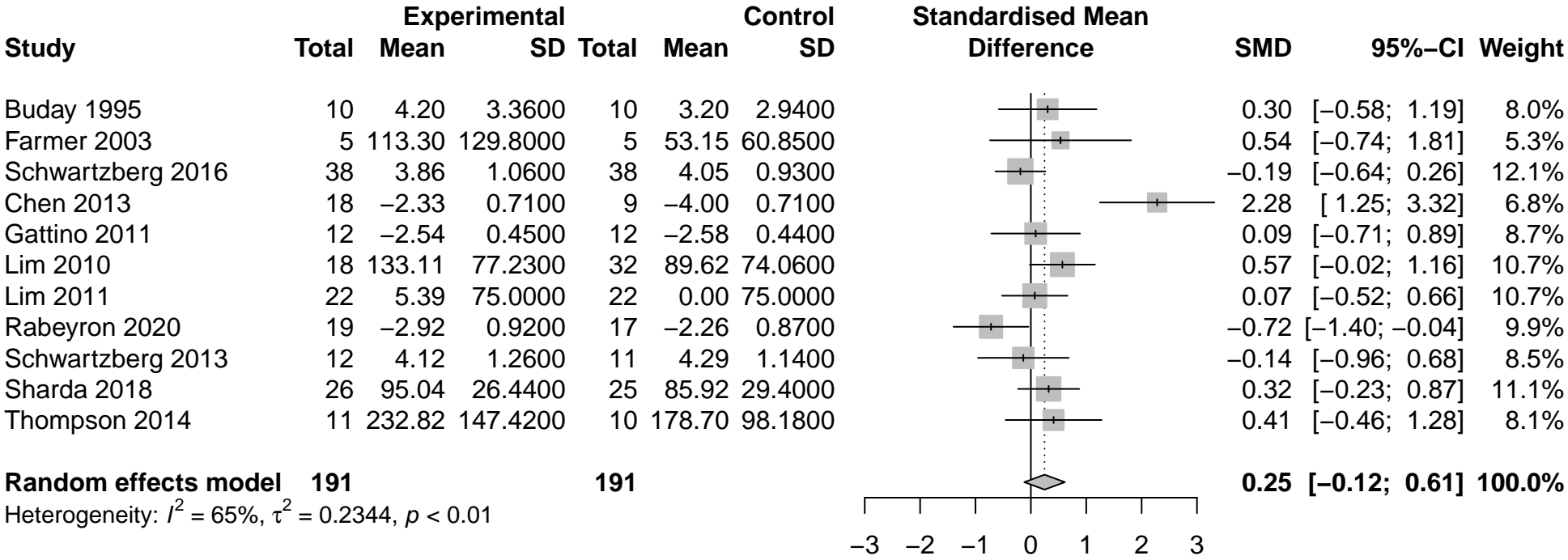

Supplement: Lassner et al. supplementary material 5 — Lassner et al. supplementary material [file S2056472424008263sup005.zip › analysis_2023.08.18/analysis/autism/autism_verbal_endpoint.pdf]

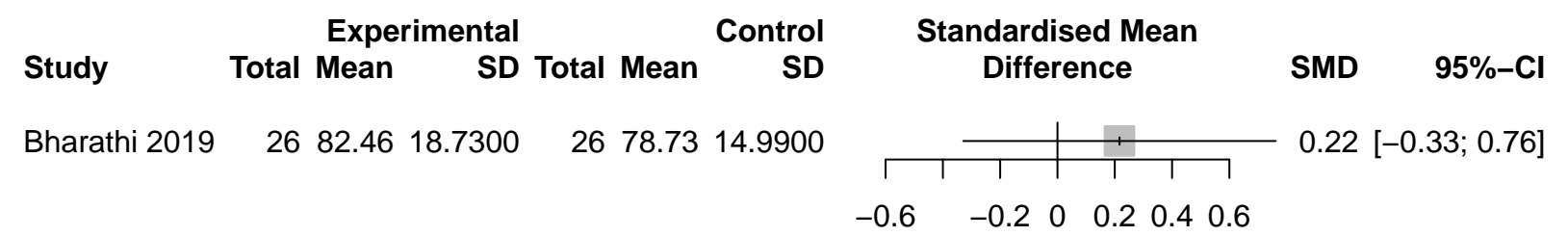

Supplement: Lassner et al. supplementary material 5 — Lassner et al. supplementary material [file S2056472424008263sup005.zip › analysis_2023.08.18/analysis/autism/autism_verbal_followup.pdf]

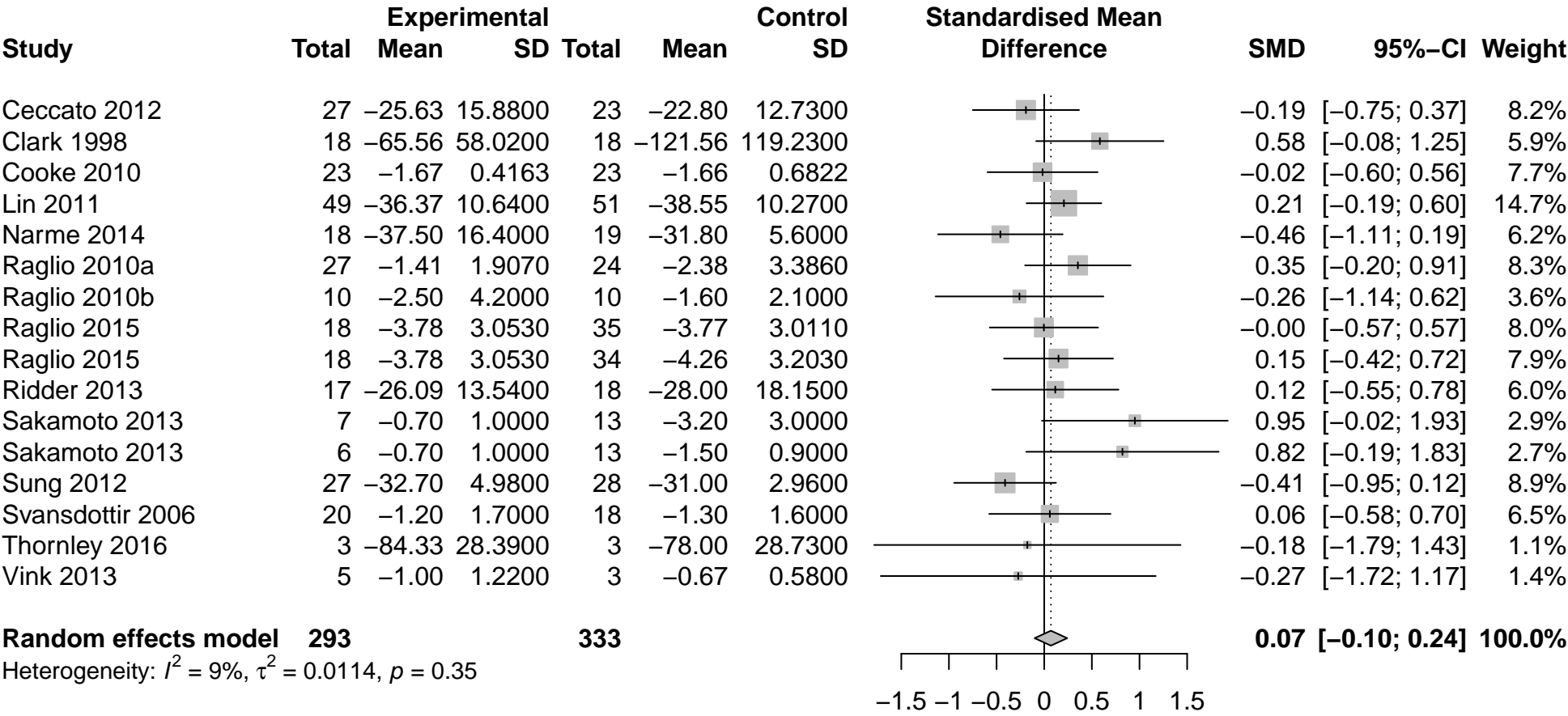

Supplement: Lassner et al. supplementary material 5 — Lassner et al. supplementary material [file S2056472424008263sup005.zip › analysis_2023.08.18/analysis/dementia/dementia_agitation-aggression_endpoint.pdf]

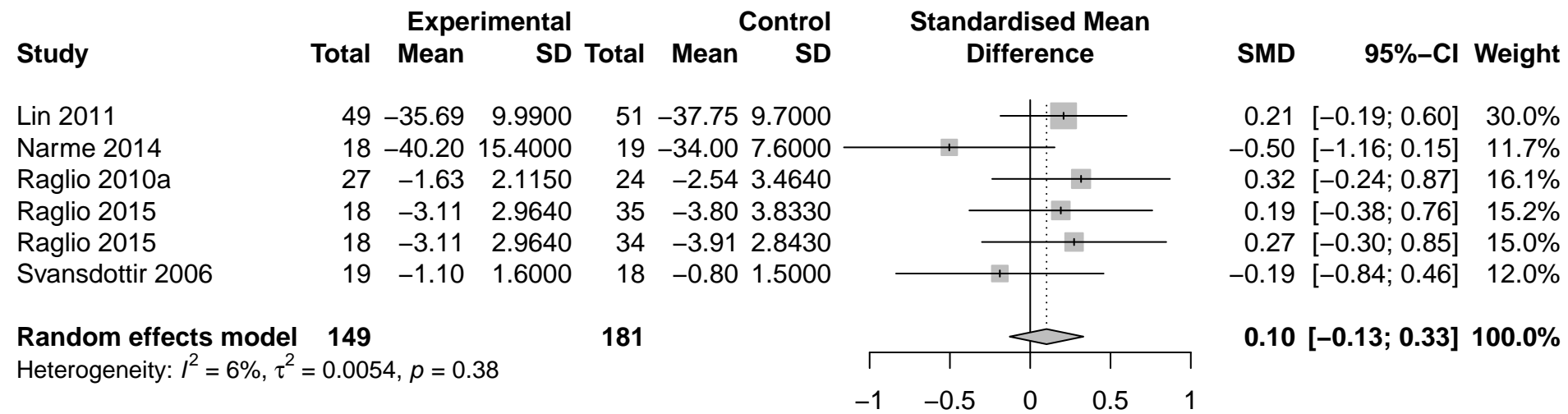

Supplement: Lassner et al. supplementary material 5 — Lassner et al. supplementary material [file S2056472424008263sup005.zip › analysis_2023.08.18/analysis/dementia/dementia_agitation-aggression_followup.pdf]

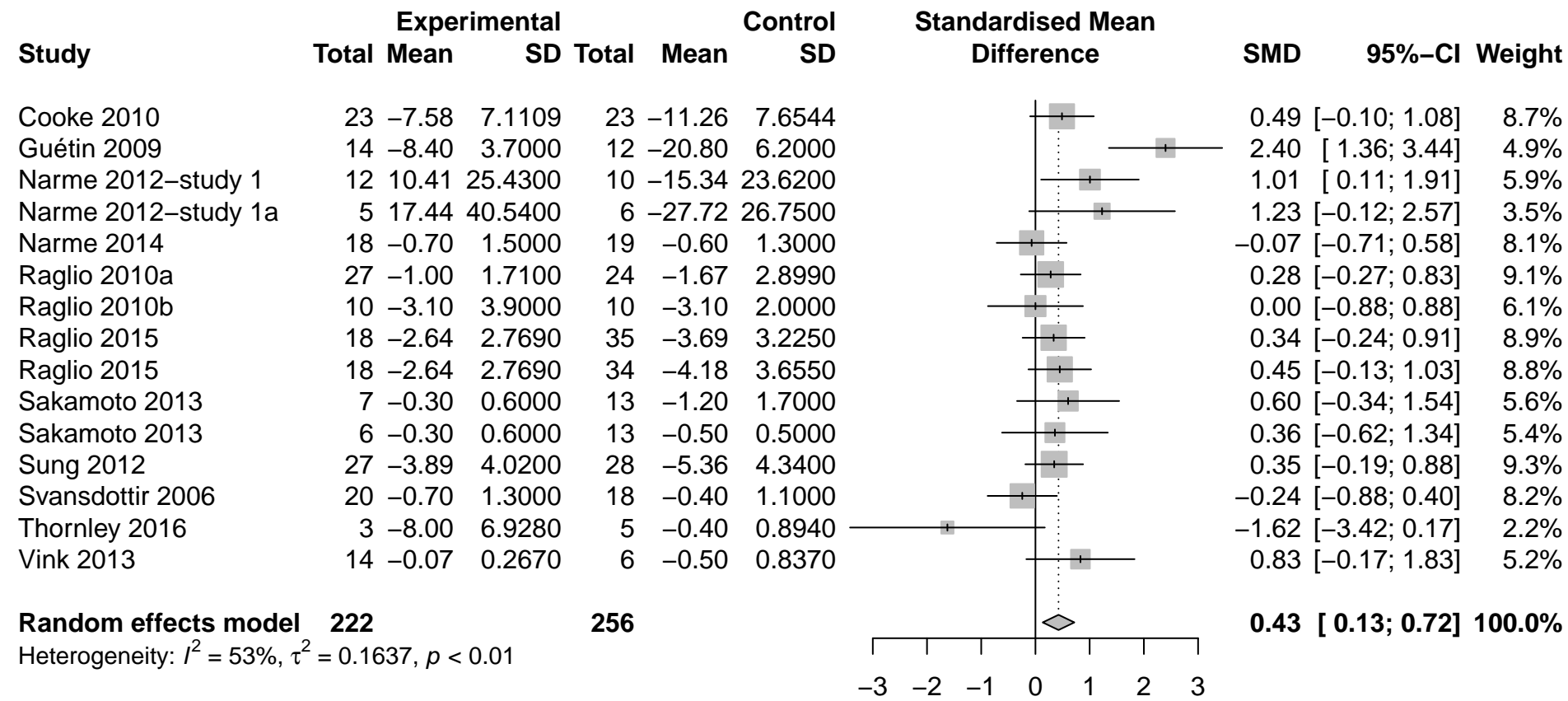

Supplement: Lassner et al. supplementary material 5 — Lassner et al. supplementary material [file S2056472424008263sup005.zip › analysis_2023.08.18/analysis/dementia/dementia_anxiety_endpoint.pdf]

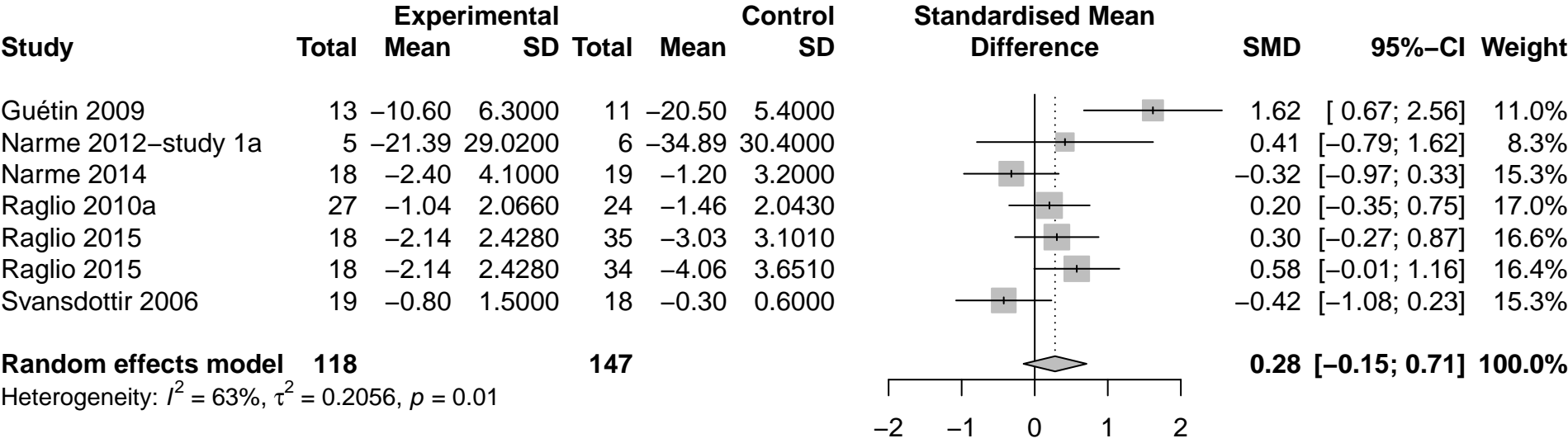

Supplement: Lassner et al. supplementary material 5 — Lassner et al. supplementary material [file S2056472424008263sup005.zip › analysis_2023.08.18/analysis/dementia/dementia_anxiety_followup.pdf]

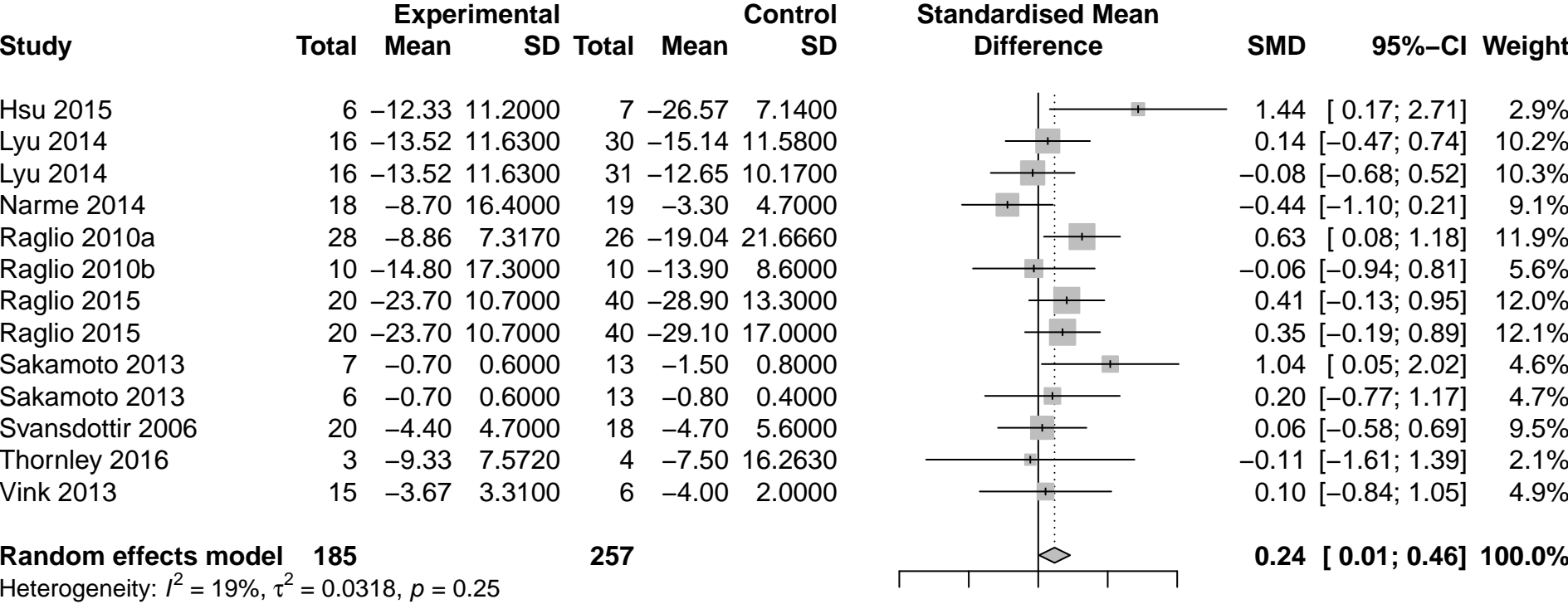

Supplement: Lassner et al. supplementary material 5 — Lassner et al. supplementary material [file S2056472424008263sup005.zip › analysis_2023.08.18/analysis/dementia/dementia_behavioral_problems_overall_endpoint.pdf]

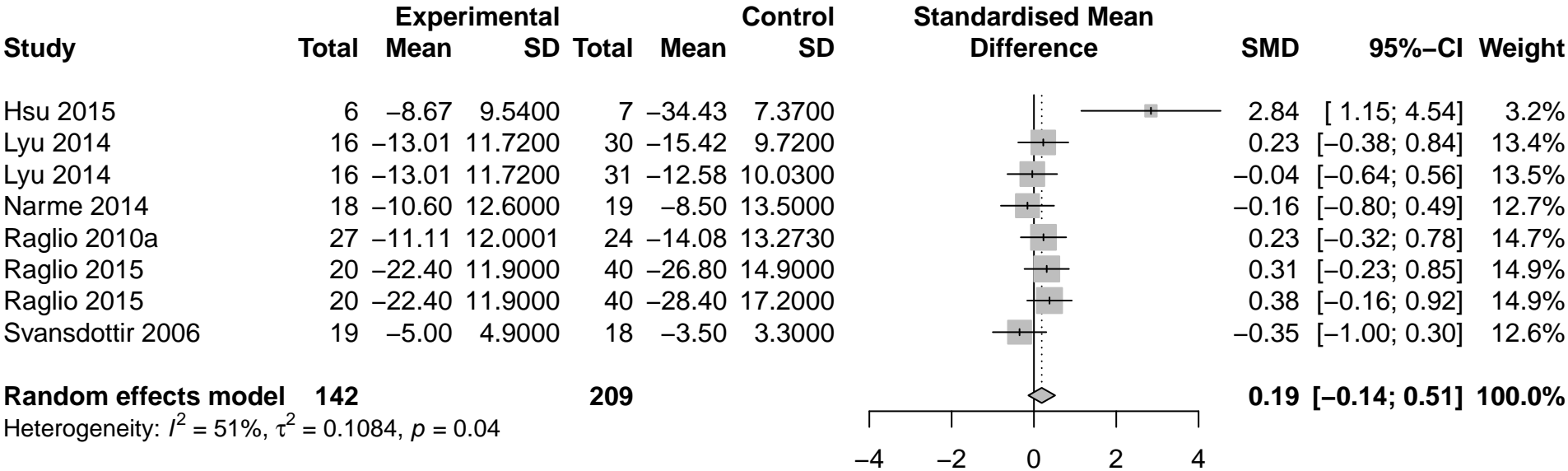

Supplement: Lassner et al. supplementary material 5 — Lassner et al. supplementary material [file S2056472424008263sup005.zip › analysis_2023.08.18/analysis/dementia/dementia_behavioral_problems_overall_followup.pdf]

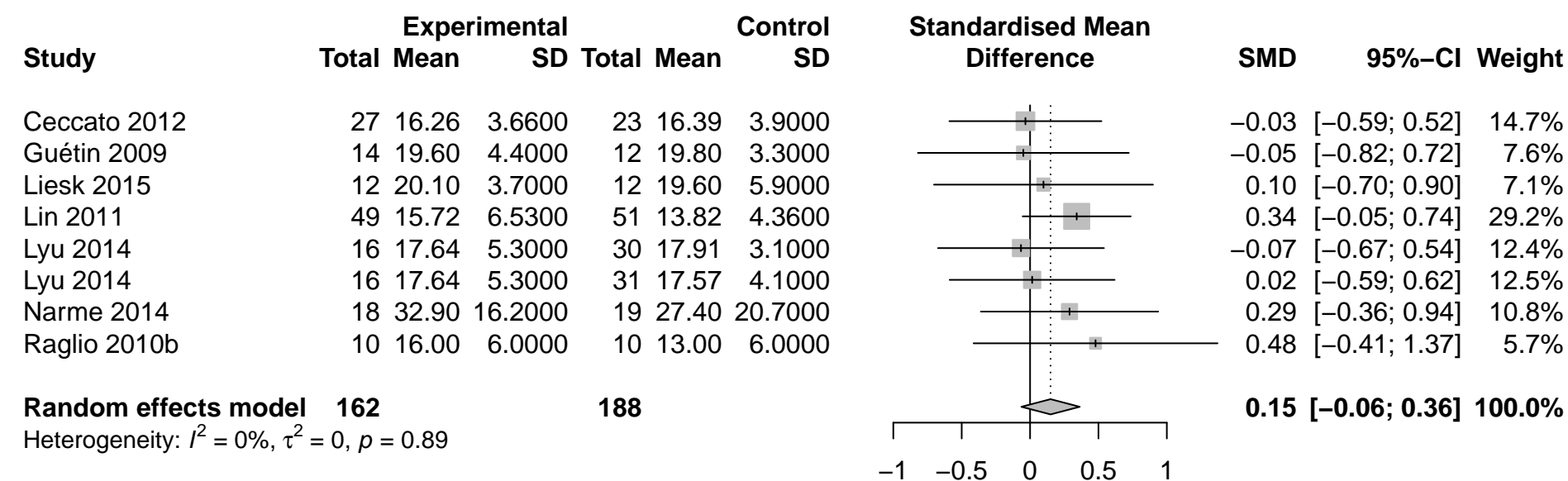

Supplement: Lassner et al. supplementary material 5 — Lassner et al. supplementary material [file S2056472424008263sup005.zip › analysis_2023.08.18/analysis/dementia/dementia_cognition_endpoint.pdf]

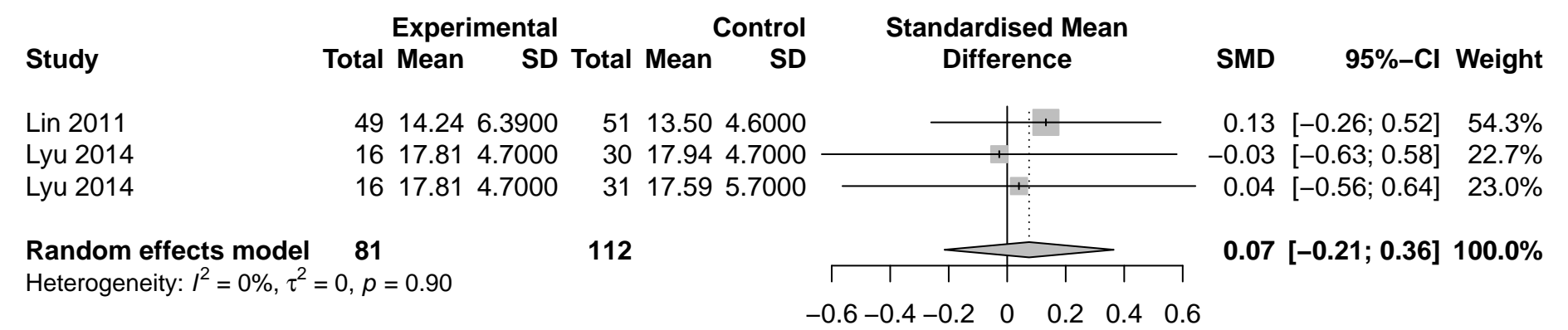

Supplement: Lassner et al. supplementary material 5 — Lassner et al. supplementary material [file S2056472424008263sup005.zip › analysis_2023.08.18/analysis/dementia/dementia_cognition_followup.pdf]

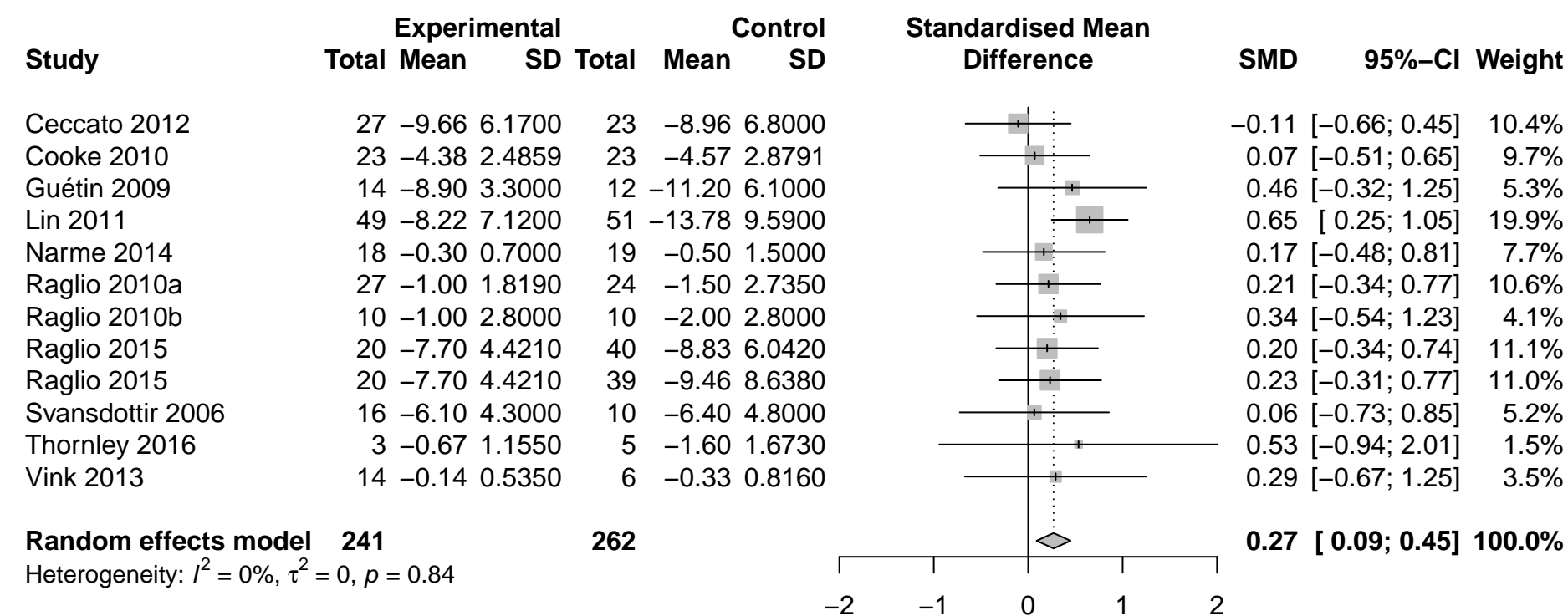

Supplement: Lassner et al. supplementary material 5 — Lassner et al. supplementary material [file S2056472424008263sup005.zip › analysis_2023.08.18/analysis/dementia/dementia_depression_endpoint.pdf]

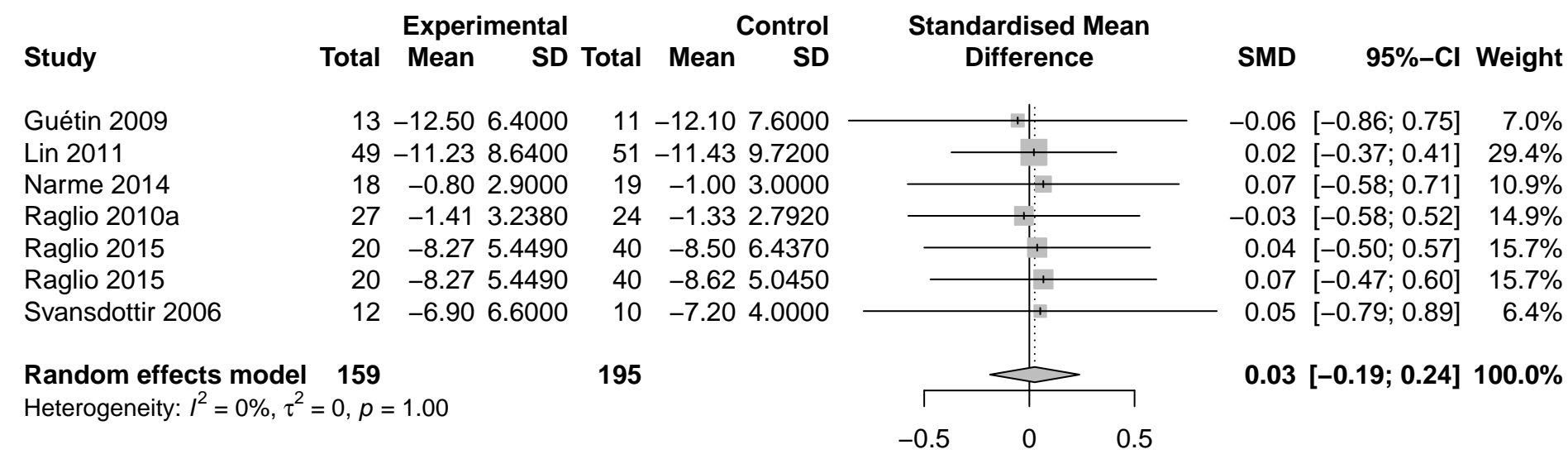

Supplement: Lassner et al. supplementary material 5 — Lassner et al. supplementary material [file S2056472424008263sup005.zip › analysis_2023.08.18/analysis/dementia/dementia_depression_followup.pdf]

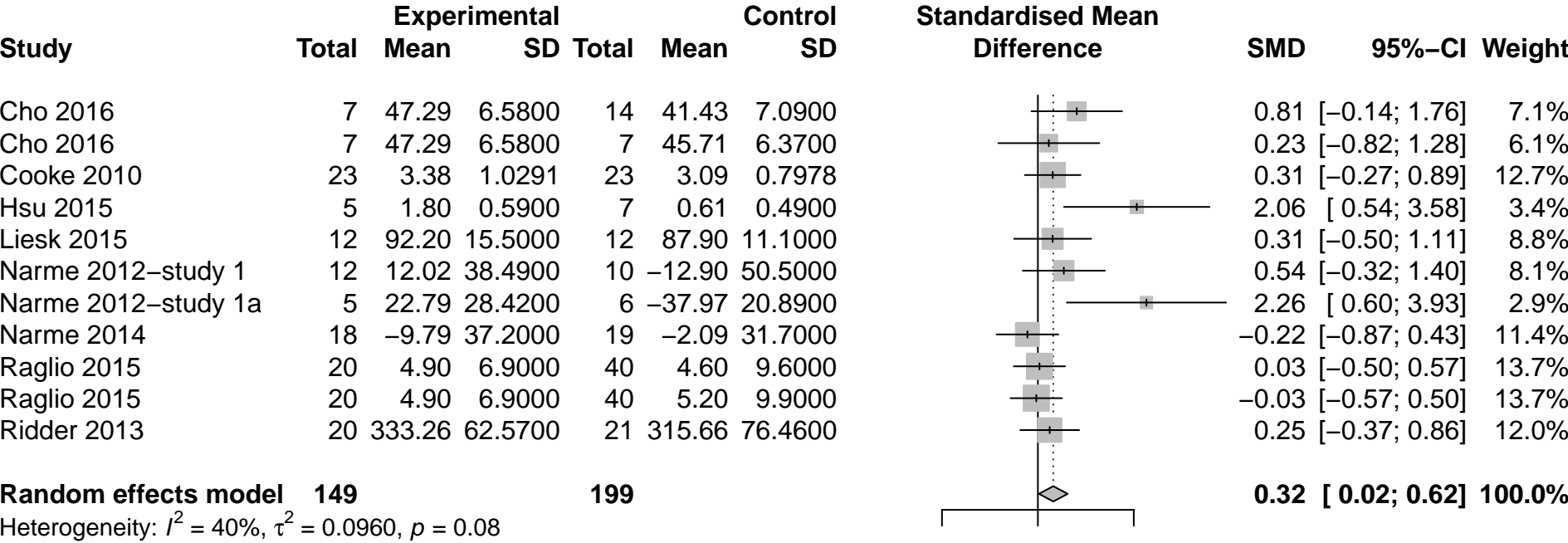

Supplement: Lassner et al. supplementary material 5 — Lassner et al. supplementary material [file S2056472424008263sup005.zip › analysis_2023.08.18/analysis/dementia/dementia_qol_endpoint.pdf]

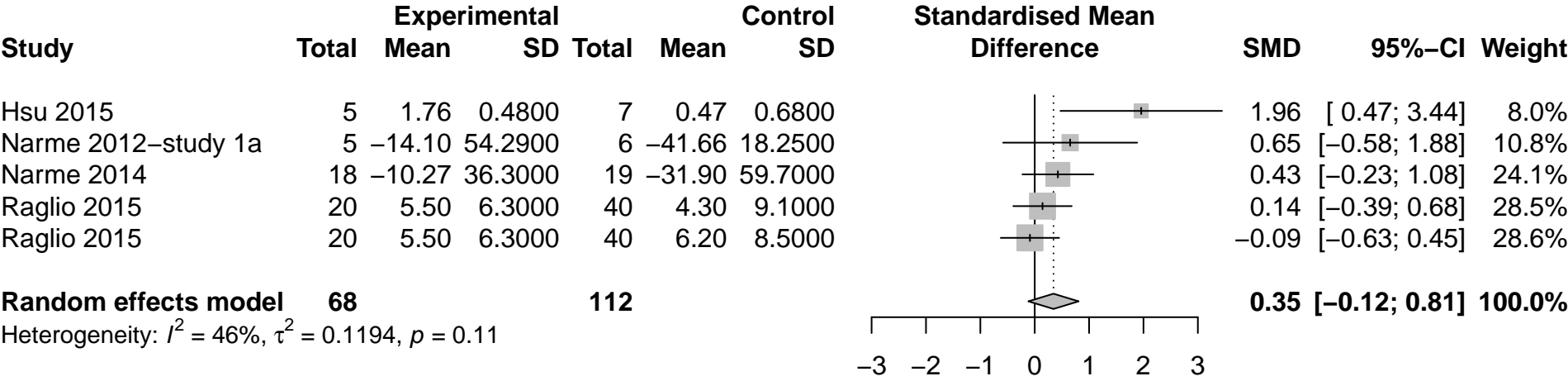

Supplement: Lassner et al. supplementary material 5 — Lassner et al. supplementary material [file S2056472424008263sup005.zip › analysis_2023.08.18/analysis/dementia/dementia_qol_followup.pdf]

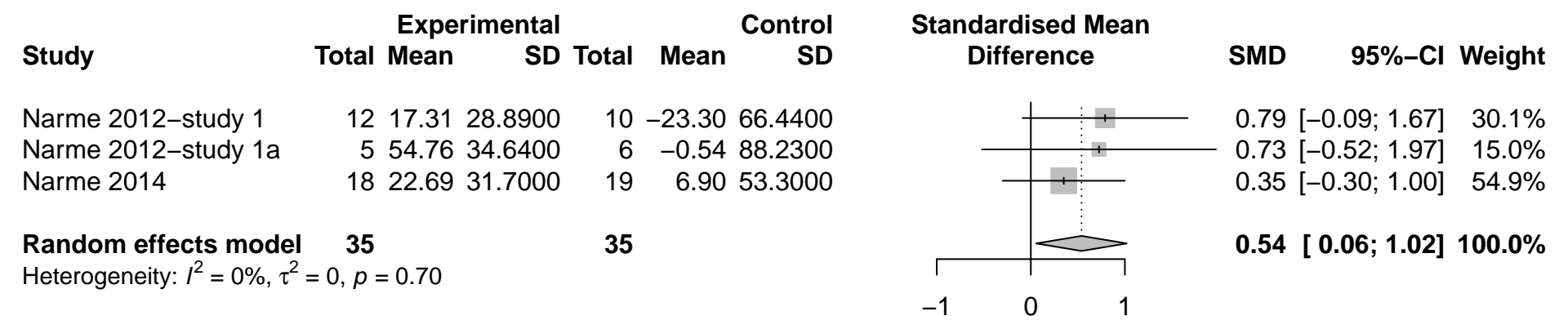

Supplement: Lassner et al. supplementary material 5 — Lassner et al. supplementary material [file S2056472424008263sup005.zip › analysis_2023.08.18/analysis/dementia/dementia_social_behavior_endpoint.pdf]

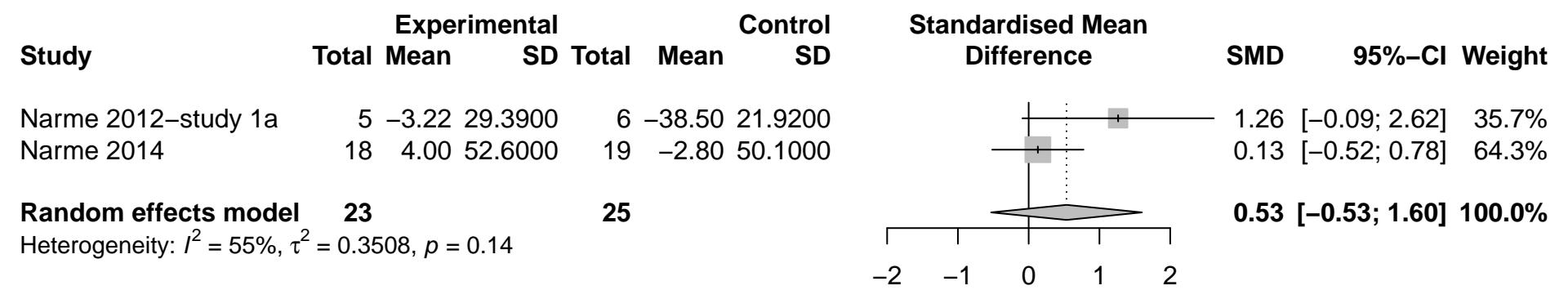

Supplement: Lassner et al. supplementary material 5 — Lassner et al. supplementary material [file S2056472424008263sup005.zip › analysis_2023.08.18/analysis/dementia/dementia_social_behavior_followup.pdf]

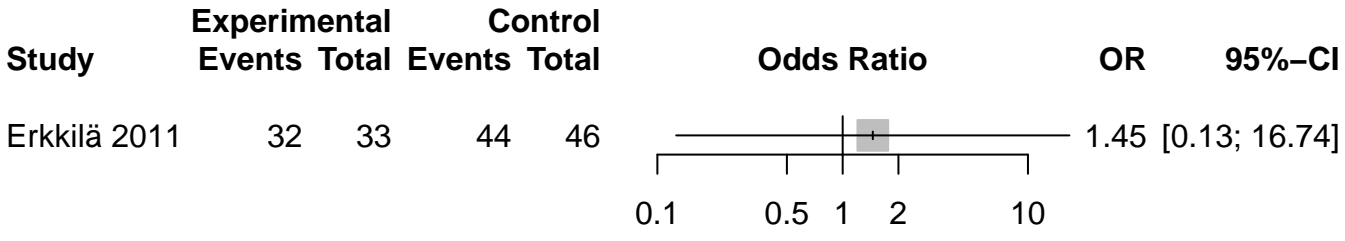

Supplement: Lassner et al. supplementary material 5 — Lassner et al. supplementary material [file S2056472424008263sup005.zip › analysis_2023.08.18/analysis/depression/depression_adverse_event_endpoint.pdf]

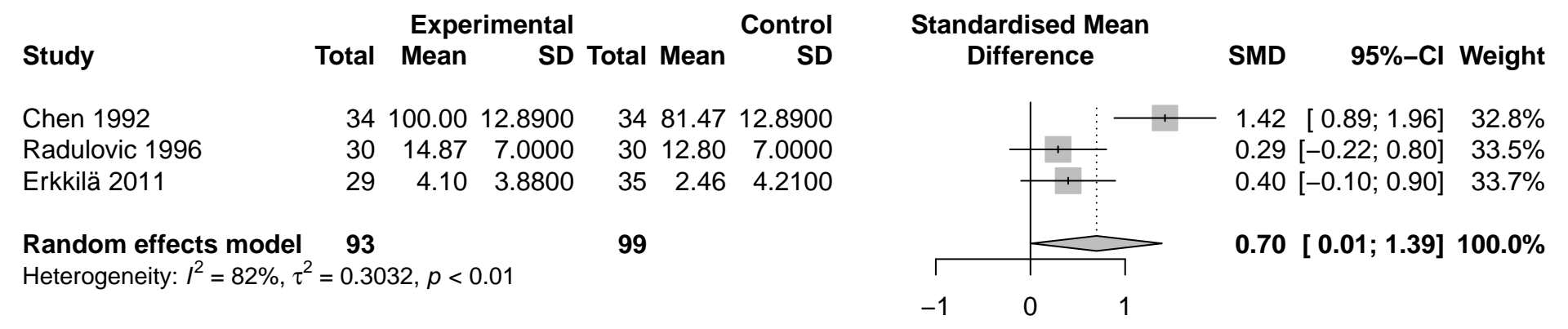

Supplement: Lassner et al. supplementary material 5 — Lassner et al. supplementary material [file S2056472424008263sup005.zip › analysis_2023.08.18/analysis/depression/depression_anxiety_endpoint.pdf]

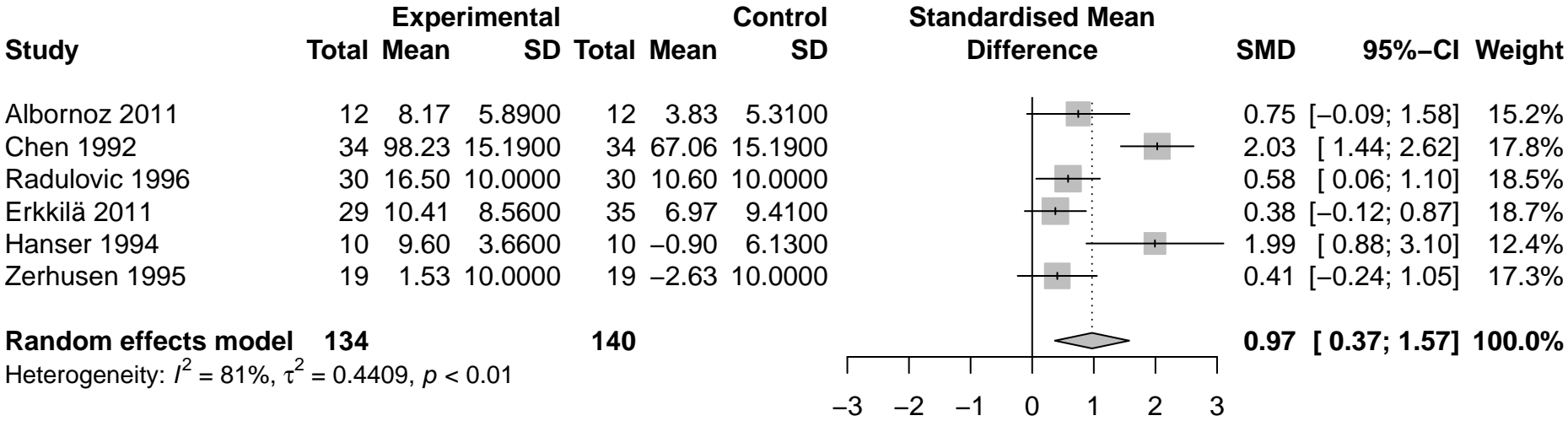

Supplement: Lassner et al. supplementary material 5 — Lassner et al. supplementary material [file S2056472424008263sup005.zip › analysis_2023.08.18/analysis/depression/depression_depression_endpoint.pdf]

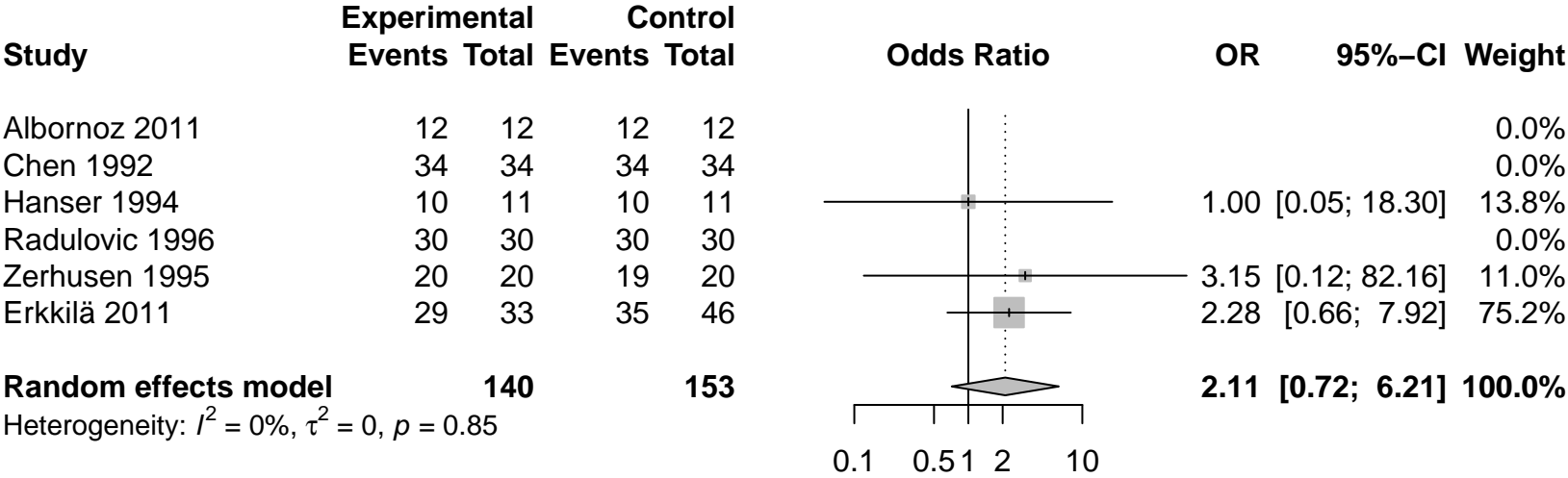

Supplement: Lassner et al. supplementary material 5 — Lassner et al. supplementary material [file S2056472424008263sup005.zip › analysis_2023.08.18/analysis/depression/depression_dropout_any_endpoint.pdf]

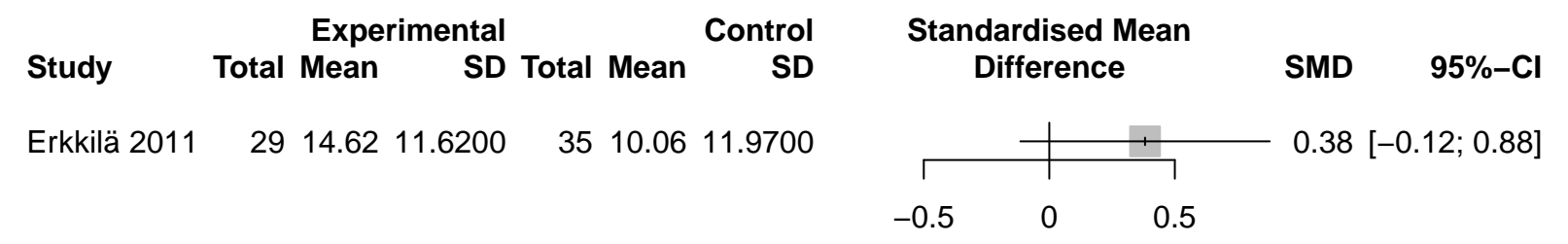

Supplement: Lassner et al. supplementary material 5 — Lassner et al. supplementary material [file S2056472424008263sup005.zip › analysis_2023.08.18/analysis/depression/depression_functioning_endpoint.pdf]

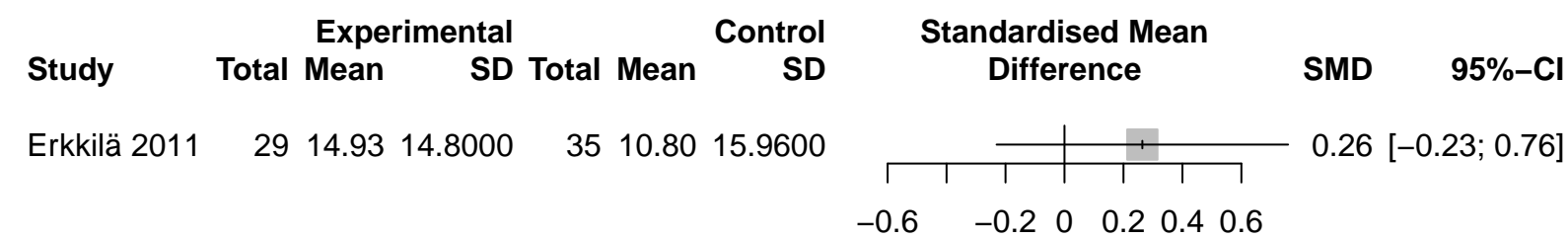

Supplement: Lassner et al. supplementary material 5 — Lassner et al. supplementary material [file S2056472424008263sup005.zip › analysis_2023.08.18/analysis/depression/depression_qol_endpoint.pdf]

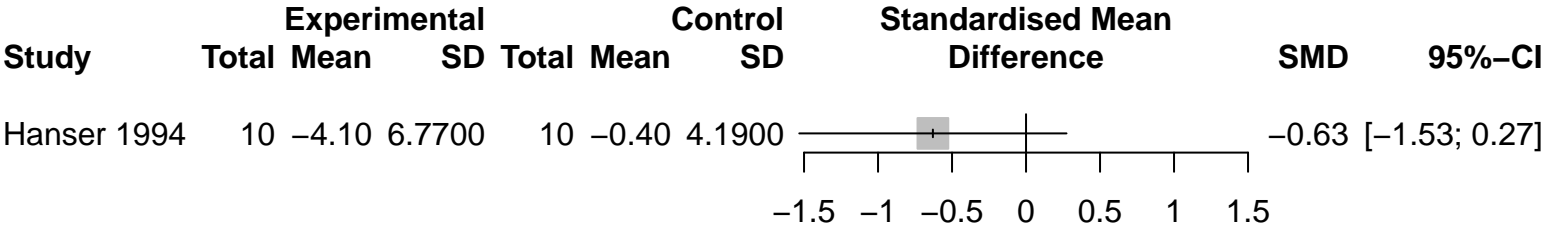

Supplement: Lassner et al. supplementary material 5 — Lassner et al. supplementary material [file S2056472424008263sup005.zip › analysis_2023.08.18/analysis/depression/depression_self_esteem_endpoint.pdf]

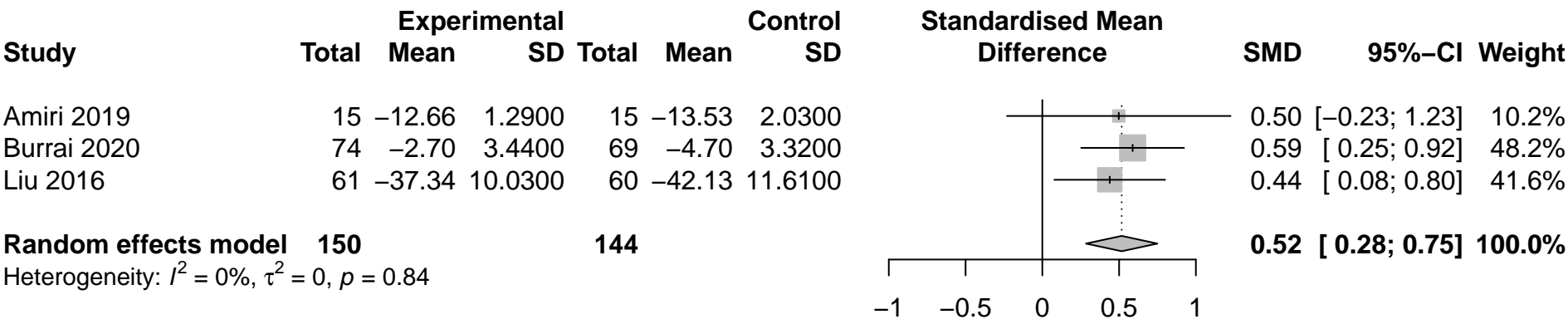

Supplement: Lassner et al. supplementary material 5 — Lassner et al. supplementary material [file S2056472424008263sup005.zip › analysis_2023.08.18/analysis/insomnia/insomnia_anxiety_endpoint.pdf]

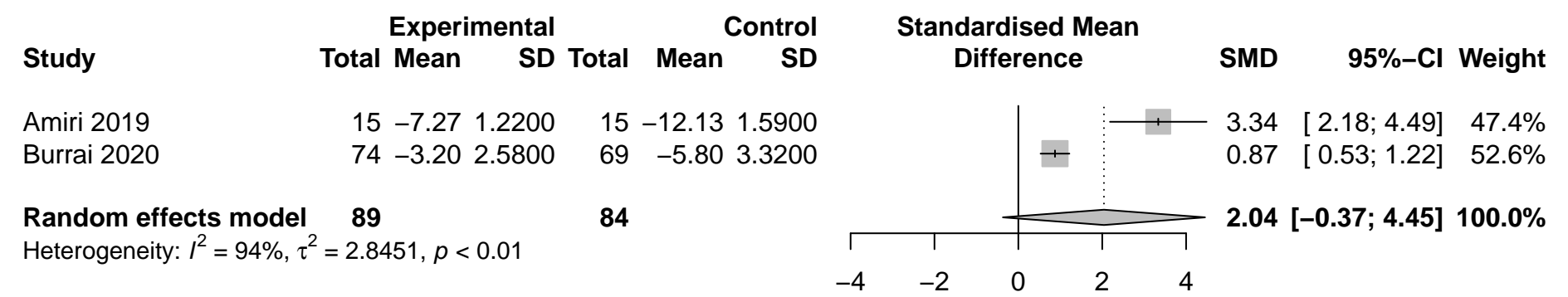

Supplement: Lassner et al. supplementary material 5 — Lassner et al. supplementary material [file S2056472424008263sup005.zip › analysis_2023.08.18/analysis/insomnia/insomnia_depression_endpoint.pdf]

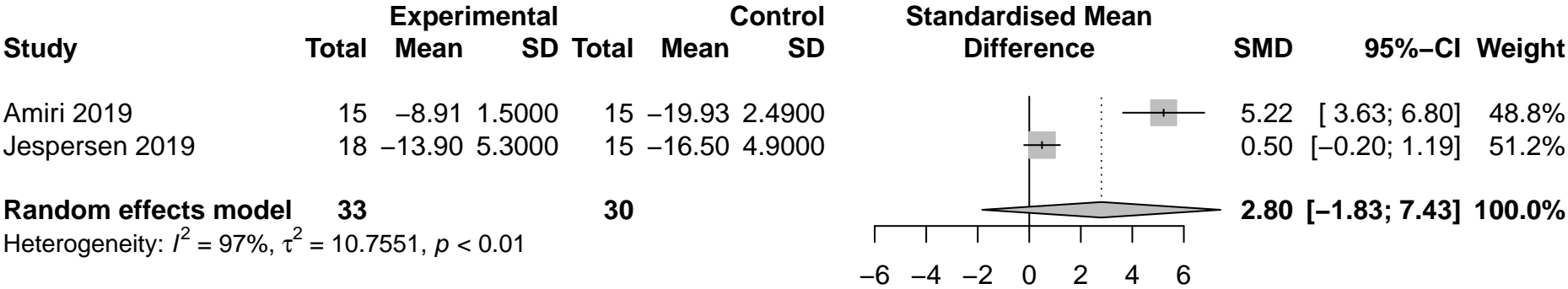

Supplement: Lassner et al. supplementary material 5 — Lassner et al. supplementary material [file S2056472424008263sup005.zip › analysis_2023.08.18/analysis/insomnia/insomnia_insomnia_severity_index_endpoint.pdf]

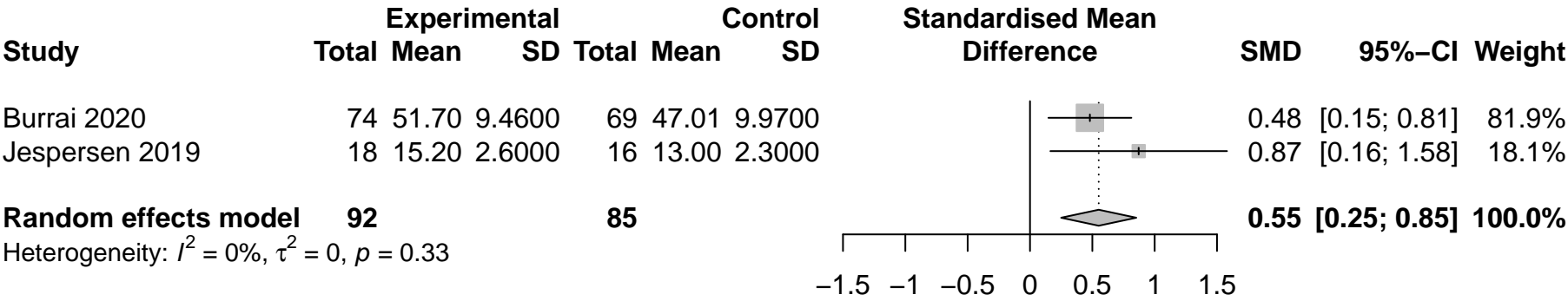

Supplement: Lassner et al. supplementary material 5 — Lassner et al. supplementary material [file S2056472424008263sup005.zip › analysis_2023.08.18/analysis/insomnia/insomnia_qol_endpoint.pdf]

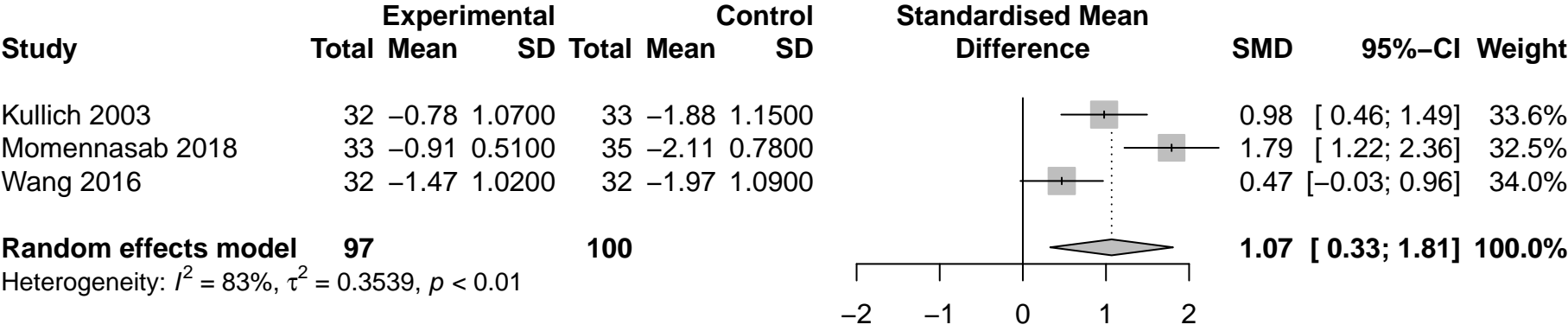

Supplement: Lassner et al. supplementary material 5 — Lassner et al. supplementary material [file S2056472424008263sup005.zip › analysis_2023.08.18/analysis/insomnia/insomnia_sleep efficiency_endpoint.pdf]

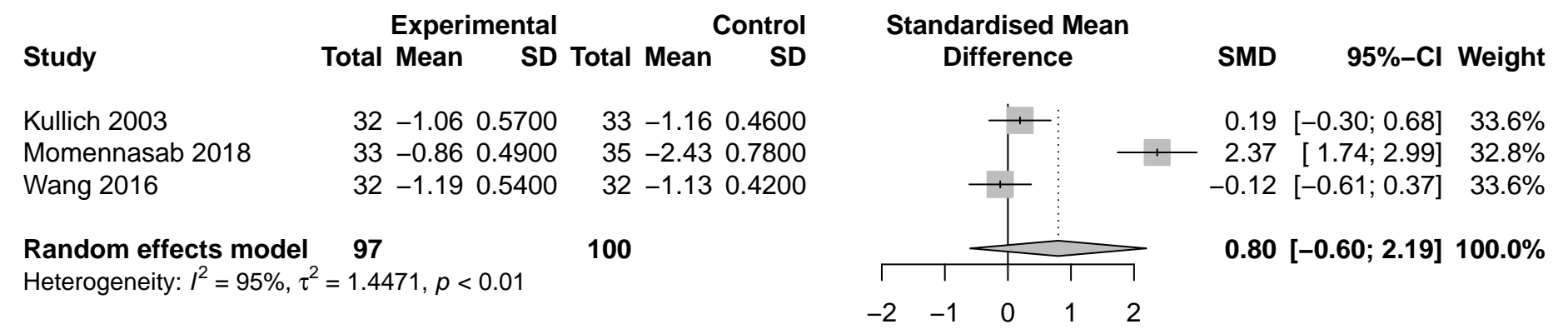

Supplement: Lassner et al. supplementary material 5 — Lassner et al. supplementary material [file S2056472424008263sup005.zip › analysis_2023.08.18/analysis/insomnia/insomnia_sleep interruption_endpoint.pdf]

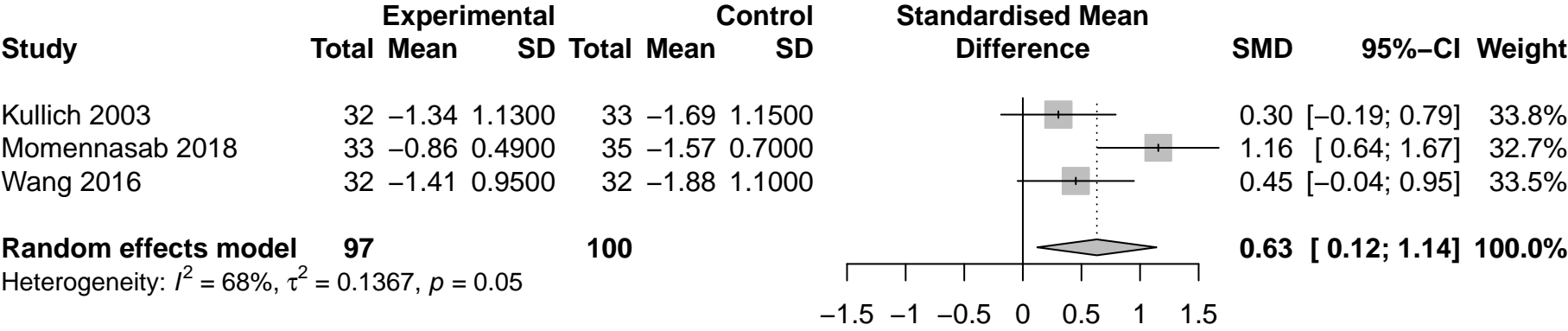

Supplement: Lassner et al. supplementary material 5 — Lassner et al. supplementary material [file S2056472424008263sup005.zip › analysis_2023.08.18/analysis/insomnia/insomnia_sleep onset latency_endpoint.pdf]

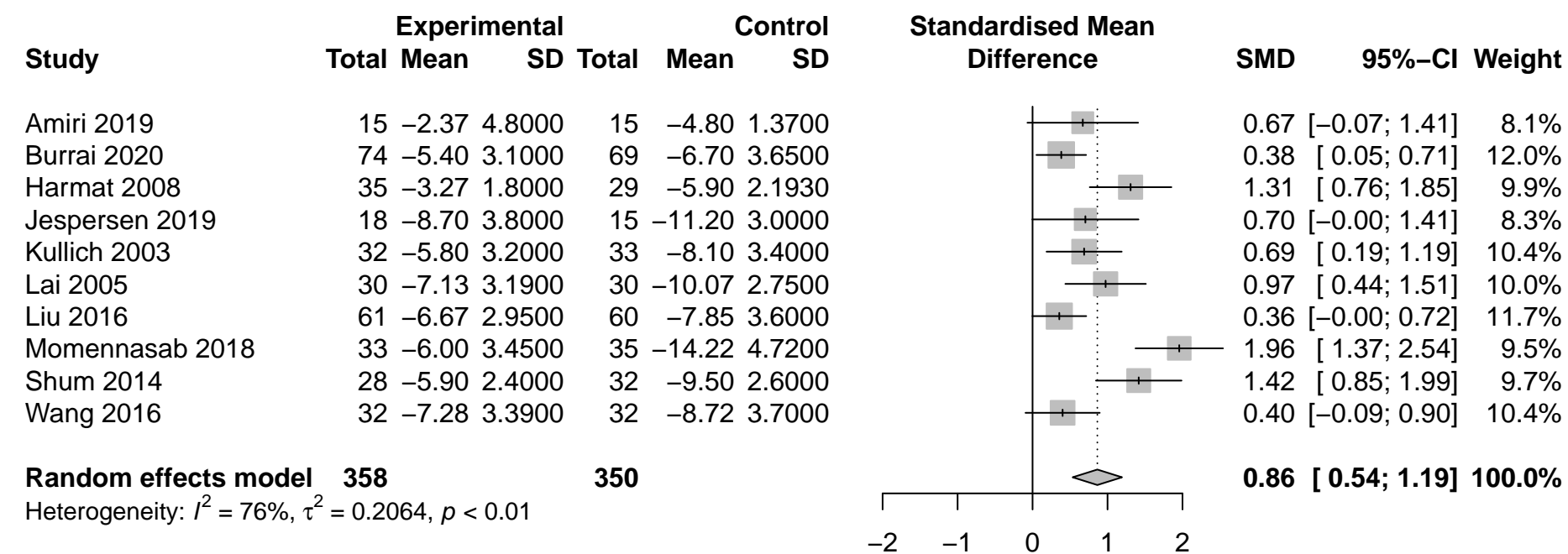

Supplement: Lassner et al. supplementary material 5 — Lassner et al. supplementary material [file S2056472424008263sup005.zip › analysis_2023.08.18/analysis/insomnia/insomnia_sleep_qol_endpoint.pdf]

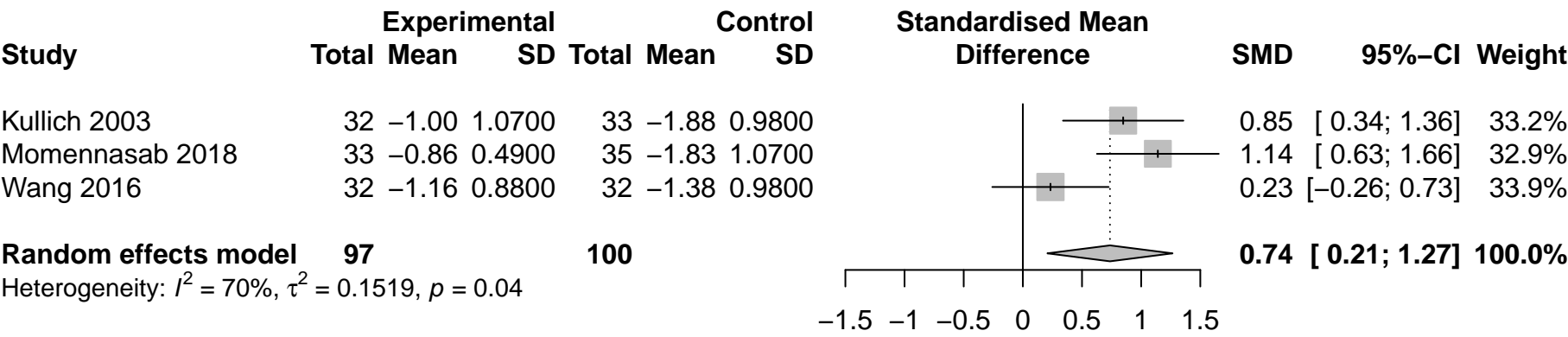

Supplement: Lassner et al. supplementary material 5 — Lassner et al. supplementary material [file S2056472424008263sup005.zip › analysis_2023.08.18/analysis/insomnia/insomnia_total sleep time_endpoint.pdf]

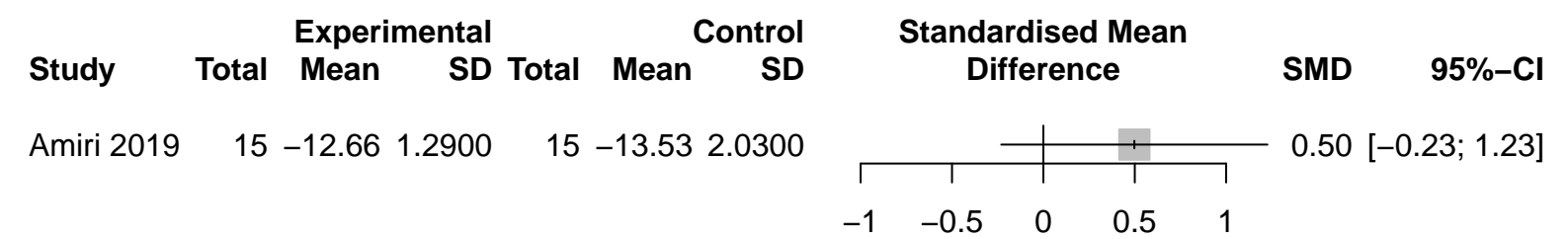

Supplement: Lassner et al. supplementary material 5 — Lassner et al. supplementary material [file S2056472424008263sup005.zip › analysis_2023.08.18/analysis/insomnia/primary_insomnia_anxiety_endpoint.pdf]

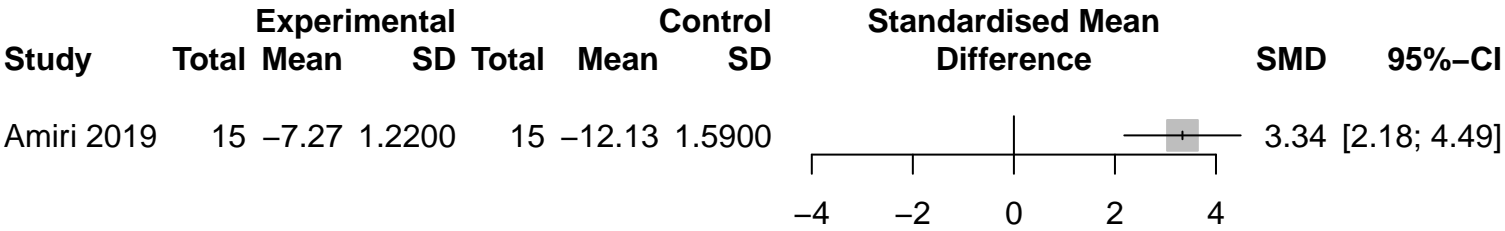

Supplement: Lassner et al. supplementary material 5 — Lassner et al. supplementary material [file S2056472424008263sup005.zip › analysis_2023.08.18/analysis/insomnia/primary_insomnia_depression_endpoint.pdf]

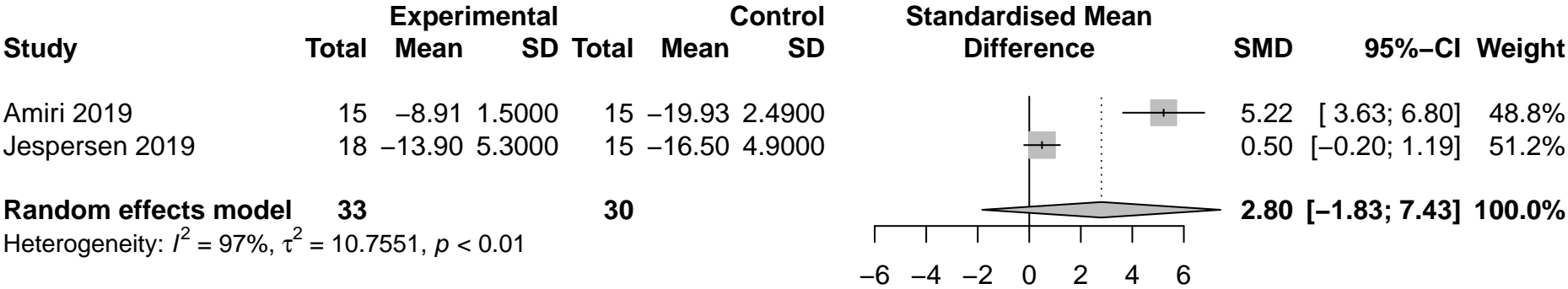

Supplement: Lassner et al. supplementary material 5 — Lassner et al. supplementary material [file S2056472424008263sup005.zip › analysis_2023.08.18/analysis/insomnia/primary_insomnia_insomnia_severity_index_endpoint.pdf]

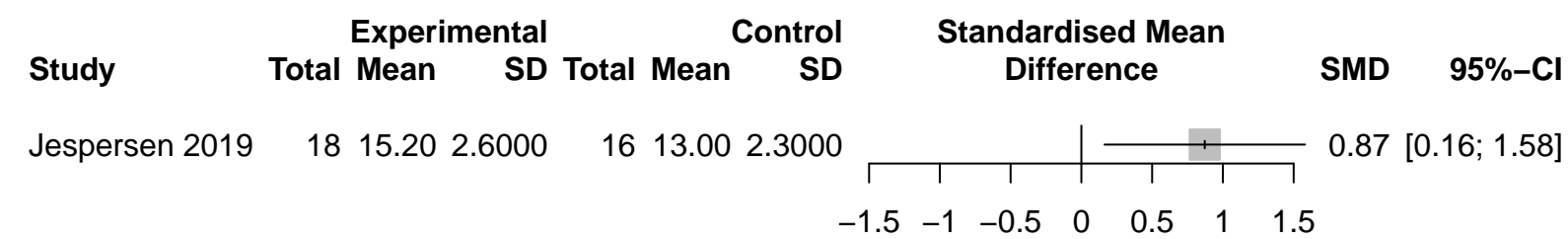

Supplement: Lassner et al. supplementary material 5 — Lassner et al. supplementary material [file S2056472424008263sup005.zip › analysis_2023.08.18/analysis/insomnia/primary_insomnia_qol_endpoint.pdf]

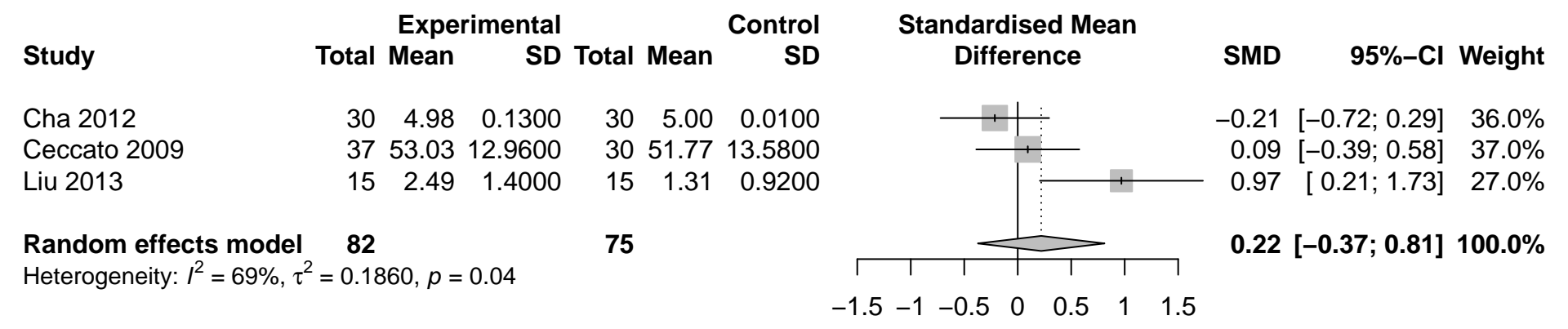

Supplement: Lassner et al. supplementary material 5 — Lassner et al. supplementary material [file S2056472424008263sup005.zip › analysis_2023.08.18/analysis/schizophrenia/schizophrenia_abstract-thinking_endpoint.pdf]

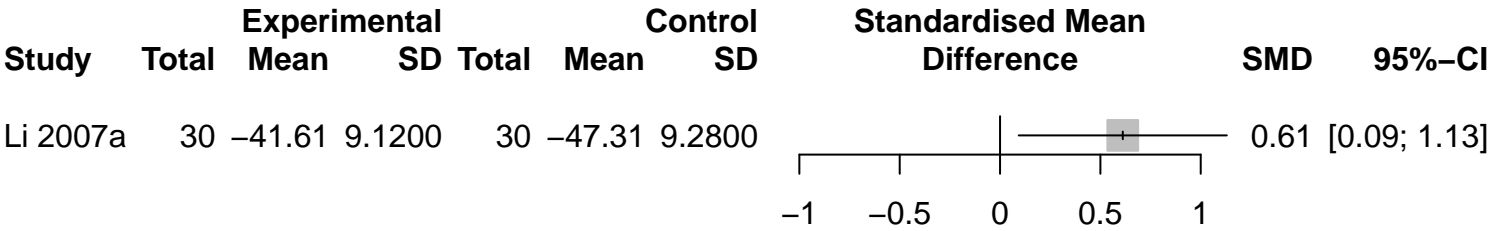

Supplement: Lassner et al. supplementary material 5 — Lassner et al. supplementary material [file S2056472424008263sup005.zip › analysis_2023.08.18/analysis/schizophrenia/schizophrenia_anxiety_endpoint.pdf]

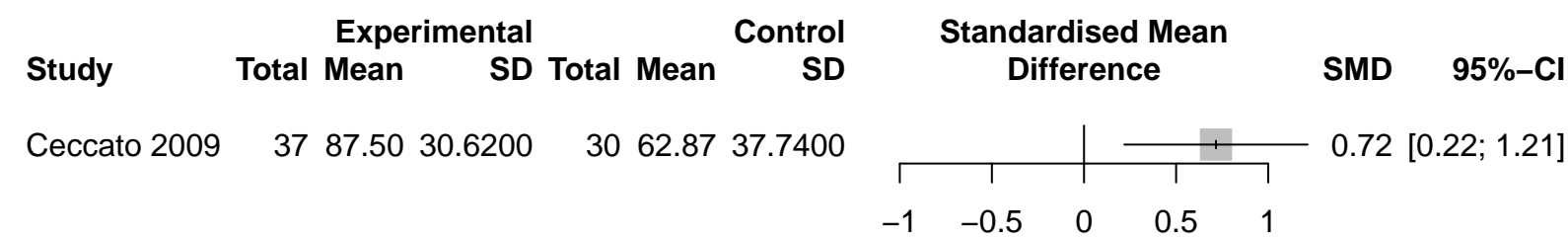

Supplement: Lassner et al. supplementary material 5 — Lassner et al. supplementary material [file S2056472424008263sup005.zip › analysis_2023.08.18/analysis/schizophrenia/schizophrenia_attention_endpoint.pdf]

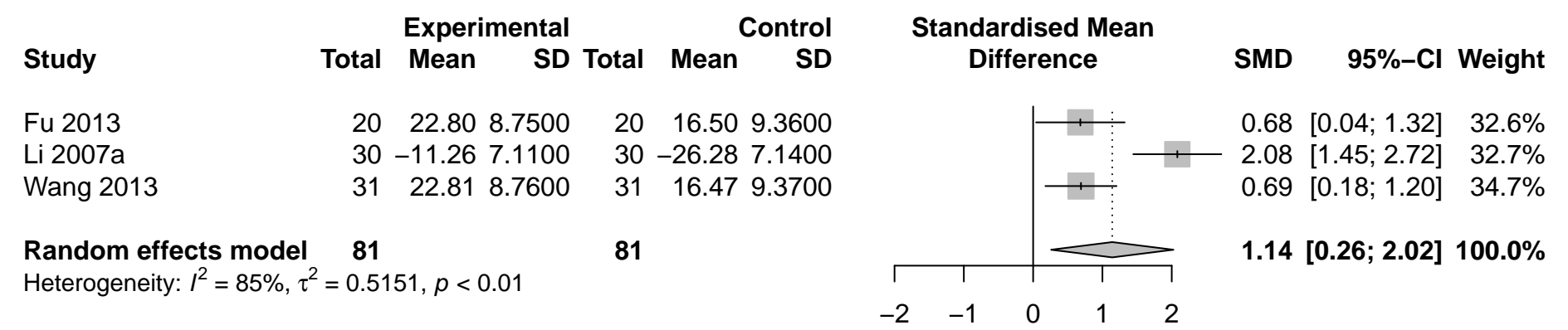

Supplement: Lassner et al. supplementary material 5 — Lassner et al. supplementary material [file S2056472424008263sup005.zip › analysis_2023.08.18/analysis/schizophrenia/schizophrenia_behavior_general_endpoint.pdf]

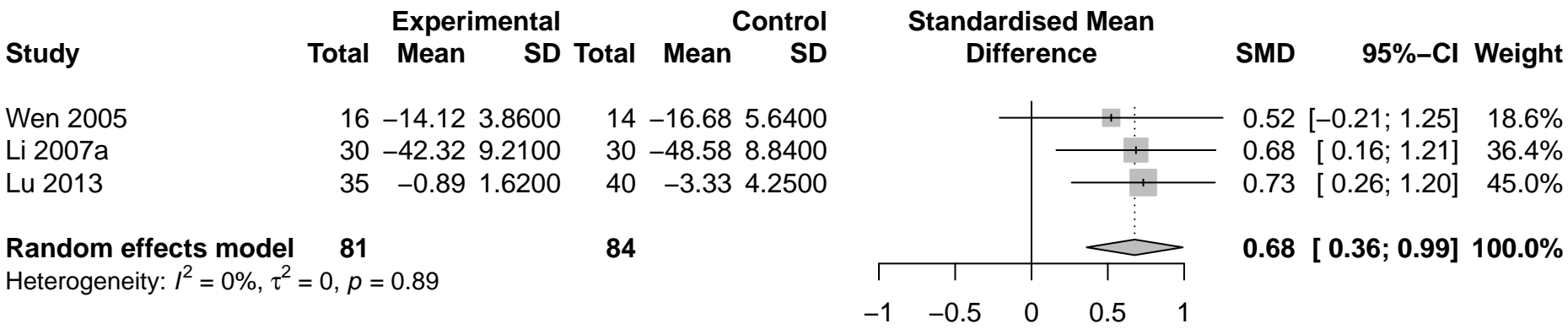

Supplement: Lassner et al. supplementary material 5 — Lassner et al. supplementary material [file S2056472424008263sup005.zip › analysis_2023.08.18/analysis/schizophrenia/schizophrenia_depression_endpoint.pdf]

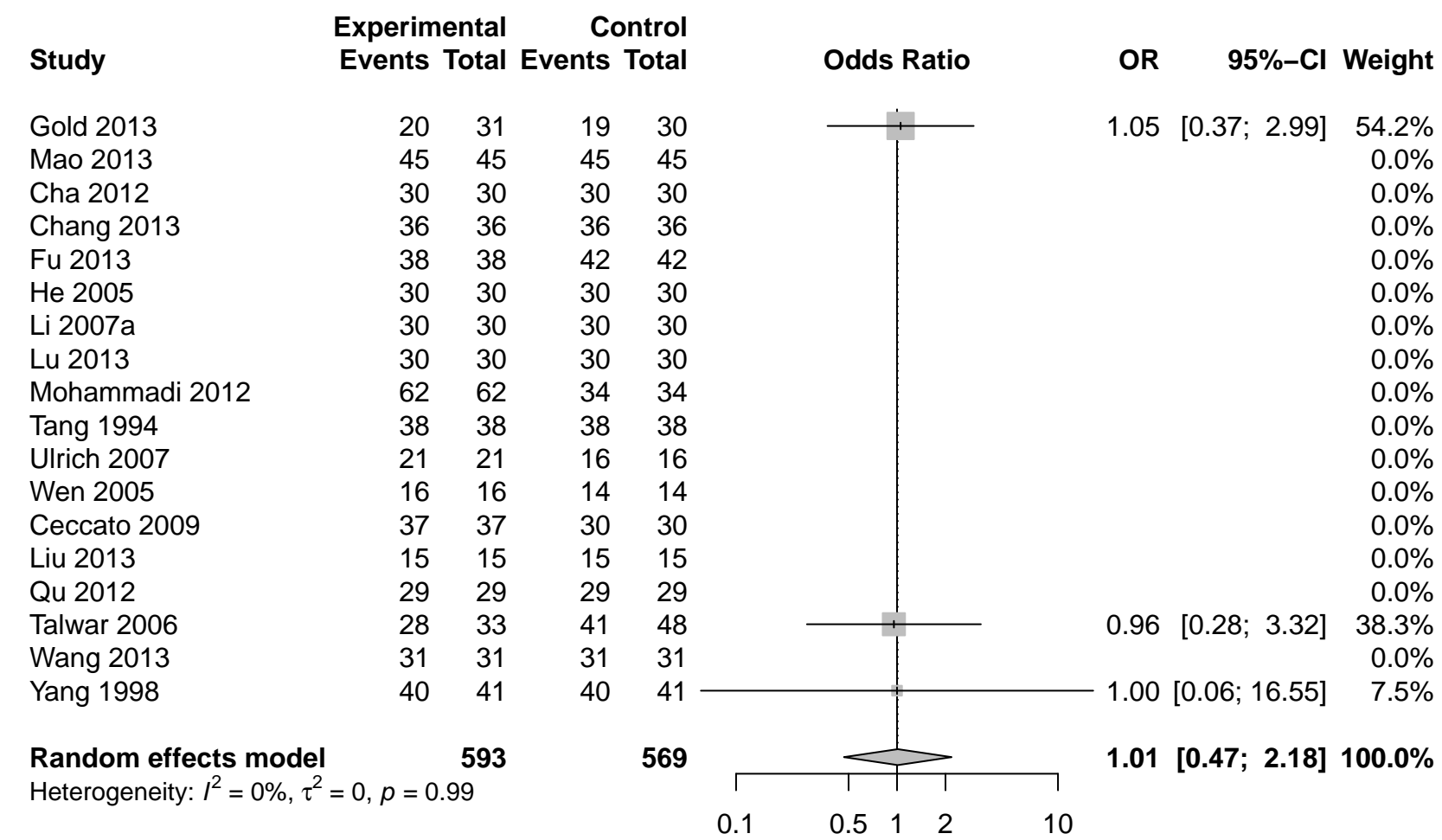

Supplement: Lassner et al. supplementary material 5 — Lassner et al. supplementary material [file S2056472424008263sup005.zip › analysis_2023.08.18/analysis/schizophrenia/schizophrenia_dropout_any_endpoint.pdf]

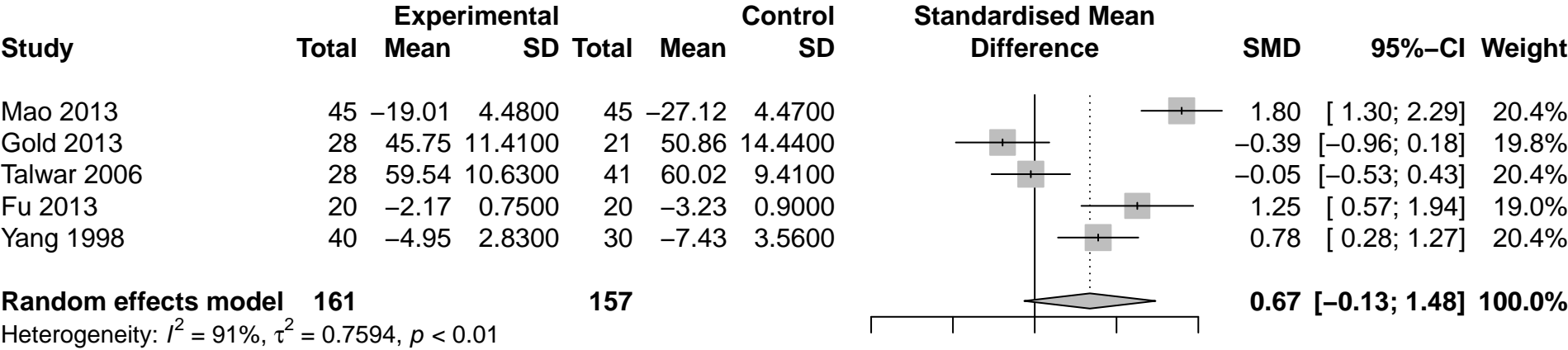

Supplement: Lassner et al. supplementary material 5 — Lassner et al. supplementary material [file S2056472424008263sup005.zip › analysis_2023.08.18/analysis/schizophrenia/schizophrenia_functioning_endpoint.pdf]

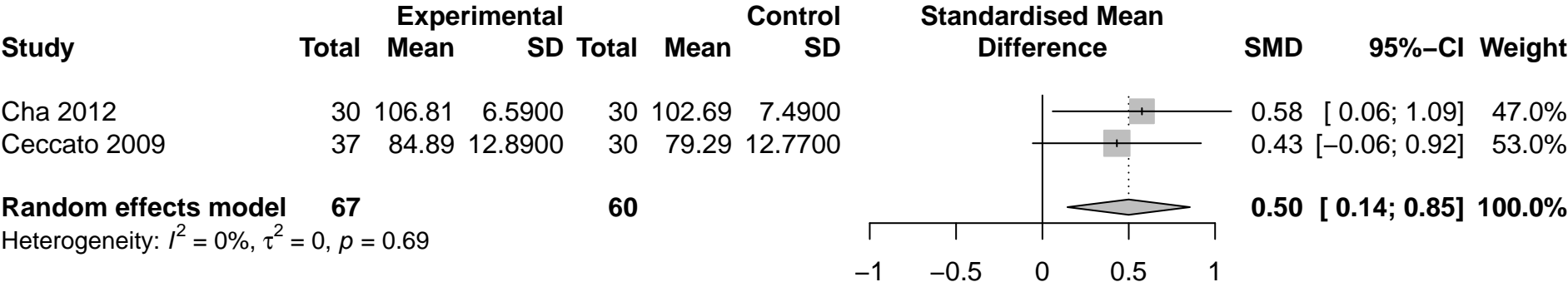

Supplement: Lassner et al. supplementary material 5 — Lassner et al. supplementary material [file S2056472424008263sup005.zip › analysis_2023.08.18/analysis/schizophrenia/schizophrenia_memory_endpoint.pdf]

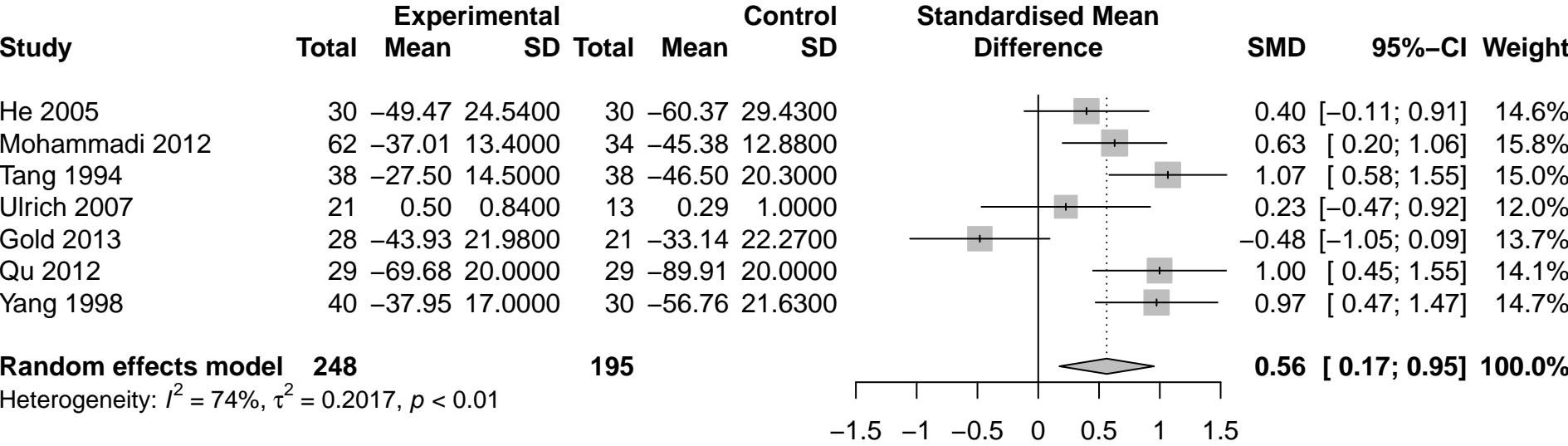

Supplement: Lassner et al. supplementary material 5 — Lassner et al. supplementary material [file S2056472424008263sup005.zip › analysis_2023.08.18/analysis/schizophrenia/schizophrenia_negative_endpoint.pdf]

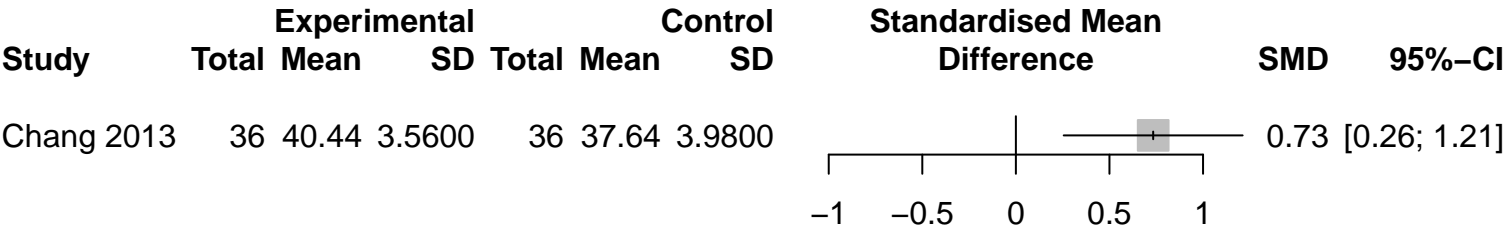

Supplement: Lassner et al. supplementary material 5 — Lassner et al. supplementary material [file S2056472424008263sup005.zip › analysis_2023.08.18/analysis/schizophrenia/schizophrenia_peceived_support_endpoint.pdf]

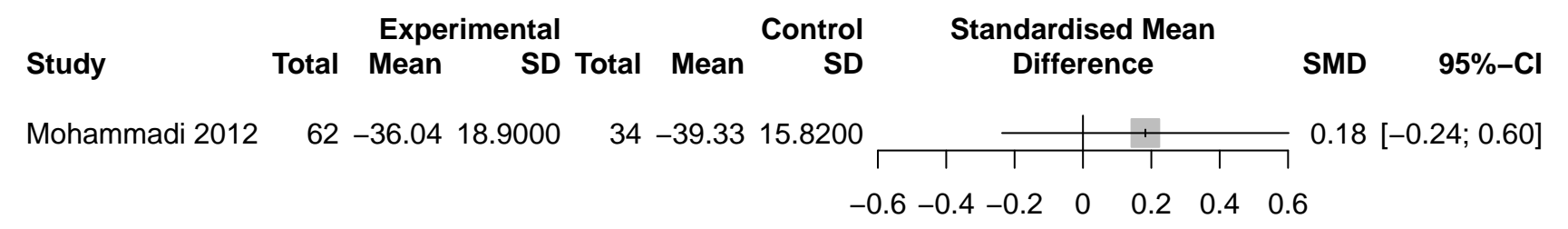

Supplement: Lassner et al. supplementary material 5 — Lassner et al. supplementary material [file S2056472424008263sup005.zip › analysis_2023.08.18/analysis/schizophrenia/schizophrenia_positive_endpoint.pdf]

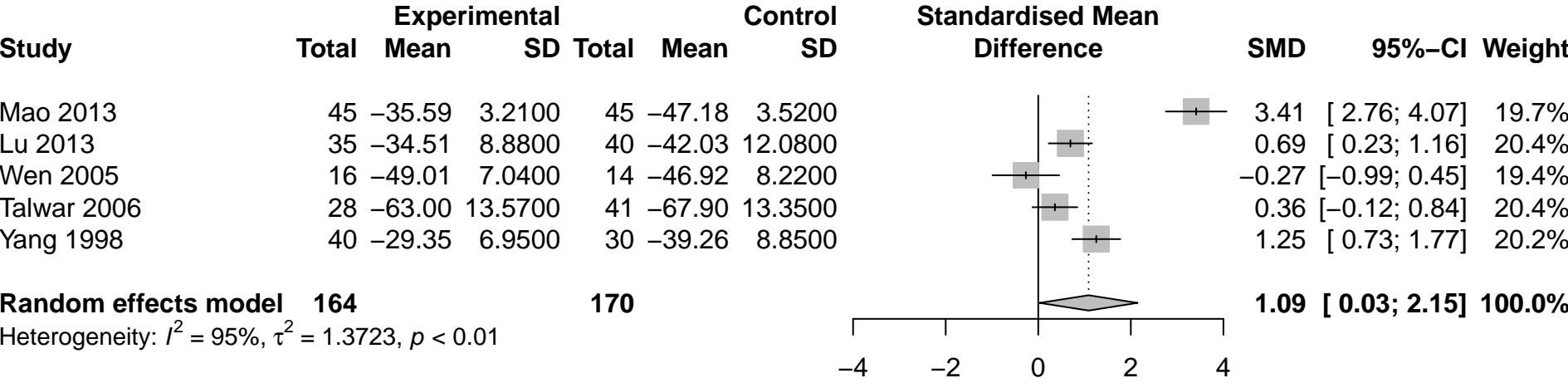

Supplement: Lassner et al. supplementary material 5 — Lassner et al. supplementary material [file S2056472424008263sup005.zip › analysis_2023.08.18/analysis/schizophrenia/schizophrenia_psychosis_symptoms_endpoint.pdf]

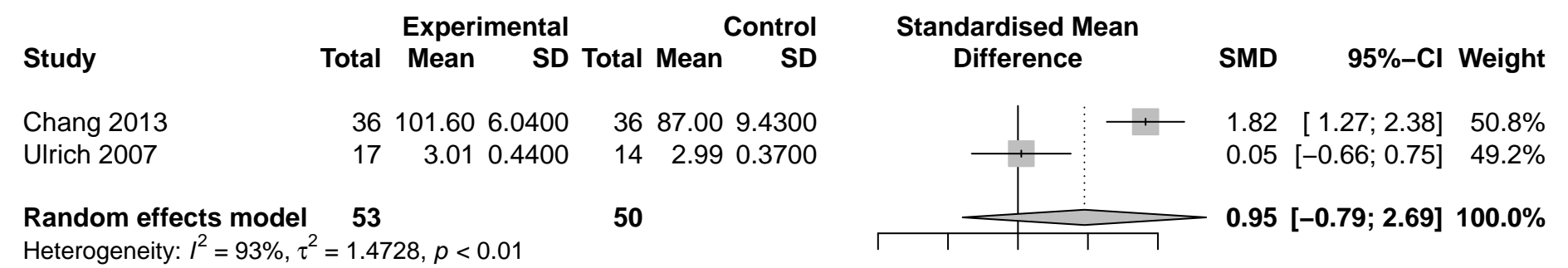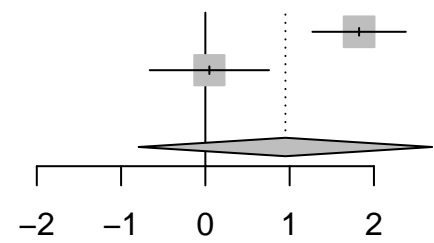

Supplement: Lassner et al. supplementary material 5 — Lassner et al. supplementary material [file S2056472424008263sup005.zip › analysis_2023.08.18/analysis/schizophrenia/schizophrenia_qol_endpoint.pdf]

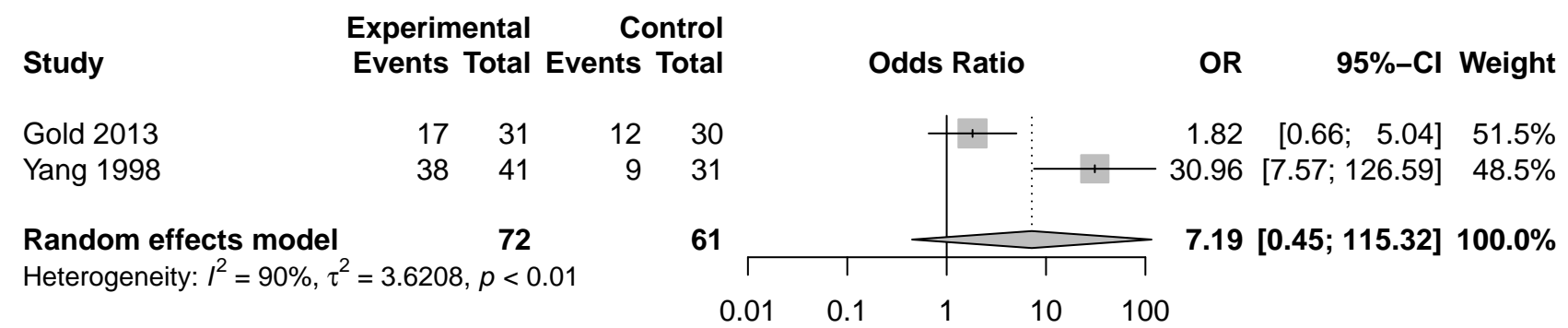

Supplement: Lassner et al. supplementary material 5 — Lassner et al. supplementary material [file S2056472424008263sup005.zip › analysis_2023.08.18/analysis/schizophrenia/schizophrenia_response_endpoint.pdf]

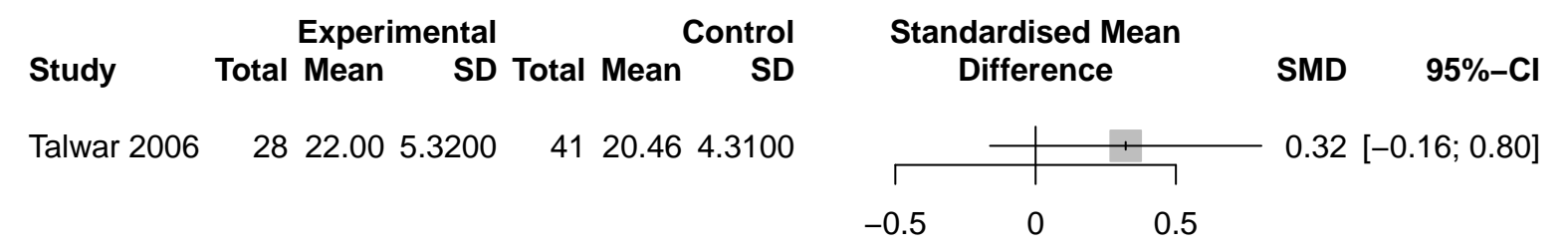

Supplement: Lassner et al. supplementary material 5 — Lassner et al. supplementary material [file S2056472424008263sup005.zip › analysis_2023.08.18/analysis/schizophrenia/schizophrenia_satisfcation_care_endpoint.pdf]

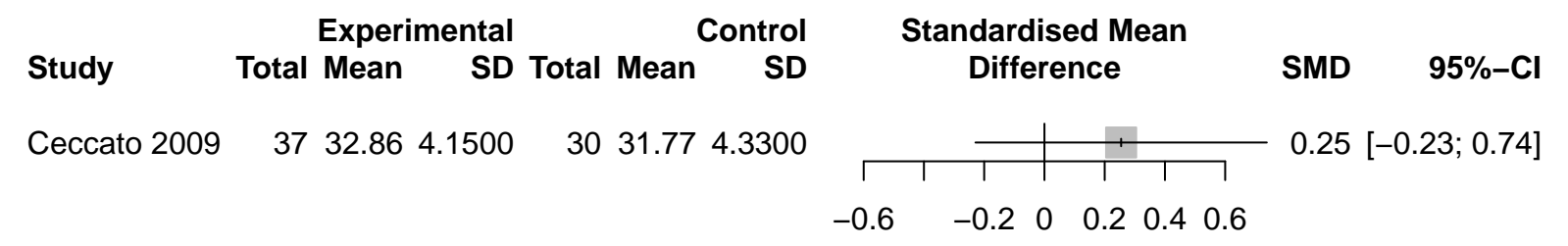

Supplement: Lassner et al. supplementary material 5 — Lassner et al. supplementary material [file S2056472424008263sup005.zip › analysis_2023.08.18/analysis/schizophrenia/schizophrenia_vigilance-attention_endpoint.pdf]

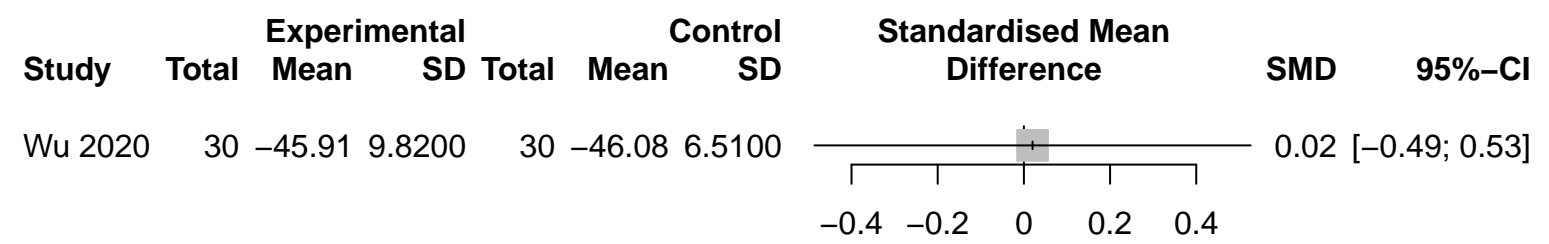

Supplement: Lassner et al. supplementary material 5 — Lassner et al. supplementary material [file S2056472424008263sup005.zip › analysis_2023.08.18/analysis/substance use/substance_use_anxiety_endpoint.pdf]

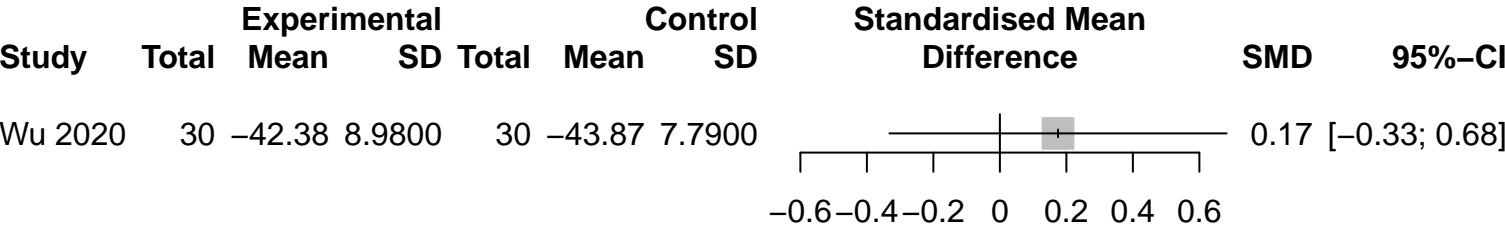

Supplement: Lassner et al. supplementary material 5 — Lassner et al. supplementary material [file S2056472424008263sup005.zip › analysis_2023.08.18/analysis/substance use/substance_use_anxiety_followup.pdf]

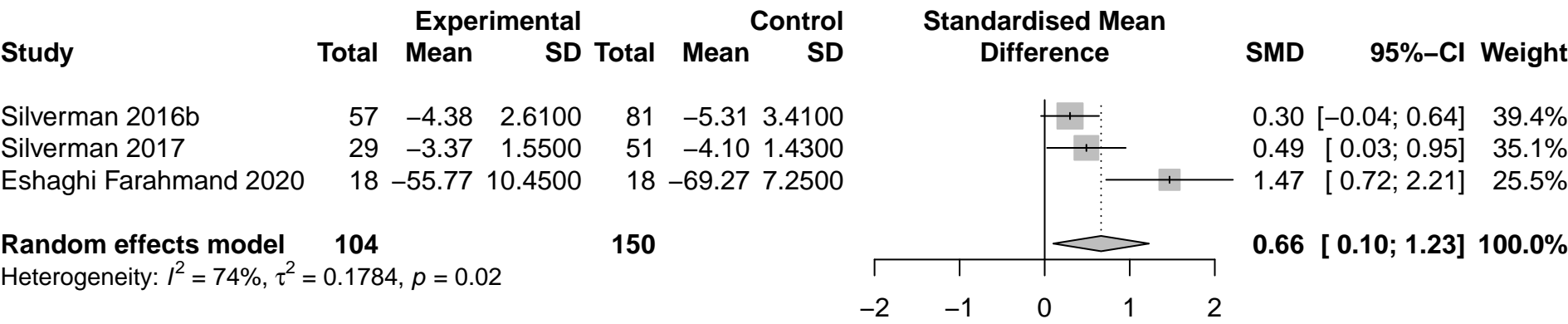

Supplement: Lassner et al. supplementary material 5 — Lassner et al. supplementary material [file S2056472424008263sup005.zip › analysis_2023.08.18/analysis/substance use/substance_use_craving_endpoint.pdf]

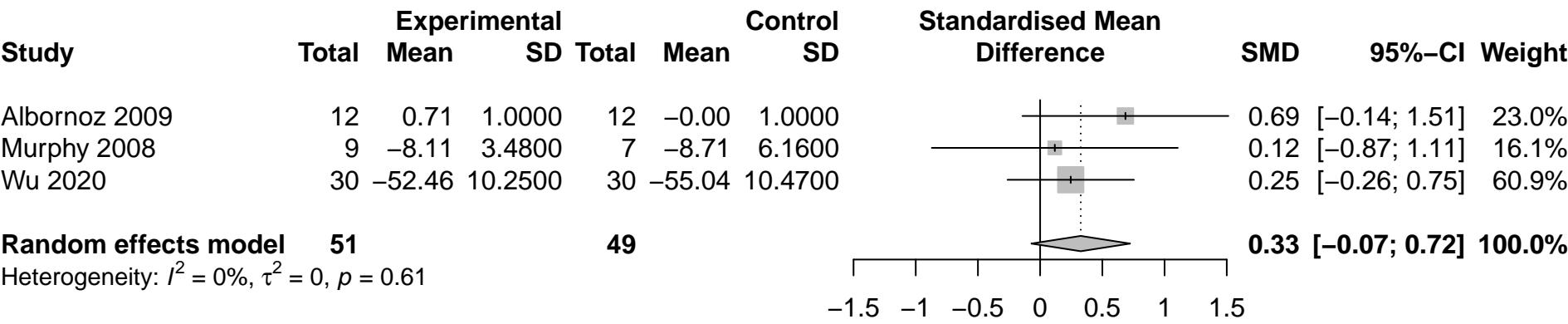

Supplement: Lassner et al. supplementary material 5 — Lassner et al. supplementary material [file S2056472424008263sup005.zip › analysis_2023.08.18/analysis/substance use/substance_use_depression_endpoint.pdf]

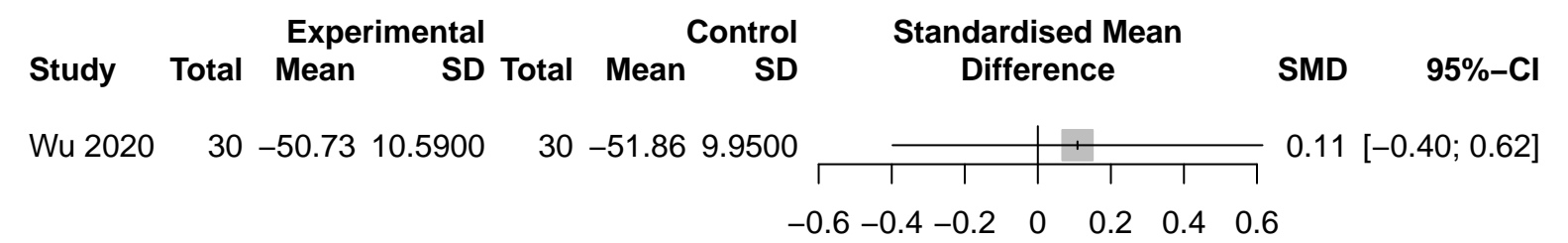

Supplement: Lassner et al. supplementary material 5 — Lassner et al. supplementary material [file S2056472424008263sup005.zip › analysis_2023.08.18/analysis/substance use/substance_use_depression_followup.pdf]

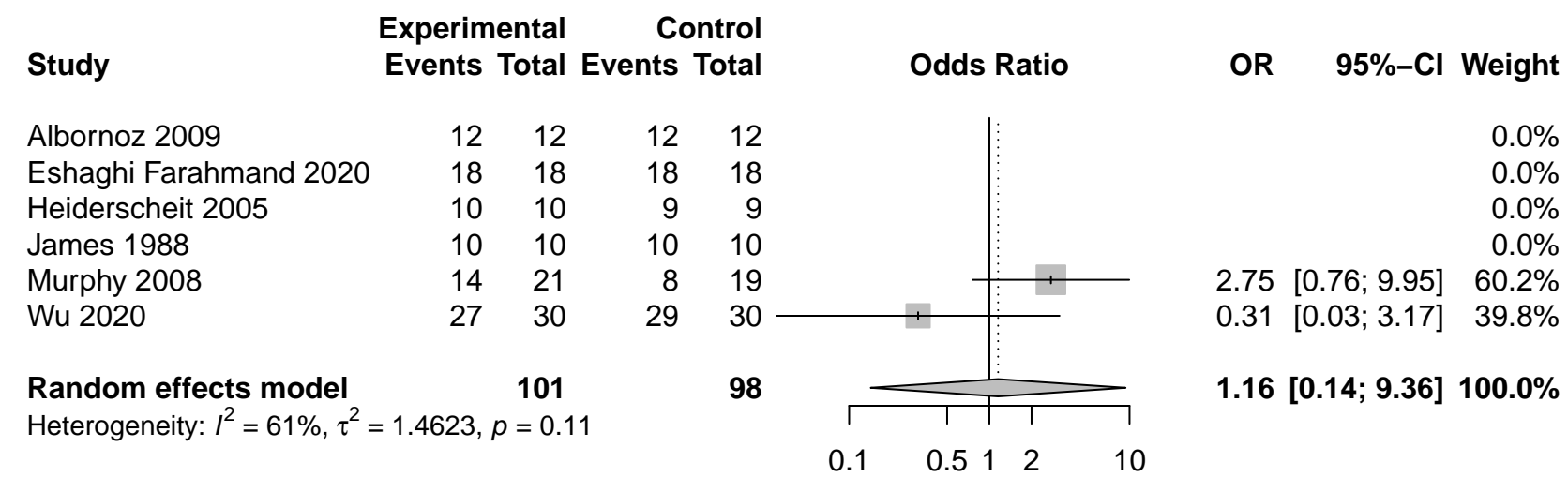

Supplement: Lassner et al. supplementary material 5 — Lassner et al. supplementary material [file S2056472424008263sup005.zip › analysis_2023.08.18/analysis/substance use/substance_use_dropout_any_endpoint.pdf]

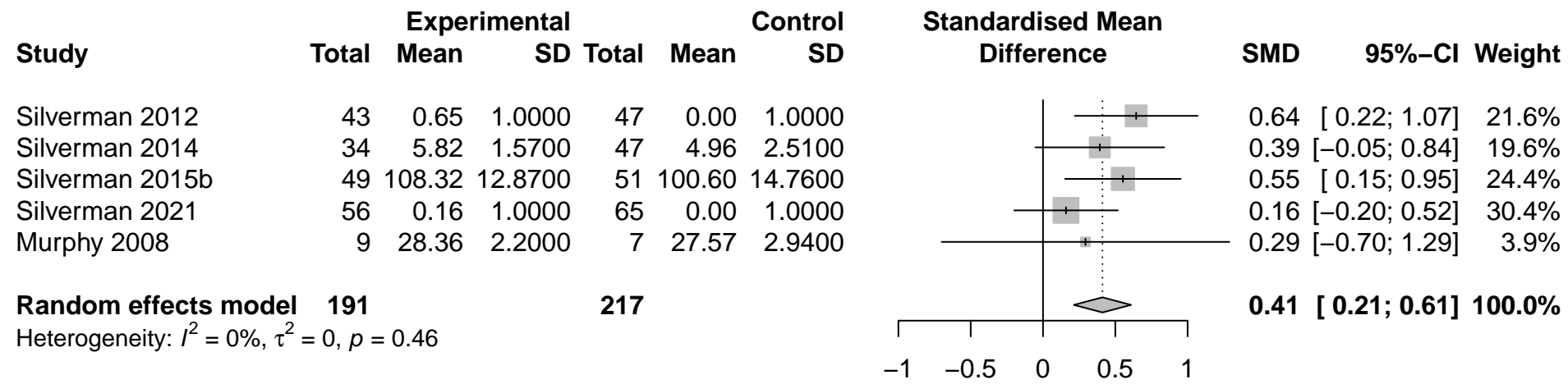

Supplement: Lassner et al. supplementary material 5 — Lassner et al. supplementary material [file S2056472424008263sup005.zip › analysis_2023.08.18/analysis/substance use/substance_use_motivation for treatment or change_endpoint.pdf]

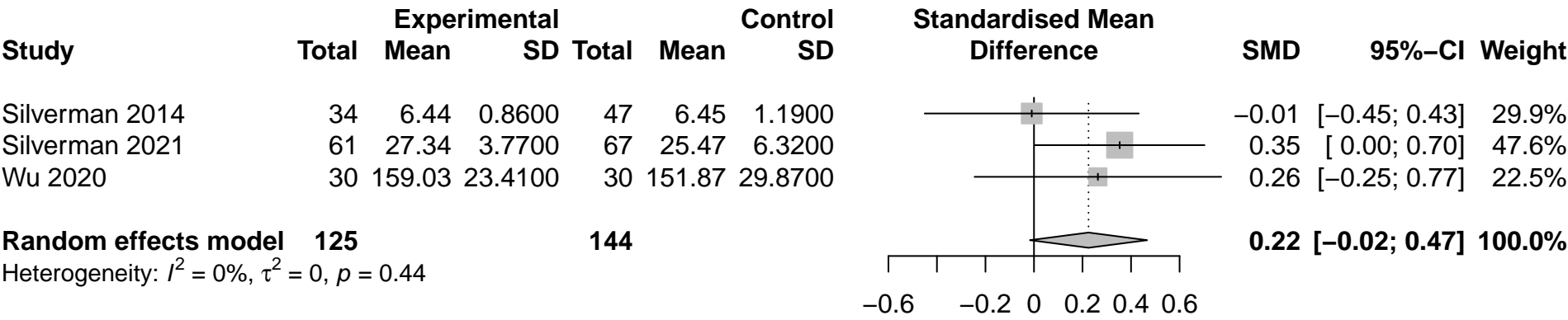

Supplement: Lassner et al. supplementary material 5 — Lassner et al. supplementary material [file S2056472424008263sup005.zip › analysis_2023.08.18/analysis/substance use/substance_use_motivation to stay sober or clean_endpoint.pdf]

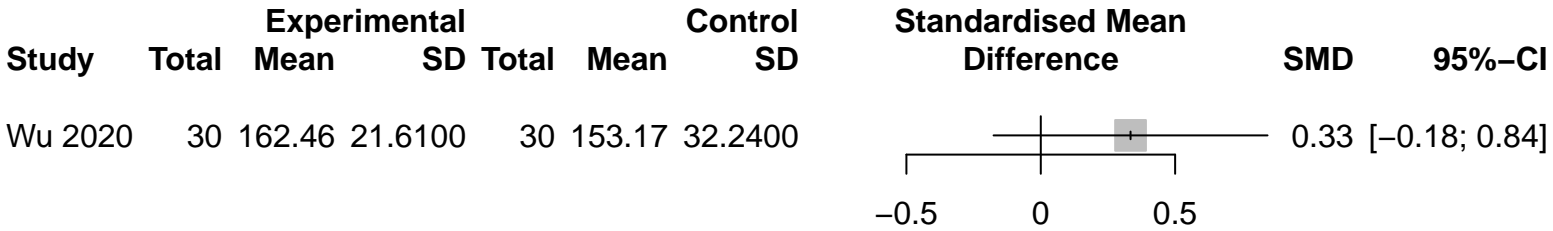

Supplement: Lassner et al. supplementary material 5 — Lassner et al. supplementary material [file S2056472424008263sup005.zip › analysis_2023.08.18/analysis/substance use/substance_use_motivation to stay sober or clean_followup.pdf]
