# Supplementary material for: Evidence for music therapy and music medicine in psychiatry: transdiagnostic meta-review of meta-analyses
Source: BJPsych Open. 2024 Dec 13;11(1):e4. doi: 10.1192/bjo.2024.826 (PMC11733488; doi:10.1192/bjo.2024.826)
Supplement: Lassner et al. supplementary material 8 — Lassner et al. supplementary material [file S2056472424008263sup008.docx]

**Appendix 8. References as presented in the Cochrane Reviews for forest plots & result table**

**DEPRESSION**

Albornoz 2011 {published data only} Albornoz Y. The effects of group improvisational music

therapy on depression in adolescents and adults with substance abuse: a randomised controlled trial. Nordic Journal of Music Therapy 2011;20(3):208-24.

Atiwannapat 2016 {published data only} Atiwannapat P, Thaipisuttikul P, Poopityastaporn P,

Katekaew W. Active versus receptive group music therapy for major depressive disorder - a pilot study. Complementary Therapies in Medicine 2016;26:141-5. [PUBMED: 27261995]

Chen 1992 {published data only} Chen X. Active music therapy for senile depression. Chinese

Journal of Neurology and Psychiatry 1992;25(4):208-10. [PUBMED: 1478135]

Erkkilä 2011 {published data only} Erkkilä J, Punkanen M, Fachner J, Ala-Ruona E, Pöntiö I,

Tervaniemi M, et al. Individual music therapy for depression: randomised controlled trial. British Journal of Psychiatry 2011;199:132-9. [PUBMED: 21474494]

Hanser 1994 {published data only} Hanser SB, Thompson LW. Effects of music therapy

strategy on depressed older adults. Journal of Gerontology 1994;49(6):265-9. [PUBMED: 7963281]

Hendricks 1999 {published data only} Hendricks CB, Robinson B, Bradley B, Davis K. Using

music techniques to treat adolescent depression. Journal of Humanistic Counseling, Education and Development 1999;38:39-46.

Hendricks 2001 {published data only} Hendricks CB. A study of the use of music therapy

techniques in a group for the treatment of adolescent depression. Dissertation Abstracts International 2001;62(2-A):472.

Radulovic 1996 {published data only} Radulovic R. The using of music therapy in treatment of

depressive disorders. Summary of Master Thesis. Belgrade: Faculty of Medicine University of Belgrade, 1996. [PUBMED: 1941727]

Zerhusen 1995 {published data only} Zerhusen JD, Boyle K, Wilson W. Out of the darkness:

group cognitive therapy for depressed elderly.Journal of Military Nursing Research 1995;1:28-32. [PUBMED: 1941727]

**SCHIZOPHRENIA**Ceccato 2009 {published data only}

Ceccato E. Music therapy in rehabilitation for people with schizophrenia. A multicenter, single blind, randomised controlled trial testing the effectiveness of the STAM protocol (Sound Training Attention and Memory) [Musicoterapia nella riabilitazione cognitiva del paziente schizofrenico. Studio multicentrico randomizzato e controllato in singolo cieco di applicazione del protocollo STAM (Sound Training Attention and Memory)]. Master's thesis 2010.

Ceccato E, Caneva P, Lamonaca D. Music therapy and cognitive rehabilitation in schizophrenic patients. Nordic Journal of Music Therapy 2006;15(2):111-20.

*  Ceccato E, Lamonaca D, Caneva PA, Gamba L, Poli R, Agrimi E. A multicentre study to test the effectiveness of the STAM (Sound Training Attention and Memory) protocol in the rehabilitation of patients with schizophrenia: a single blind, randomized control trial [Musicoterapia nella riabilitazione cognitiva del paziente schizofrenico. Studio multicentrico randomizzato controllatoin singolo cieco di applicazione del protocollo STAM (Sound Training Attention and Memory)]. Giornale Italiano di Psicopatologia 2009;15:395-400.

Cha 2012 {published data only} Cha ZQ, Li D, Wu QF, Zhang Y, Lu JB, M ZQ. The effect of

receptive music therapy on cognitive function of schizophrenia patients in remission [jie shou xing yin yue zhi liao dui jing shen fen lie huan jie qi ren zhi gong neng de ying xiang]. China Health Psychology 2012; Vol. 20, issue 07:1039-40. [CENTRAL: 20561]

Chang 2013 {published data only} Chang CH, Liu FF, Xu TH, Jiao TL. A research of music

therapy on subjective well-being and social support for patients with schizophrenia [yin yue zhi liao dui jing shen fen lie huan zhe zhu guan xing fu gan yu she hui zhi chi de xiang guan yan jiu]. National Medicine 2013; Vol. 25, issue 11:25-7.

Fu 2013 {published data only} Fu WJ, Zhang LF. The rehabilitation effect of improvisational

music therapy on the treatment of social disability in patients with schizophrenia in recovery period [ji xing yan zou shi yin yue zhi liao dui kang fu qi jing shen fen lie huan zhe she hui gong neng que sun de kang fu xiao ying]. Sichuan Mental Health 2013; Vol. 26, issue 03:215-8.

Gold 2013 {published and unpublished data}

* Gold C, Mossler K, Grocke D, Heldal TO, Tjemsland L, Aarre T, et al. Individual music therapy for mental health care clients with low therapy motivation: Multicentre randomised controlled trial. Psychotherapy and Psychosomatics 2013; Vol. 82, issue 5:319-31.

Gold C, Rolvsjord R, Aaro LE, Aarre T, Tjemsland L, Stige B. Resource-oriented music therapy for psychiatric patients with low therapy motivation: protocol for a randomised controlled trial. BMC Psychiatry 2005;5(39):1-8. [MEDLINE: 0; MEDLINE: 16259626]

Gold C, Tjemsland L, Tytlandsvik M, Heskestad S, Aarre T, Stige B. Resource-oriented music therapy for psychiatric patients with low therapy motivation: a randomised controlled trial. www.clinicaltrials.gov 2005.

He 2005 {published data only} He FR, Liu RK, Ma L. Influence of musical therapy on serum

PRL of patients with schizophrenia, type II [yin yue xin li zhi liao dui ‖xing jing shen fen lie huan zhe xue qing cui ru su shui ping de ying xiang]. Shandong Archives of Psychiatry 2005;18(2):78-9. [CAJ]

Li 2007a {published data only} Li YM, Ren X, Li CP, Li ZQ. The correct effect of language guided

music therapy on patients with schizophrenia [yu yan you dao shi yin yue liao fa dui jing shen fen lie huan zhe de xin li jiao zhi xiao guo]. International Nurses Journal 2007;26(9):917-8. [CHINESE: Academic Journals]

Liu 2013 {published data only} Liu SJ, Wu HL, Shi L. The impact of music therapy on cognitive

function for patients with chronic schizophrenia in recession [yin yue zhi liao dui jing shen fen lie zheng man xing shuai tui qi huan zhe ren zhi gong neng de ying xiang]. China Rehabilitation 2013; Vol. 28, issue 03:230-1.

Lu 2013 {published data only} Lu SF, Lo CH, Sung HC, Hsieh TC, Yu SC, Chang SC. Effects of

group music intervention on psychiatric symptoms and depression in patient with schizophrenia. Complementary therapies in medicine 2013; Vol. 21, issue 6:682-8.

Mao 2013 {published data only} Mao ZQ, Li D, Zhang GF, Cha ZQ, Rong JK. The effects of

music therapy on the rehabilitation of patients with chronic schizophrenia [yin yue zhi liao dui man xing jing shen fen lie zheng huan zhe de kang fu xiao guo guan cha]. China Journal of Health Psychology 2013; Vol. 21, issue 01:56-7.

Mohammadi 2012 {published data only} Mohammadi AZ, Minhas LS, Haidari M, Panah FM. A

study of the effects of music therapy on negative and positive symptoms in schizophrenic patients. German Journal of Psychiatry 2012; Vol. 15, issue 2:56-62.

Qu 2012 {published data only} Qu YN, Huang YF, Zhou ZX, Li LH. The rehabilitation care for

elderly patients with chronic schizophrenia in long-term hospitalization [chang qi zhu yuan man xing jing shen fen lie zheng huan zhe lao nian qi de kang fu hu li]. Guide of Chinese Medicine 2012; Vol. 10, issue 09:552-4.

Talwar 2006 {published and unpublished data}

Crawford M. A pilot randomised controlled trial to examine the effects of individual music therapy among inpatients with schizophrenia and schizophrenia like illness. National Research Register 2004; Vol. 2.

Gold C. Music therapy improves symptoms in adults hospitalised with schizophrenia. Evidence-Based Mental Health 2007;10(3):77. [CINAHL: 2009752767; MEDLINE: 17652559]

Maratos A. A pilot randomised controlled trial to examine the effects of individual music therapy among inpatients with schizophrenia and schizophrenia-like illnesses. Unpublished study protocol 2004.

Maratos A, Crawford M. Composing ourselves: What role might music therapy have in promoting recovery from acute schizophrenia?. London West Mental Health R&D Consortium's 9th Annual Conference. 2004.

Talwar N, Crawford M, Maratos A. A single blind randomised controlled trial of music therapy in patients with schizophrenia and related psychoses. Thematic Conference of the World Psychiatric Association on "Treatments in Psychiatry: An Update"; 2004 Nov 10-13; Florence, Italy. 2004.

* Talwar N, Crawford MJ, Maratos A, Nur U, McDermott O, Procter S. Music therapy for in-patients with schizophrenia: exploratory randomised controlled trial. British Journal of Psychiatry 2006;189:405-9. [AMED: 92246; MEDLINE: 17077429]

Tang 1994 {published data only (unpublished sought but not used)} Tang W, Yao X, Zheng Z.

Rehabilitative effect of music therapy for residual schizophrenia: A one-month randomised controlled trial in Shanghai. British Journal of Psychiatry 1994;165(suppl. 24):38-44.

Ulrich 2007 {unpublished data only}

Ulrich G. De toegevoegde waarde van groepsmuziektherapie bij schizofrene patiënten: Een gerandomiseer onderzoek [The added value of group music therapy with schizophrenic patients: A randomised study]. Heerlen, NL: Open Universiteit, 2005.

Ulrich G. [A randomised study of music therapy for schizophrenia: Study protocol]. Unpublished manuscript 2003.

*  Ulrich G, Houtmans T, Gold C. The additional therapeutic effect of group music therapy for schizophrenic patients: a randomized study. Acta Psychiatrica Scandinavica 2007;116(5):362-70. [MEDLINE: 0; MEDLINE: 17919155; PsycINFO]

Ulrich G, Houtmans T, Lechner L. [English Translation not available] [De toegevoegde waarde van groepsmuziektherapie bij schizofrene patiënten een gerandomiseerd onderzoek]. Dissertation submitted to Open Universiteit Nederland 2005.

Wang 2013 {published and unpublished data} Wang S. The effects of music therapy on

improving social function in patients with chronic schizophrenia [man xing jing shen fen lie huan zhe fu yi yin yue zhi liao dui qi she hui gong neng de gai shan]. The National Medicine 2013; Vol. 25, issue 13:51-2.

Wen 2005 {published data only} Wen SR, Cao GY, Zhou HS. The effect of music therapy on the

depressive position of patients with schizophrenia [yin yue zhi liao dui jing shen fen lie huan zhe de yi yu zhuang tai de ying xiang]. Chinese Journal of Clinical Rehabilitation 2005;9(8):195.

Yang 1998 {published data only (unpublished sought but not used)} Yang WY, Li Z, Weng YZ,

Zhang HY, Ma B. Psychosocial rehabilitation effects of music therapy in chronic schizophrenia. Hong Kong Journal of Psychiatry 1998;8(1):38-40.

**AUTISM**

Arezina 2011 {published and unpublished data} Arezina CH. The Effect of Interactive Music

Therapy on Joint Attention Skills in Preschool Children with Autism Spectrum Disorder [Master's thesis]. Lawrence (KS): University of Kansas, 2011.

Bharathi 2019 {published and unpublished data} Bharathi G, Venugopal A, Vellingiri B. Music

therapy as a therapeutic tool in improving the social skills of autistic children. Egyptian Journal of Neurology, Psychiatry and Neurosurgery 2019;55:44. [DOI: 10.1186/s41983-019-0091-x]

Bieleninik 2017 {published and unpublished data}

* Bieleninik Ł, Geretsegger M, Mössler K, Assmus J, Thompson G, Gattino G, et al.Effects of improvisational music therapy vs enhanced standard care on symptom severity among children with autism spectrum disorder: the TIMEA randomized clinical trial. JAMA 2017;318(6):525-35. [DOI: 10.1001/jama.2017.9478] [PMCID: PMC5817481] [PMID: 28787504]

Crawford MJ, Gold C, Odell-Miller H, Thana L, Faber S, Assmus J, et al. International multicentre randomised controlled trial of improvisational music therapy for children with autism spectrum disorder: TIME-A study. Health Technology Assessment 2017;21(59):1-40. [DOI: 10.3310/hta21590] [PMCID: PMC5672498] [PMID: 29061222]

Geretsegger M, Holck U, Gold C. Randomised controlled trial of improvisational music therapy's effectiveness for children with autism spectrum disorders (TIME-A): study protocol.BMC Pediatrics 2012;12:2. [DOI: 10.1186/1471-2431-12-2] [PMCID: PMC3280156] [PMID: 22221670]

ISRCTN78923965.Trial of improvisational music therapy's effectiveness for children with autism [Randomised controlled trial of improvisational music therapy's effectiveness for children with autism spectrum disorders (TIME-A)]. doi.org/10.1186/ISRCTN78923965 (first received 18 June 2011).

Brownell 2002 {published and unpublished data} Brownell MD. Musically adapted social

stories to modify behaviors in students with autism: four case studies. Journal of Music Therapy 2002;39(2):117-44. [DOI: 10.1093/jmt/39.2.117] [PMID: 12213082]

Buday 1995 {published data only} Buday EM. The effects of signed and spoken words taught

with music on sign and speech imitation by children with autism. Journal of Music Therapy 1995;32(3):189-202. [DOI: 10.1093/ jmt/32.3.189]

Chen 2010 {published data only} Chen L, Tao HM, Li H, Shao Z, Yao L, Liao LJ. Empirical study

of clinical effect of music therapy on children with autism [音乐治疗干预自闭症儿童临床效果的实证研究]. Chongqing Medicine 2010;39(18):2481-2, 2485. [DOI: 10.3969/ j.issn.1671-8348.2010.18.042]

Chen 2013 {published data only} Chen L, Zhang YR, Shao Z, Zhao P, Liao LJ. Observation on the

curative effect of music therapy with social stories as the main content in intervention of children with autism [以社交故事为主要内容的音乐疗法干预孤 独症儿童的疗效观察]. Chinese Journal of Physical Medicine and Rehabilitation 2013;35(7):575-8. [DOI: 10.3760/ cma.j.issn.0254-1424.2013.07.019]

Farmer 2003 {published data only} Farmer KJ. The Effect of Music vs Nonmusic Paired with

Gestures on Spontaneous Verbal and Nonverbal Communication Skills of Children with Autism Between the Ages 1-5 [Master's thesis]. Tallahassee (FL): Florida State University, 2003.

Gattino 2011 {published data only} Gattino GS, Riesgo RdS, Longo D, Leite JC, Faccini LS.

Effects of relational music therapy on communication of children with autism: a randomized controlled study. Nordic Journal of Music Therapy 2011;20(2):142-54. [DOI: 10.1080/08098131.2011.566933]

Ghasemtabar 2015 {published data only} Ghasemtabar SN,  Hosseini M, Fayyaz I, Arab S,

Naghashian H, Poudineh Z. Music therapy: an effective approach in improving social skills of children with autism. Advanced Biomedical Research 2015;4:157. [DOI: 10.4103/2277-9175.161584] [PMCID: PMC4550953] [PMID: 26380242]

Huang 2015 {published data only} Huang LY. Rehabilitation applications of music therapy in

children with autism [音乐治疗在自闭症儿童康复中的应用]. China Continuing Medical Education 2015;7(18):153-4.

Kim 2008 {published and unpublished data}

Kim J, Wigram T, Gold C. Emotional, motivational and interpersonal responsiveness of children with autism in improvisational music therapy. Autism 2009;13(4):389-409. [DOI: 10.1177/1362361309105660] [PMID: 19535468]

*  Kim J, Wigram T, Gold C. The effects of improvisational music therapy on joint attention behaviors in autistic children: a randomized controlled study. Journal of Autism and Developmental Disorders 2008;38(9):1758-66. [DOI: 10.1007/ s10803-008-0566-6] [PMID: 18592368]

Kim J. The Effects of Improvisational Music Therapy on Joint Attention Behaviours in Children with Autistic Spectrum Disorder [PhD thesis]. Aalborg, Denmark: Aalborg University, 2006.

LaGasse 2014 {published and unpublished data} LaGasse AB. Effects of a music therapy group

intervention on enhancing social skills in children with autism. Journal of Music Therapy 2014;51(3):250-75. [DOI: 10.1093/jmt/thu012] [PMID: 25053766]

Lim 2010 {published data only}

*  Lim HA. Effect of "developmental speech and language training through music" on speech production in children with autism spectrum disorders. Journal of Music Therapy 2010;47(1):2-26. [DOI: 10.1093/jmt/47.1.2] [PMID: 20635521]

Lim HA. The Effect of "Developmental Speech-Language Training through Music" on Speech Production in Children with Autism Spectrum Disorders [PhD thesis]. Coral Gables (FL): University of Miami, 2007. [WEB PAGE: citeseerx.ist.psu.edu/viewdoc/ download?doi=10.1.1.462.5692&rep=rep1&type=pdf]

Lim 2011 {published data only} Lim HA, Draper E. The effects of music therapy incorporated

with applied behavior analysis verbal behavior approach for children with autism spectrum disorders. Journal of Music Therapy 2011;48(4):532-50. [DOI: 10.1093/jmt/48.4.532] [PMID: 22506303]

Mateos-Moreno 2013 {published data only} Mateos-Moreno D, Atencia-Doña L. Effect of a

combined dance/ movement and music therapy on young adults diagnosed with severe autism. Arts in Psychotherapy 2013;40(5):465-72. [DOI: 10.1016/j.aip.2013.09.004]

Moon 2010 {published data only}

Moon JY. Effects of therapeutic music play based on theory of mind for children with Asperger syndrome's self-cognition, self-efficacy, and self- esteem[마음이론에 근거한 음악극이 아 스퍼거 증후군 아동의자기인식과 자기효능감 및 자아존중감에 미치는 영향]. Emotional and Behavioral Disorders Research 2010;26(2):197-222. [WEB PAGE: www.dbpia.co.kr/Journal/ articleDetail?nodeId=NODE08783523]

Porter 2017 {published and unpublished data}

McConnell T. Data request for Cochrane review about "Music Therapy for ASD" [personal communication]. Email to: C Gold 23 November 2020.

*  Porter S,  McConnell T, McLaughlin K, Lynn F, Cardwell C, Braiden H-J, et al. Music therapy for children and adolescents with behavioural and emotional problems: a randomised controlled trial. Journal of Child Psychology and Psychiatry 2017;58(5):586-94. [DOI: 10.1111/jcpp.12656] [PMID: 27786359]

Porter S, Holmes V, McLaughlin K, Lynn F, Cardwell C, Braiden HJ, et al. Music in mind, a randomized controlled trial of music therapy for young people with behavioural and emotional problems: study protocol. Journal of Advanced Nursing 2012;68(10):2349-58. [DOI: 10.1111/j.1365-2648.2011.05936.x] [PMID: 22235808]

Rabeyron 2020 {published and unpublished data} *  Rabeyron T,  Robledo Del Canto J-

P,  Carasco E,  Bisson V,  Bodeau N,  Vrait F-X, et al.A randomized controlled trial of 25 sessions comparing music therapy and music listening for children with autism spectrum disorder. Psychiatry Research 2020;293:113377. [DOI: 10.1016/j.psychres.2020.113377] [PMID: 32798927]

Sa 2020 {published data only} Sa V. The Effect of Music Attention Control Training (MACT) for

Pre-Adolescents with Autism Spectrum Disorder [Master's thesis]. Stockton (CA): University of the Pacific, 2020.

Schwartzberg 2013 {published and unpublished data} Schwartzberg ET, Silverman MJ. Effects

of music-based social stories on comprehension and generalization of social skills in children with autism spectrum disorders: a randomized effectiveness study. Arts in Psychotherapy 2013;40(3):331-7. [DOI: 10.1016/j.aip.2013.06.001]

Schwartzberg 2016 {published and unpublished data} Schwartzberg ET,  Silverman MJ. Effects

of a music-based short story on short- and long-term reading comprehension of individuals with autism spectrum disorder: a cluster randomized study. Arts in Psychotherapy 2016;48:54-61. [DOI: 10.1016/j.aip.2016.01.001]

Sharda 2018 {published data only}26821793 Latif N, Di Francesco C, Custo-Blanch M, Hyde K,

Sharda M, Nadig A.Joint engagement and movement: active ingredients of a music-based intervention with school-age children with autism. NeuroRehabilitation 2021;48(2):167-85. [DOI: 10.3233/ NRE-208012] [PMID: 33664155]

*  Sharda M,  Tuerk C, Chowdhury R, Jamey K, Foster N,  Custo-Blanch M, et al. Music improves social communication and auditory-motor connectivity in children with autism. Translational Psychiatry 2018;8(1):231. [DOI: 10.1038/ s41398-018-0287-3] [PMCID: PMC6199253] [PMID: PMC6199253]

Thomas 2003 {unpublished data only} Thomas AF, Hunter BC. The effect of music therapy on

communication skills of children with autism, ages 2-3: a pilot study. In: Music Therapy: The Key to Health and Wellness. Proceedings of the FiPh American Music Therapy Association Conference; 2003 18-23 Nov; Minneapolis (MN). 2003:43.

Thompson 2014 {published and unpublished data} Thompson G. Making a Connection:

Randomised Controlled Trial of Family Centred Music Therapy for Young Children with Autism Spectrum Disorder [PhD thesis]. Melbourne (VIC): University of Melbourne, 2012. [URI: http:// hdl.handle.net/11343/37719]

*  Thompson GA,  McFerran KS, Gold C. Family-centred music therapy to promote social engagement in young children with severe autism spectrum disorder: a randomized controlled study. Child: Care, Health & Development 2014;40(6):840-52. [DOI: 10.1111/cch.12121] [PMID: 24261547]

Yurteri 2019 {published data only} Yurteri N, Akdemir M. The effect of music therapy on

autistic symptoms and quality of life in children with autism spectrum disorder [Otizm spektrum bozukluğu olan çocuklarda müzik terapinin otizm belirtileri ve yaşam kalitesine etkisi]. Anatolian Journal of Psychiatry [Anadolu Psikiyatri Dergisi] 2019;20(4):436-41. [DOI: 10.5455/apd.12505]

**SUBSTANCE-USE DISORDER**

Albornoz 2009 {published data only} Albornoz Y. The effects of group improvisational music

therapy on depression in adolescents and adults with substance abuse: a randomized controlled trial. Nordic Journal of Music Therapy 2011;20(3):208-24. [DOI: 10.1080/08098131.2010.522717]

*  Albornoz Y. The Effects of Group Improvisational Music Therapy on Depression in Adolescents and Adults with Substance Abuse [Doctoral dissertation]. Philadelphia (PA): Temple University, 2009.

Eshaghi Farahmand 2020 {published data only} Eshaghi Farahmand S, Ahadi H, Kalhornia

Golkar M, Sedaghat M. Comparison of the effectiveness of music therapy and cognitive behavioral therapy on quality of life, craving and emotion regulation in patients under methadone maintenance therapy. Iranian Journal of Psychiatry and Clinical Psychology 2020;26(2):170-87. [DOI: 10.32598/ijpcp.26.2.3212.1]

Heiderscheit 2005 {published data only} Heiderscheit A. The effects of the Bonny Method of

Guided Imagery and Music (GIM) on interpersonal problems, sense of coherence, and salivary immunoglobulin A of adults in chemical dependency treatment. Music & Medicine 2017;9(1):24-36.

*  Heiderscheit AL. The Effects of the Bonny Method of Guided Imagery and Music (GIM) on Adults in Chemical Dependency Treatment: Sense of Coherence, Salivary Immunoglobulin A and Interpersonal problems [Doctoral dissertation]. Minneapolis (MN): University of Minnesota, 2005. [UNIVERSITY OF MINNESOTA DIGITAL CONSERVANCY: hdl.handle.net/11299/160300]

James 1988 {published data only} James MR. Music therapy values clarification: a positive

influence on perceived locus of control. Journal of Music Therapy 1988;25(4):206-15.

Murphy 2008 {published data only} Murphy KM. The Effects of Group Guided Imagery and

Music on the Psychological Health of Adults in Substance Abuse Treatment [Doctoral dissertation]. Philadelphia (PA): Temple University, 2008.

Silverman 2009 {published data only} Silverman MJ. The effect of lyric analysis on treatment

eagerness and working alliance in consumers who are in detoxification: a randomized clinical effectiveness study. Music Therapy Perspectives 2009;27(2):115-21. [DOI: 10.1093/mtp/27.2.115]

Silverman 2010 {published data only} Silverman MJ. The effect of a lyric analysis intervention

on withdrawal symptoms and locus of control in patients on a detoxification unit: a randomized effectiveness study. Arts in Psychotherapy 2010;37(3):197-201. [DOI: 10.1016/ j.aip.2010.04.001]

Silverman 2011a {published data only} Silverman MJ. Effects of music therapy on change and

depression on clients in detoxification. Journal of Addictions Nursing 2011;22(4):185-92. [DOI: 10.3109/10884602.2011.616606]

Silverman 2011b {published data only} Silverman MJ. Effects of music therapy on change

readiness and craving in patients on a detoxification unit. Journal of Music Therapy 2011;48(4):509-31. [DOI: 10.1093/jmt/48.4.509]

Silverman 2012 {published data only} Silverman MJ. Effects of group songwriting on

motivation and readiness for treatment on patients in detoxification: a randomized wait-list effectiveness study. Journal of Music Therapy 2012;49(4):414-29. [DOI: 10.1093/jmt/49.4.414]

Silverman 2014 {published data only} Silverman MJ. Effects of music therapy on drug

avoidance self-efficacy in patients on a detoxification unit: a three-group randomized effectiveness study. Journal of Addictions Nursing 2014;25(4):172-81. [DOI: 10.1097/JAN.0000000000000047]

Silverman 2015a {published data only} Silverman MJ. Effects of educational music therapy on

knowledge of triggers and coping skills, motivation, and treatment eagerness in patients on a detoxification unit: a three-group cluster-randomized effectiveness study. Korean Journal of Music Therapy 2015;17(2):81-101. [DOI: 10.21330/ kjmt.2015.17.2.81]

Silverman 2015b {published data only} Silverman MJ. Effects of lyric analysis interventions on

treatment motivation in patients on a detoxification unit: a randomized effectiveness study. Journal of Music Therapy 2015;52(1):117-34. [DOI: 10.1093/jmt/thu057]

Silverman 2016a {published data only} Silverman MJ. Effects of live and educational music

therapy on working alliance and trust with patients on detoxification unit: a four-group cluster-randomized trial. Substance Use & Misuse 2016;51(13):1741-50. [DOI: 10.1080/10826084.2016.1197263]

Silverman 2016b {published data only} Silverman MJ. Effects of a single lyric analysis

intervention on withdrawal and craving with inpatients on a detoxification unit: a cluster-randomized effectiveness study. Substance Use & Misuse 2016;51(2):241-9. [DOI: 10.3109/10826084.2015.1092990]

Silverman 2017 {published data only}

*  Silverman MJ.Effects of group-based educational songwriting on craving in patients on a detoxification unit: a cluster-randomized effectiveness study. Psychology of Music 2017;Advance online publication:1-14. [DOI: 10.1177/0305735617743103]

Silverman MJ.Effects of group-based educational songwriting on craving in patients on a detoxification unit: a cluster-randomized effectiveness study. Psychology of Music 2019;47(2):241-54. [DOI: 10.1177/0305735617743103]

Silverman 2019a {published data only}

*  Silverman MJ. Quantitative comparison of group-based music therapy experiences in adults with substance use disorder on a detoxification unit: a three-group cluster-randomized study. Arts & Health 2019; Advance online publication:1-14. [DOI: 10.1080/17533015.2019.1608568]

Silverman MJ. Quantitative comparison of group-based music therapy experiences in adults with substance use disorder on a detoxification unit: a three-group cluster-randomized study. Arts & Health 2021;13(1):49-62. [DOI: 10.1080/17533015.2019.1608568]

Silverman 2019b {published data only} Silverman MJ. Songwriting to target state shame,

guilt, and pride in adults with substance use disorder on a detoxification unit: a cluster-randomized study. Substance Use & Misuse 2019;54(8):1345-54. [DOI: 10.1080/10826084.2019.1580742]

Silverman 2020 {published data only} Silverman MJ. Therapeutic songwriting for perceived

stigma and perceived social support in adults with substance use disorder: a cluster-randomized effectiveness study. Substance Use & Misuse 2020;55(5):763-71. [DOI: 10.1080/10826084.2019.1701037]

Silverman 2021 {published data only} Silverman MJ. A randomized study of lyric analysis on

recognition, taking steps, and commitment to sobriety in adults on a detoxification unit. Journal of Substance Use 2021; Advance online publication:1-7. [DOI: 10.1080/14659891.2021.1879292]

Wu 2020 {published data only} Wu Q, Chen T, Wang Z, Chen S, Zhang J, Bao J, et al.

Effectiveness of music therapy on improving treatment motivation and emotion in female patients with methamphetamine use disorder: a randomized controlled trial. Substance Abuse 2020;41(4):493-500. [DOI: 10.1080/08897077.2019.1675117]

**INSOMNIA**

Amiri 2019 {published data only} Amiri S, Parvizi Fard A, Khaledi-Paveh B, Foroughi A,

Bavafa A, Bazani M, et al. The effectiveness of music therapy on insomnia using Persian traditional music. Journal of Kermanshah University of Medical Sciences 2019;23(2):e86914. [DOI: 10.5812/ jkums.86914]

Burrai 2020 {published data only} Burrai F, Sanna GD, Moccia E, Morlando F, Cosentino ER,

Bui V, et al. Beneficial effects of listening to classical music in patients with heart failure: a randomized controlled trial. Journal of Cardiac Failure 2020;26(7):541-9. [DOI: 10.1016/ j.cardfail.2019.12.005] [PMID: 31877362]

Cai 2015 {published data only} Cai X-M, Zhang X-P, Tang X. Observation on clinical effect of

auricular acupoint sticking plus music therapy for poststroke insomnia. Journal of Acupuncture and Tuina Science 2015;13(4):227-31. [DOI: 10.1007/s11726-015-0858-2]

Chang 2012 {published data only} Chang E-T, Lai H-L, Chen P-W, Hsieh Y-M, Lee L-H. The

effects of music on the sleep quality of adults with chronic insomnia using evidence from polysomnographic and selfreported analysis: a randomized control trial. International Journal of Nursing Studies 2012;49(8):921-30. [DOI: 10.1016/ j.ijnurstu.2012.02.019] [PMID: 22494532]

Harmat 2008 {published and unpublished data} Harmat L, Takács J, Bódizs R. Music improves

sleep quality in students. Journal of Advanced Nursing 2008;62(3):327-35. [DOI: 10.1111/j.1365-2648.2008.04602.x] [PMID: 18426457]

Huang 2017 {published data only} Huang C-Y, Chang E-T, Hsieh Y-M, Lai H-L. Effects of music and music video interventions on sleep quality: a randomized controlled trial in adults with sleep disturbances. Complementary Therapies in Medicine 2017;34:116-22. [DOI: 10.1016/j.ctim.2017.08.015] [PMID: 28917363]

Jespersen 2019 {published data only}

*  Jespersen KV, Otto M, Kringelbach M, van Someren E, Vuust P. A randomized controlled trial of bedtime music for insomnia disorder. Journal of Sleep Research 2019;28(4):e12817. [DOI: 10.1111/jsr.12817] [PMID: 30676671]

NCT02321826. Music for insomnia [Better night – better day: a randomized controlled trial of listening to music for improving insomnia]. clinicaltrials.gov/ct2/show/NCT02321826 (first received 22 December 2014).

Kullich 2003 {published and unpublished data} Kullich W, Bernatzky G, Hesse HP, Wendtner F,

Likar R, Klein G. Music therapy – effect on pain, sleep, and quality of life in low back pain [Musiktherapie – Wirkung auf Schmerz, Schlaf und Lebensqualität bei low back pain]. Wiener Medizinische Wochenschrift. 2003;153(9-10):217-21. [DOI: 10.1046/ j.1563-258x.2003.02081.x] [PMID: 12836459]

Lai 2005 {published data only}

*  Lai H-L, Good M. Music improves sleep quality in older adults. Journal of Advanced Nursing 2005;49(3):234-44. [DOI: 10.1111/ j.1365-2648.2004.03281.x] [PMID: 15660547]

Lai H-L. The Effects of Music Therapy on Sleep Quality in Elderly People [PhD thesis]. Ann Arbor (MI): UMI Dissertation Services, 2004.

Liu 2016 {published data only} Liu Y-H, Lee CS, Yu C-H, Chen C-H. Effects of music listening on

stress, anxiety, and sleep quality for sleep-disturbed pregnant women. Women & Health 2016;56(3):296-311. [DOI: 10.1080/03630242.2015.1088116] [PMID: 26361642]

Momennasab 2018 {published data only} Momennasab M, Ranjbar M, Najafi SS. Comparing

the effect of listening to music during hemodialysis and at bedtime on sleep quality of hemodialysis patients: a randomized clinical trial. European Journal of Integrative Medicine 2018;17:86-91. [DOI: 10.1016/j.eujim.2017.12.001]

Shum 2014 {published data only} Shum A, Taylor BJ, Thayala J, Chan MF. The effects of

sedative music on sleep quality of older community-dwelling adults in Singapore. Complementary Therapies in Medicine 2014;22(1):49-56. [DOI: 10.1016/j.ctim.2013.11.003] [PMID: 24559816]

Wang 2016 {published data only} Wang Q, Chair SY, Wong EM, Li X. The effects of music

intervention on sleep quality in community-dwelling elderly. Journal of Alternative and Complementary Medicine 2016;22(7):576-84. [DOI: 10.1089/acm.2015.0304] [PMID: 27223689]

**DEMENTIA**

Ceccato 2012 {published data only} Ceccato E, Vigato G, Bonetto C, Bevilacqua A, Pizziolo P,

Crociani S, et al. STAM protocol in dementia: a multicenter, single-blind, randomized, and controlled trial. American Journal of Alzheimer's Disease and Other Dementias 2012;27(5):301-10.

Cho 2016 {published data only} Cho HK. The Effects of Group Singing on Quality of Life and

Affect of People with Dementia: a Randomized Controlled Trial [Dissertation]. Ann Arbor (MI): ProQuest Dissertations Publishing, 2016.

Clark 1998 {published data only} Clark ME, Lipe AW, Bilbrey M. Use of music to decrease

aggressive behaviors in people with dementia. Journal of Gerontological Nursing 1998;24(7):10-7.

Cooke 2010 {published data only}

*  Cooke M, Moyle W, Shum D, Harrison S, Murfield J. A randomized controlled trial exploring the effect of music on quality of life and depression in older people with dementia. Journal of Health Psychology 2010;15(5):765-76. [DOI: 10.1177/1359105310368188]

*  Cooke ML, Moyle W, Shum DH, Harrison SD, Murfield JE. A randomized controlled trial exploring the effect of music on agitated behaviours and anxiety in older people with dementia. Aging & Mental Health 2010;14(8):905-16. [DOI: 10.1080/13607861003713190]

Harrison S, Cooke M, Moyle W, Shum D, Murfield J. Development of a music intervention protocol and its effect on participant engagement: experiences from a randomised controlled trial with older people with dementia. Arts & Health 2010;2(2):125-39.

Murfield J, Cooke M, Moyle W, Shum D, Harrison S. Conducting randomized controlled trials with older people with dementia in long-term care: challenges and lessons learnt. International Journal of Nursing Practice 2011;17(1):52-9. [DOI: 10.1111/ j.1440-172X.2010.01906.x]

Guétin 2009 {published data only} Guétin S, Portet F, Picot MC, Pommie C, Messaoudi M,

Djabelkir L, et al. Effect of music therapy on anxiety and depression in patients with Alzheimer's type dementia: randomised, controlled study. Dementia and Geriatric Cognitive Disorders 2009;28(1):36-46. [DOI: 10.1159/000229024]

Hsu 2015 {published data only} Hsu MH, Flowerdew R, Parker M, Fachner J, Odell-Miller H.

Individual music therapy for managing neuropsychiatric symptoms for people with dementia and their carers: a cluster randomised controlled feasibility study. BMC Geriatrics 2015;15:84. [DOI: 10.1186/s12877-015-0082-4]

Liesk 2015 {published data only} Liesk J, Hartogh T, Kalbe E. Cognitive stimulation and music

intervention for people with dementia in nursing homes: a pilot study, problems and perspectives [Kognitive stimulation und musikintervention bei stationär versorgten menschen mit demenz: eine pilotstudie, probleme und perspektiven]. Zeitschri- für Gerontologie und Geriatrie 2015;48(3):275-81.

Lin 2011 {published data only} Chu H, Yang CY, Lin Y, Ou KL, Lee TY, O'Brien AP, et al. The

impact of group music therapy on depression and cognition in elderly persons with dementia: a randomized controlled study. Biological Research for Nursing 2014;16(2):209-17. [DOI: 10.1177/1099800413485410]

Lin Y, Chu H, Yang CY, Chen CH, Chen SG, Chang HJ, et al. Effectiveness of group music intervention against agitated behavior in elderly persons with dementia. International Journal of Geriatric Psychiatry 2011;26(7):670-8. [DOI: 10.1002/ gps.2580]

Lord 1993 {published data only} Lord TR, Garner JE. Effects of music on Alzheimer patients.

Perceptual and Motor Skills 1993;76(2):451-5.

Lyu 2014 {published data only} Lyu J, Gao T, Li M, Xie L, Li W, Jin W, et al. The effect of music

therapy on memory, language and psychological symptoms of patients with mild Alzheimer's disease. Chinese Journal of Neurology 2014;47(12):831-5.

Narme 2012-study 1 {published data only} Narme P, Tonini A, Khatir F, Schiaratura L,

Clément S, Samson S. Non pharmacological treatment for Alzheimer's disease: comparison between musical and non-musical interventions [Thérapies non médicamenteuses dans la maladie d'Alzheimer: comparaison d'ateliers musicaux et non musicaux]. Gériatrie et Psychologie Neuropsychiatrie du Vieillissement 2012;10(2):215-24.

Narme 2012-study 1a {published data only} Narme P, Tonini A, Khatir F, Schiaratura L,

Clément S, Samson S. Non pharmacological treatment for Alzheimer's disease: comparison between musical and non-musical interventions [Thérapies non médicamenteuses dans la maladie d'Alzheimer: comparaison d'ateliers musicaux et non musicaux]. Gériatrie et Psychologie Neuropsychiatrie du Vieillissement 2012;10(2):215-24. [In the article, this is referred to as study 2]

Narme 2014 {published data only}

*  Narme P, Clément S, Ehrlé N, Schiaratura L, Vachez S, Courtaigne B, et al. Efficacy of musical interventions in dementia: evidence from a randomized controlled trial. Journal of Alzheimer's Disease 2014;38(2):359-69. [DOI: 10.3233/ JAD-130893]

Samson S, Clément S, Narme P, Schiaratura L, Ehrlé N. Efficacy of musical interventions in dementia: methodological requirements of nonpharmacological trials. Annals of the New York Academy of Sciences 2015;1337:249-55. [DOI: 10.1111/ nyas.12621]

Raglio 2010a {published data only}

Raglio A, Bellelli G, Traficante D, Gianotti M, Ubezio MC, Gentile S, et al. Addendum to 'Efficacy of music therapy treatment based on cycles of sessions: a randomised controlled trial' (Raglio et al., 2010). Aging & Mental Health 2012;16(2):265-7. [DOI: 10.1080/13607863.2011.630376]

*  Raglio A, Bellelli G, Traficante D, Gianotti M, Ubezio MC, Gentile S, et al. Efficacy of music therapy treatment based on cycles of sessions: a randomised controlled trial. Aging & Mental Health 2010;14(8):900-4. [DOI: 10.1080/13607861003713158]

Raglio 2010b {published data only} Raglio A, Oasi O, Gianotti M, Manzoni V, Bolis S,

Ubezio MC, et al. Effects of music therapy on psychological symptoms and heart rate variability in patients with dementia: a pilot study. Current Aging Science 2010;3(3):242-6.

Raglio 2015 {published data only} Raglio A, Bellandi D, Baiardi P, Gianotti M, Ubezio MC,

Zanacchi E, et al. Effect of active music therapy and individualized listening to music on dementia: a multicenter randomized controlled trial. Journal of the American Geriatrics Society 2015;63(8):1534-9.

Ridder 2013 {published data only} Ridder HM, Stige B, Qvale LG, Gold C. Individual music

therapy for agitation in dementia: an exploratory randomized controlled trial. Aging & Mental Health 2013;17(6):667-78.

Sakamoto 2013 {published data only} Sakamoto M, Ando H, Tsutou A. Comparing the effects

of different individualized music interventions for elderly individuals with severe dementia. International Psychogeriatrics / IPA 2013;25(5):775-84.

Sung 2012 {published data only} Sung HC, Lee WL, Li TL, Watson R. A group music

intervention using percussion instruments with familiar music to reduce anxiety and agitation of institutionalized older adults with dementia. International Journal of Geriatric Psychiatry 2012;27(6):621-7.

Svansdottir 2006 {published data only} Svansdottir HB, Snaedal J. Music therapy in moderate

and severe dementia of Alzheimer's type: a case-control study. International Psychogeriatrics / IPA 2006;18(4):613-21.

Thornley 2016 {published data only} Thornley J, Hirjee H, Vasudev A. Music therapy in

patients with dementia and behavioral disturbance on an inpatient psychiatry unit: results from a pilot randomized controlled study. International Psychogeriatrics 2016;28(5):869-71.

Vink 2013 {published data only}

Vink AC, Zuidersma M, Boersma F, de Jonge P, Zuidema SU, Slaets JP. Effect of music therapy versus recreational activities on neuropsychiatric symptoms in elderly adults with dementia: an exploratory randomized controlled trial. Journal of the American Geriatrics Society 2014;62(2):392-3. [DOI: 10.1111/ jgs.12682]

*  Vink AC, Zuidersma M, Boersma F, de Jonge P, Zuidema SU, Slaets JP. The effect of music therapy compared with general recreational activities in reducing agitation in people with dementia: a randomised controlled trial. International Journal of Geriatric Psychiatry 2013;28(10):1031-8. [DOI: 10.1002/ gps.3924]
